# Development of quantitative screen for 1550 chemicals with GC-MS

**DOI:** 10.1007/s00216-018-0997-7

**Published:** 2018-03-19

**Authors:** Alan J. Bergmann, Gary L. Points, Richard P. Scott, Glenn Wilson, Kim A. Anderson

**Affiliations:** Department of Environmental and Molecular Toxicology, Oregon State University, 1007 Agricultural and Life Sciences Bldg., Corvallis, OR 97331 USA

**Keywords:** Gas chromatography, Multiple linear regression, Chemometrics, Response prediction, Automated mass spectral deconvolution and identification system (AMDIS), Passive sampling devices

## Abstract

With hundreds of thousands of chemicals in the environment, effective monitoring requires high-throughput analytical techniques. This paper presents a quantitative screening method for 1550 chemicals based on statistical modeling of responses with identification and integration performed using deconvolution reporting software. The method was evaluated with representative environmental samples. We tested biological extracts, low-density polyethylene, and silicone passive sampling devices spiked with known concentrations of 196 representative chemicals. A multiple linear regression (*R*^2^ = 0.80) was developed with molecular weight, logP, polar surface area, and fractional ion abundance to predict chemical responses within a factor of 2.5. Linearity beyond the calibration had *R*^2^ > 0.97 for three orders of magnitude. Median limits of quantitation were estimated to be 201 pg/μL (1.9× standard deviation). The number of detected chemicals and the accuracy of quantitation were similar for environmental samples and standard solutions. To our knowledge, this is the most precise method for the largest number of semi-volatile organic chemicals lacking authentic standards. Accessible instrumentation and software make this method cost effective in quantifying a large, customizable list of chemicals. When paired with silicone wristband passive samplers, this quantitative screen will be very useful for epidemiology where binning of concentrations is common.

**Electronic supplementary material:**

The online version of this article (10.1007/s00216-018-0997-7) contains supplementary material, which is available to authorized users.

**Graphical abstract:**
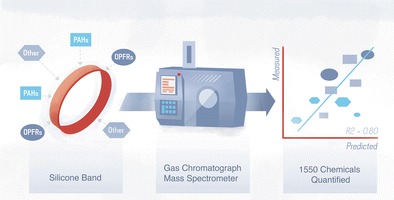
A multiple linear regression of chemical responses measured with GC-MS allowed quantitation of 1550 chemicals in samples such as silicone wristbands.

## Introduction

Hundreds of thousands of chemicals exist in the environment. Many chemicals are known to pose health risks and many more are yet to be evaluated. Semi-volatile organic chemicals (SVOCs) are of interest because they are often bioavailable, have potential adverse health impacts, and can be persistent in the environment. Rapid and cost-effective methods for sampling and analysis are necessary to improve our ability to monitor environmental contamination and prioritize chemicals for health research.

Current analytical techniques to identify and measure SVOCs can precisely quantify small numbers (e.g., < 100) of chemicals within specific classes of chemicals in targeted quantitation, e.g., Anderson et al. [1]. Typically, targeted gas chromatography mass spectrometry (GC-MS) methods use in-house chemical standards to develop method-specific libraries of retention times, mass spectra, and response factors. Alternatively, non-target analysis thoroughly investigates complex chromatograms for unknown components [2]. Non-target methods use high-resolution mass spectrometry and reference libraries of mass spectra such as the National Institute of Standards and Technology (NIST) Mass Spectral Library. A third approach is to perform targeted screening for a hundreds of chemicals which balances the analysis time of targeted quantitation with the thoroughness of non-target analysis.

Targeted screening methods are frequently performed on GC or liquid chromatography with mass spectrometry. GC is well suited for the analysis of SVOCs. Non-target and targeted screening are performed by capturing the full scan ion profile including potentially interfering chemicals. Deconvolution software, such as the Automated Mass Spectral Deconvolution and Identification System (AMDIS, NIST), is available to extract specific signals from complex chromatograms. A challenge with targeted screening is to develop libraries of retention times, mass spectra, and response factors for hundreds or thousands of chemicals. It is unfeasible for a laboratory to determine these parameters for each chemical individually but it can rely on libraries for mass spectra such as the NIST MS Library, and to a more limited extent, retention time. Agilent Technologies developed the Deconvolution Reporting Software (DRS) package for ChemStation to incorporate retention time indices in a standardized GC method with AMDIS and the NIST MS library [3]. This type of analysis is good for identifying chemicals in complex chromatograms but quantification requires more work.

In order to accurately quantify chemicals in targeted screening methods, response factors for a large list of chemicals need to be determined. While calibrations can be manually constructed for every chemical [4], this is time and resource intensive especially for methods with hundreds to thousands of target analytes. Another method is to assign an internal standard to groups of chemicals in the method. Bu et al. assigned 14 internal standards based on chemical class to a list of 847 chemicals analyzed by GC-MS [5, 6]. They were able to quantify within a factor of 4 the true value of target analytes in spiked sediment extracts. Differences in response factors between the internal standard and targets contributed to the uncertainty in quantification. A third method is to use chemometrics to predict instrument response from chemical properties [7]. Chemical-specific parameters that vary within chemical classes such as molecular weight, polarity, and fractional ion abundance [8, 9] affect chemical response. Some work has been done to predict chemical-specific MS responses, mostly in electrospray ionization-MS for liquid chromatography applications [10], and for GC-MS with thermal desorption for a small set of VOCs [11]. Our goal was to develop a predictive model for response factor calibration of SVOCs lacking authentic standards and to apply the method to samples with a high-throughput potential.

Passive sampling devices (PSDs) are versatile and simple tools for measuring contaminants in the environment and biota [12–14]. The polymers low-density polyethylene (LDPE) and polydimethylsiloxane (silicone) are used as PSDs to sample for SVOCs. These PSDs accumulate lipophilic chemicals from the environment through passive diffusion. Commonly deployed for days to weeks at a time, PSDs can concentrate trace contaminants and lower environmental detection limits. Consequently, environmentally deployed LDPE can contain many hundreds to thousands of individual chemicals. Wristband passive sampling devices made of silicone are a recent development in passive sampling. Silicone wristbands are used to monitor a person’s external exposure to SVOCs and can have more variable backgrounds than environmentally deployed LDPE [15, 16]. Human biomonitoring, such as with silicone wristbands, generates immense data sets. Wristband passive sampling devices can be used in large numbers because they are cheap, non-invasive, hold chemicals stably for weeks at ambient temperature, and can be prepared for deployment in large batches using minimal solvent [15]. Still, the majority of laboratory time and cost associated with wristband biomonitoring is in performing the chemical extraction and analysis.

Our objective was to create a quantitative GC-MS method for a list of more than 1500 SVOCs to pair with the high sample generation of wristbands and other PSDs. We aimed to leverage the integrated deconvolution and chemical confirmation available with the DRS package and generate a library of calibrations for chemicals that we do not physically have in the laboratory. We present the analytical and statistical results of predicting response. We called the resulting multi-class quantitative screen the Many Analyte Screen Version 1500 (MASV1500). We validated the accuracy, precision, and sensitivity of this method in real environmental samples with a focus on passive sampling devices. As the first report of a high-throughput quantitative screen using the deconvolution freeware AMDIS, this work demonstrates an analytical method that compliments the sample generating capacity of passive sampling devices.

## Methods

### Chemical standards

A complete list of 224 chemicals used for calibration model building and testing can be found in Table S1 (see Electronic Supplementary Material (ESM)). Standards were purchased from a variety of sources including AccuStandard, Sigma-Aldrich, TCI America, SantaCruz Biotechnology, and Chiron. Standards were prepared as singles or simple mixes in ethyl acetate, n-hexane, or isooctane (Fisher Scientific, optima grade) at concentrations typically between 0.5 and 10 μg/mL.

### GC-MS parameters

All data was acquired using an Agilent 7890A GC coupled with an Agilent 5975C MSD operated in in full scan mode with electron ionization. The GC was equipped with an Agilent DB-5MS column (30 m × 0.25 mm). The inlet pressure was locked to the retention time of chlorpyrifos at 19.23 (± 0.20) minutes. Full details of the instrument operating parameters are available in Table S2 (see ESM).

### Deconvolution

AMDIS version 2.66 (NIST), as part of the DRS (Agilent) was used to deconvolute and identify all peaks. All AMDIS software parameters are given in Table S3 (see ESM). AMDIS integrations of deconvoluted peaks were used for quantification.

### Adding chemicals to libraries

The complete chemical library consisted of 1550 chemicals including 21 chemicals that are isotopically labeled or otherwise not generally found in the environment. We achieved this list by manually adding approximately 450 chemicals to libraries purchased with the deconvolution software. Single-component standards were prepared from neat or purchased in ethyl acetate, methylene chloride, n-hexane, or isooctane at a concentration between 0.5 and 10 ng/μL. Mass spectra and retention time for each new chemical were acquired using the GC-MS method described above and added to a new ChemStation probability-based matching library. Each entry included chemical name, retention time, retention index (retention time in seconds), mass spectra, molecular formula (from which the software generates MW) and Chemical Abstracts Service registration number (CASRN). After new chemicals were added, the new library was appended to the master library. AMDIS library files and an updated method file were then generated from this master library using ChemStation software.

### Initial calibration

In order to calibrate for 1550 chemicals, we developed a predictive model based on the response factors of chemicals available in our laboratory. A modeling set of 196 chemicals was analyzed. The modeling set consisted of PAHs, several classes of pesticides, polychlorinated biphenyls (PCBs), polybrominated diphenylethers (PBDEs), and phosphate flame retardants, phenols, and anilines (Table S1, see ESM).

Molecular weight (MW), topical polar surface area (PSA), Henry’s law, octanol-water partitioning coefficient (logP), octanol-air partitioning coefficient (Koa), acid disassociation constants (pKa), halogen and heteroatom substitution abundance, and fractional ion abundance were examined as potential explanatory variables. These parameters were obtained from Advanced Chemistry Development (ACD) Labs through Chemspider (R version 3.3, webchem package). For chemicals that were not available through Chemspider (*n* = 97), we manually retrieved the parameters from Episuite 4.1 Software or from ChemStation and AMDIS libraries. Fractional ion abundance was obtained from the AMDIS spectral library and is calculated as the ratio of the most abundant ion to the sum of all ion abundances. Python (version 3.6) code used to calculate fractional ion abundance from the AMDIS library files and to pull values from Chemspider can be found in the ESM. We assessed the representativeness of the modeling set by comparing the distributions of the final selected chemical parameters to the entire 1550 list (Kolmogorov-Smirnov test, alpha = 0.05).

The responses of triplicate injections at 500 pg/μL were used to construct a multiple linear regression (JMP Pro 13) to predict responses at that concentration based on chemical parameters. Chemicals in the modeling set were randomly assigned to either a training set (75%, 147 chemicals) or a test set (25%, 49 chemicals). The distributions of the GC-MS response and explanatory variables were first evaluated for the need for transformation. Many parameters that were not already in logarithmic scale (e.g., logP and pKa) were left-centered. To normally distribute these data, log_10_ (log) transformations were applied. Model optimization proceeded through forward and backward stepwise regression to maximize the adjusted *R*^2^ while minimizing root mean square error (RMSE) and minimizing the Akaike information criterion. We interpreted RMSE as an estimate of the standard deviation of the model residuals. Assuming that two standard deviations plus and minus the mean encompass 95% of the observations, the untransformed precision of the calibration model is given by 10^(RMSE*2)^.

The equation of the optimized multiple linear regression used to predict the response of a given chemical at 500 pg/μL is:

1 $$ \mathit{\log}\left(500\  pg/\mu L\  Predicted\ Response\right)=9.372+\left[0.05678\ast logP\right]+\left[0.7394\ast \mathit{\log}\left( fractional\  ion\  abundance\right)\right]-\left[1.169\ast \mathit{\log}(MW)\right]-\left[0.173\ast \mathit{\log}\left( PSA+1\right)\right]+\left[\left( logP-5.045\right)\ast \left(\left(\mathit{\log}(MW)-2.432\right)\ast \left(-0.2466\right)\right)\right] $$

Raw instrument responses were directly divided by response factors to estimate concentration in picogram per microliter as given by:

2 $$ Concentration=\frac{Response\times 500\  pg/\mu L}{{Predicted\ Response}_{500\  pg/ uL}} $$

### Method performance

We evaluated the calibrated method with an overspike solution of 112 chemicals in isooctane (Table S1, see ESM) that represent a range of physico-chemical properties from chemical classes including pesticides, PAHs, phenols, and anilines. With clean standard solutions at several concentrations, and matrix-matched overspikes, we determined method linearity, precision, accuracy, and estimated limits of quantitation.

### Linearity

Because our prediction model was designed using only a single concentration level, we performed experiments to evaluate the range of concentration over which it could be applied. We measured response of 112 chemicals in the overspike solution at 100, 500, 2500, and 10,000 pg/μL in isooctane. The accuracy of the response prediction was evaluated at each concentration level and the adjusted *R*^2^ was used to evaluate the linear response range of each modeled chemical.

### Inter-instrument

Method transferability was tested by running the same method on a second GC-MS with identical parameters. The method calibration verification (CV) standards described in the quality control section were evaluated on two GC-MS instruments on the same day.

### Matrix overspikes

Typical samples that we anticipate analyzing with the method described in this paper include biological tissue extracts [14], LDPE passive sampling device extracts deployed in river water [17], and silicone wristbands that were worn by people [15, 18]. We evaluated the detection rate and quantitative accuracy and precision of 112 chemicals added to samples representative of typical background matrices. Silicone wristbands tend to have variable and high backgrounds of silicone, fatty acids, and steroidal chemicals (cholesterol, squalene). Five people wore a silicone wristband for 5 days to generate samples with background matrices typical of deployed wristbands. The samples were randomized and no personal information was collected which might be used to identify the participants. Aliquots of the five deployed wristbands were cleaned with C18 solid-phase extraction as described elsewhere [19]. Both pre- and post-SPE-cleaned wristband samples were evaluated. We also tested the overspike solution in one crayfish extract [14], one pre-deployment LDPE, one pre-deployment wristband, and four deployed LDPE [17].

### Instrumental limit of quantitation

Instrumental limits of quantitation (LOQs) were calculated in accordance with other methods in our laboratory [1, 20] and as described by the U.S. EPA [21]. Several adjustments were made to this method to account for modeling chemical responses rather than measuring them directly. The previously described overspike mixture (112 chemicals) was prepared at 500 pg/μL and was injected seven times to assess instrument variability. For each chemical with at least three detections by AMDIS, the standard deviation of the responses was calculated and multiplied by a single-tailed Student *t* value with the appropriate degrees of freedom (d.f. = *n* − 1; where *n* was the number of times that the chemical was identified). The average adjusted standard deviation for all evaluated chemicals was used in Eq. 2 to estimate LOQ for 1550 chemicals. We refer to this method of calculating LOQ as average response variation.

We also estimated LOQs using a second method, termed linear extrapolation, and compared the two. For all chemicals used to evaluate linearity that were detected in at least three of the four concentrations measured (*n* = 64), we extrapolated to the *x*-intercept for each chemical and used that value as a prediction of LOQ.

### Quality control

An analyst evaluated all chromatograms processed with AMDIS for identification and integration quality. Chromatographic peaks that did not meet data quality objections were rejected, and positive identifications that were poorly integrated by AMDIS were flagged as poor AMDIS peaks (PAP) to indicate that quantification would not be reliable. Our minimum data quality objectives for peak identification required retention times shifted by no more than 45 s and at least one qualifier ion should be within 20% of its predicted abundance relative to the quantitation ion. The AMDIS match factor threshold was set to 60 (out of 100) and the extracted spectra of identified chemicals were reviewed manually for missing or extra *m*/*z* peaks.

CVs were prepared to monitor instrument conditions. To establish target responses and acceptable deviation for the CV, 15 diverse chemicals were monitored in 12 injections over the course of several days. An average response was taken for each chemical across all injections in which a chemical was identified and not flagged as a PAP. These responses were used in Eq. 2 to give target concentrations for our CVs.

Instrument blanks (clean ethyl acetate or hexane) and CVs were evaluated before and after every sample set analyzed with this method. To meet our suggested data quality objectives, no target chemicals should be identified in the instrument blanks and greater than 70% of CV target chemicals must be within 30% of the responses given in Table S4 (see ESM). The CV included diagnostic chemicals to inform about instrument condition [6]. For example p,p′-DDT degradation to p,p′-DDE or p,p′-DDD indicates contamination at the GC inlet [22]. Method QC samples can contain some target chemicals. Specifically, phthalates are regularly identified as background in undeployed PSD matrices of both LDPE and silicone. However, the amounts of these pervasive chemicals in deployed samples are typically 100–10,000 times greater than QC samples [18].

One of the challenges encountered at the outset of this project was data curation. Purchased libraries often contained errors in chemical names and/or CAS numbers. We cross-checked the library entries for accuracy using R, Python, and JMP. Several chemicals in purchased libraries also lacked any retention time data. These errors were corrected when possible with individual standards but there may be additional errors in purchased retention time data that could not be identified without obtaining all standards.

## Results and discussion

### Calibration

We calibrated for chemicals lacking authentic standards by predicting response factors based on physico-chemical properties of model chemicals. As a predictive model, our goal was not to interpret the explanatory variables but to optimize the precision and accuracy of the model predictions. However, we chose to test physico-chemical properties based on (1) potential to affect GC-MS response and (2) availability to the average user. After model optimization, we determined that MW, logP, fractional ion abundance, and PSA were good predictors of response factor.

Chemicals in the domain of the calibration model are organic chemicals that are measurable by GC-MS. Specifically, the ranges of each parameter for all chemicals in the method are MW 93 to 793 g/mol; fractional ion abundance 0.0223 to 0.7849; logP − 3.77 to 10.14; and PSA 0 to 202 Å^2^. These predictors were generally representative of the entire list (Fig. S1, see ESM). The distributions of log(MW) and log(fractional ion abundance) in the model set were not significantly different from the non-modeled chemicals (Kolmogorov-Smirnov test, *p* > 0.05). LogP and log(PSA + 1) showed evidence that the distributions were not equal (Kolmogorov-Smirnov test, *p* < 0.001).

MW and fractional ion abundance were the most significant model parameters. MW was negatively associated with MS response because the detector measures an analyte’s molarity, rather than mass concentration which are the units of the calibration. At the same concentration, fewer molecules of a large MW chemical reach the detector, compared to a lighter chemical. The degree and pattern of fragmentation influences the area of the quantitation ion [9] and we observed that fractional ion abundance was a predictor of response. Quantitation is based on the AMDIS adjusted response of the ChemStation quantitation ion, which is the most abundant ion fragment in the mass spectrum. Chemicals that have a greater degree of fragmentation (e.g., endosulfan) may have lower response than a chemical that remains relatively intact (e.g., phenanthrene).

We observed that greater polarity as described by logP and PSA resulted in decreased response. Polar chemicals commonly have functional groups that may interact with instrument components, reducing the mass of the chemical that reaches the detector [23]. Polar chemicals are often less volatile than non-polar chemicals holding everything else constant. However, we did not observe a significant effect of Henry’s law constant or logKoa on instrument response so volatility does not seem to be directly related to response. For the same reasons, we also expected acidity of analytes to influence response but pKa was also not a significant model parameter. This is possibly because acidic chemicals (e.g. phenols, anilines) are a minority among the chemicals in the library and are very weak acids. Polarity can also influence the ionization efficiency in electrospray ionization-MS [10] and may also be important at the electron ionization source used in this study. We also observed an interaction between MW and logP. For non-polar chemicals, logP increases with MW but instrument response is negatively associated with MW and positively associated with logP. Interestingly, no interaction was observed between PSA and other parameters.

The frequency of heteroatoms and halogens (N, O, Cl, Br, I) in molecules was also evaluated as a predictor of response. It was suspected that the number and type of these atoms could be used to describe contributions to polarity and possibly fragmentation patterns. However, their contribution to the model when also including MW and PSA as explanatory variables was not significant and were worse predictors of response than MW and PSA.

**Fig. 1:**
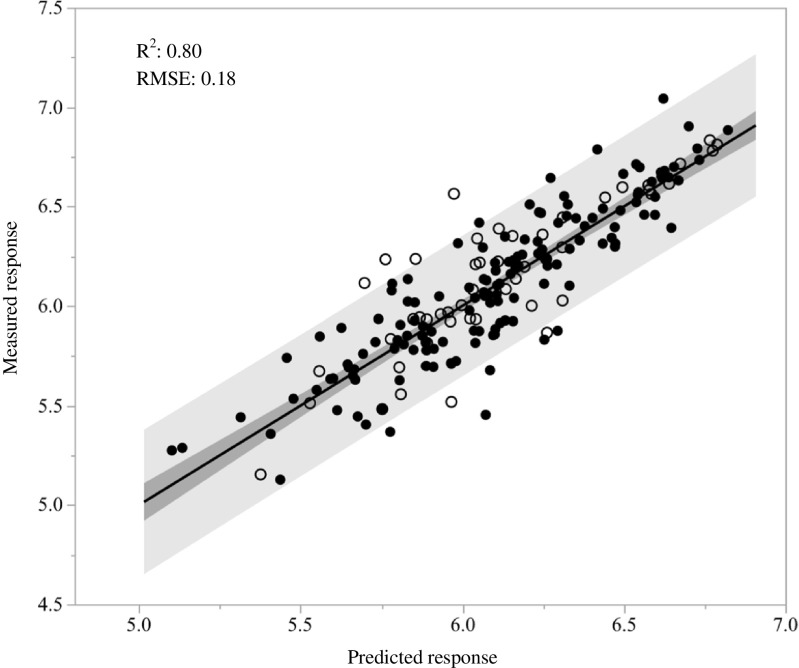
Measured vs. predicted values of 196 chemicals used in modeling GC-MS response at 500 pg/μL. Axes are log_10_ transformed. Model explanatory variables were log(MW), log(fractional ion abundance), logP, log(PSA + 1), and log(MW) crossed with logP. Training set chemicals (closed circles) and test set chemicals (open circles). Solid line is the model fit of the training set, dark shading is the fit 95% confidence interval, and light gray shading is the 95% prediction interval. *R*^2^ and root mean square error (RMSE) are given for the model fit of the training set

The final optimized model had an adjusted *R*^2^ of 0.80 and RMSE of 0.18 (Fig. 1). The RMSE translates to 95% of measured responses were within a factor of 2.28 of the true value. The model test and training sets had similar distributions of residuals with standard deviations of 0.20 and 0.18, respectively. Training and test set evaluation is commonly used for evaluation of predictive models. Steyerberg et al. also recommend bootstrapping of the models, but found that training/test set validation and bootstrapping gave similar results when events per variable were approximately 40 or more [24]. The events per variable in the current study were 39 (196 observations/5 variables). We also evaluated prediction error with a leave-one-out approach using the prediction error sum of squares (Press) statistic. The Press RMSE was 0.19. We used the most conservative estimate of prediction error, 0.20 from the test set, to determine the precision of prediction as a factor of 2.5.

The final model predicted the response factor for 95% of chemicals within a factor of 2.5 of their true value. This is, to our knowledge, better than for any previously reported method of this type. Bu et al. were able to quantify organic chemicals within a factor of 4 using a set of internal standards [6]. Naturally, quantitative screens are as precise as conventionally calibrated target methods which develop response factors with standards for every chemical. The compromise of being able to quantify over a thousand chemicals within a factor of 2.5 or 4 is within variability assumed in some disciplines. Epidemiology studies may bin chemistry results into just a few groups [25], and human health risk assessment may assume uncertainty factors of 100 when calculating reference doses [26]. The silicone wristband PSDs used as examples in the present study commonly accumulate concentrations that range several orders of magnitude, much greater than the precision of the MASV1500 method [17, 20].

While the prediction of chemical concentration in the present study is less precise than traditional targeted quantification methods, the instrumental error is comparable. The method in the present study includes peak integrations by AMDIS which did not seem to add a significant variability to the measurements. For 12 replicate injections of a standard at 100 pg/μL evaluating 62 chemicals, the relative standard deviation of the average and median chemical response were 17 and 16% respectively. The entire method was also successfully transferred to an identical GC-MS. In that inter-instrument evaluation, linalool had the greatest difference between instruments. The quantitation ion for linalool was 73 *m*/*z*, smaller than most chemicals in the method. A MS tune adjustment to weight the lower end of the mass axis could reduce this increased variation.

### AMDIS performance

**Fig. 2:**
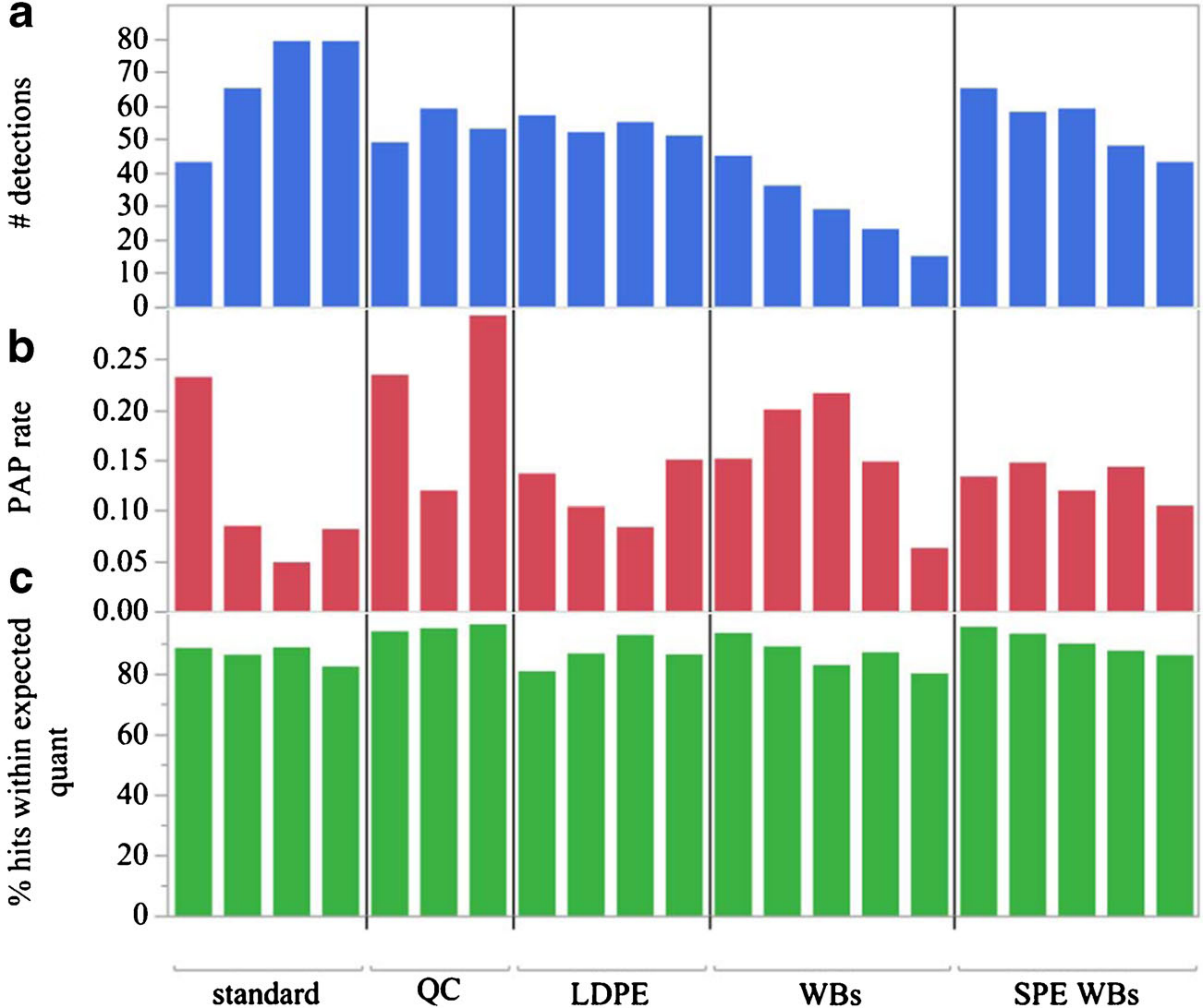
AMDIS performance in the MASV1500 method. The samples tested are shown in five groups, listed here from left to right. Standards are 100, 500, 2500, and 10,000 pg/μL overspike mixtures in ethyl acetate. QC (quality control) samples are 500 pg/μL overspike mixtures in three QC matrices: non-deployed LDPE, non-deployed wristband, and a crayfish extract. LDPE (low-density polyethylene), WBs (wristbands), and SPE WBs (solid-phase extraction cleaned wristband extracts) are replicate extracts of each matrix type with the overspike mixture at 500 pg/μL. (a) The number of positive detections by AMDIS regardless of integration quality. (b) Proportion of positive detection that were PAPs (poor AMDIS peaks). (c) The percentage of non-PAP positive detections that were quantified within the expected bounds of a factor of 2.5

**Fig. 3:**
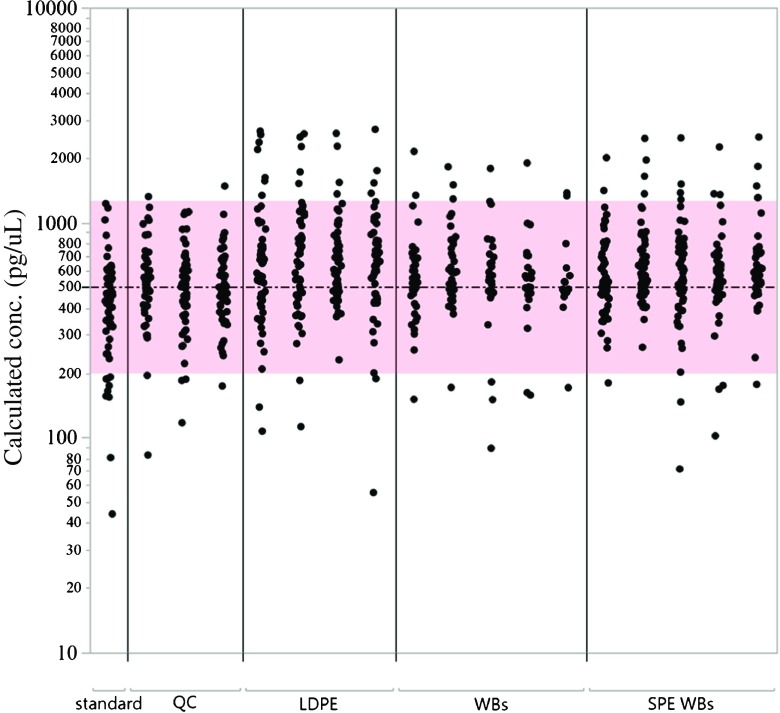
Performance of MASV1500 quantitation of matrix overspikes at 500 pg/μL. Dash-dot line is expected concentration (500 pg/μL), pink shade is ± a factor of 2.5, which is the estimated prediction error of the calibration model. Standard is 500 pg/μL in ethyl acetate. QC (quality control) samples from left to right: non-deployed LDPE, non-deployed wristband, and a crayfish extract. LDPE low-density polyethylene, WB wristband, SPE WB solid-phase extraction cleaned wristband extract

The performance of identifying and quantifying chemicals in standard solutions and matrix overspikes is shown in Figs. 2 and 3. AMDIS detected 85 of 112 chemicals (75%) among four concentrations of the overspike mixture. For chemicals that were detected in at least three levels, 64 had adjusted *R*^2^ > 0.97 (Fig. S2, see ESM). These estimates of fit indicate good linearity from 100 to 10,000 pg/μL. AMDIS detection rate and integration quality was best for clean standard solutions at 500 pg/μL and above (Fig. 2a). This is not surprising because background matrix can interfere with chemical identification, even when using deconvolution. Standards at 100 pg/μL were near the limits of quantitation and limits of detection so had lower detection rates despite no background matrix. The number of detections in matrix overspike solutions at 500 pg/μL was mostly similar to a standard solution at 500 pg/μL. An exception was deployed wristband samples before SPE clean-up which had lower and more variable number of detections than the standard solution at 500 pg/μL. The rate of PAPs was lower for high concentrations of standard solutions but varied around 10% for matrix overspike solutions (Fig. 2b). The number of positive detections that were within the expected quantitation range, excluding PAPs, was above 80% for all samples (Fig. 2c). While background matrix does not seem to affect quantitation, it does affect the number of detections in a sample (Fig. 2a). Performance of AMDIS identifications was best for high concentrations of standards in clean matrices as indicated by a high number of detections and few PAPs, followed by SPE-cleaned wristbands. AMDIS performed worst for wristbands that were not cleaned.

Figure 3 shows calculated concentrations of chemicals in spiked solutions and matrix-matched samples, and compares them to the expected precision of within a factor of 2.5 from the nominal value, between 200 and 1250 pg/uL. All standards and matrix overspikes were centered around the expected value indicating the accuracy of the method. Matrix-matched samples had more detections beyond the expected range, especially on the high end. This could be due to matrix enhancement for some chemicals, despite the method using deconvolution software.

A solution of pesticides and laboratory surrogates selected as a diverse list of chemicals was run three times at 500 pg/μL. AMDIS detected 73 out of 78 different chemicals at least once among the runs. Of these, 65 chemicals were identified by AMDIS in every replicate and 54 had RSDs less than 25%. Of the chemicals with RSDs greater than 25%, 5 out of 11 had higher variability because they were poorly integrated by AMDIS. The physico-chemical properties of the compounds evaluated for intrainstrumental variation ranged as follows: fractional ion abundance 0.0253–0.6227; MW 201–540; logP 1.19–8.1; PSA 0–171. In another test, method conditions as described above were applied to a second, identical, GC-MS. The average percent difference in response between the instruments was 13% across all chemicals identified in the CV. The percent difference ranged from 0.5% for benzothiazole to 63% for linalool which was 35% higher than any other chemical measured. Therefore, we think transferring the method would be successful.

Kadokami et al. found that the accuracy of identification by their screening method was superior to the performance of AMDIS [4]. However, they acknowledge that they did not use retention time or optimize AMDIS deconvolution settings. We found that AMDIS performed well but did not identify all chemicals. We observed that AMDIS can misidentify chemicals as their isomers within a retention window of approximately 45 s. Often, pairs or groups of isomers are present in a sample and the software will identify one peak as multiple isomers. If multiple peaks are identified as isomers but there is uncertainty about which peak is an isomer then the analyst user may choose to report all detected isomers as the sum of those chemicals.

### Limits of quantitation

A major improvement in our analysis was estimating chemical-specific LOQs. LOQs were estimated in two different ways which corroborated each other and converged on LOQs of approximately 40–500 pg/μL depending on the chemical. The median predicted LOQs were nominally the same (Fig. S3, see ESM) for both the average response variability method (201 pg/μL) and the linear extrapolation method (237 pg/μL). Additionally, the range and distribution of LOQs predicted by both models were similar. The ranges of response variability model and the linear extrapolation model were 2580 and 2393 pg/μL, respectively. Predicted LOQs for all chemicals by the response variability method can be found in Table S5 (see ESM).

The method we used to report LOQ, the average response variation method, is typically used for limit of detection (LOD) calculations. We have determined that these concentration limits are better described as LOQs than LODs because AMDIS is often able to detect chemicals at concentrations estimated below these limits but with poor performance during integration. The frequency of PAPs in a 100 pg/μL solution was much higher than any other standard (Fig. 2b), and those peaks could not be reliably integrated for quantitation.

A number of assumptions were made to determine LOQs. It is recommended to use a standard near the expected LOD when evaluating instrument variability for LOD calculations [21]. For the present method, 500 pg/μL is likely near the LOD of some chemicals in the method, but also likely to be an order of magnitude above the true LOD for many others. At 100 pg/μL, fewer chemicals were quantifiable than at 500 pg/μL because more detections were poorly integrated by AMDIS. Therefore, we chose to use 500 pg/μL to capture a greater range of chemicals, but may be overestimating the LOQ concentration for some chemicals. Additionally, we assume that the average measured response is representative of all chemicals in the model. Chemical specific response factors from the calibration model should normalize any bias from this average. Finally, all LOQ calculations have the modeling error implicitly built into them. We should therefore expect that the true LOQs may be within a factor of 2.5 from the values given here.

### Analysis time

Analysis of 1550 chemicals in one GC run saves time and cost compared to analyzing for the same chemicals in separate methods. Most traditional GC-MS methods target fewer than 50 chemicals and may take anywhere from 30 min to over an hour to acquire a chromatogram. Using conservative estimates, this would require a combined instrumentation time approaching 30 h by as many as 30 individual methods compared to 1 h for the MASV1500 method. Analyst time would increase correspondingly as it might require 10 or more minutes per method per sample, or about 5 total hours per sample. The MASV1500 method requires approximately 15 min per sample to process the chromatogram. These estimates do not include the time spent curating standard libraries, and establishing and maintaining each method.

### Limitations

GC-MS analysis is only appropriate for specific types of chemicals. The method described here does not perform well for non-volatile chemicals or very polar chemicals. Some polar chemicals (e.g. nitro-anilines, halogenated phenols) gave no response at approximately 10 ng/μL. This impacts LODs and LOQs and is a compromise for the ability to analyze for a large number of chemicals. Our linearity evaluation may be biased to good performers because only those with at least three detections among a concentration series could be included in *R*^2^ calculation.

Estimation of physico-chemical parameters can be a limiting factor when predicting accurate response factors because different sources may provide very different values [27]. Another example is that PSA method used by ACD Labs does not assign a contribution of polarity for halogen substituents. For example, all PCBs and PBDEs have PSA of 0 and 9, respectively. Different number and orientation of halogens between congeners should produce different PSA among the classes. The source of input parameters can affect model results. We chose ACD Labs as the primary source for the thoroughness of parameters available and consistency within this study. The Chemistry Dashboard from U.S. EPA is a platform for centralized chemical properties, including from ACD Labs, and offers a parameter prediction with open quantitative structure activity relationship application (OPERA) modeling [28]. Kim et al. used effective carbon number as a predictor of response for volatile organic chemicals measured with thermal desorption GC-MS [11]. We did not pursue more advanced chemical descriptors because one goal of this paper was to create an accessible method with common and easily obtainable physico-chemical parameters.

## Conclusions

The MASV1500 method described here is a targeted analysis for a large number of chemicals that can be used effectively in conjunction with non-targeted methods or sample fractionation [17, 29]. Quantitation using AMDIS and a predictive model for response factor was able to quantify multiple classes of chemicals in representative samples within a factor of 2.5, better than comparable methods that have been previously reported. This method can screen for over a thousand chemicals with less analyst time than typical methods. A different deconvolution software may offer better resolution between isomers. Other software options are available for deconvolution including a component of Agilent’s Mass Hunter [30], and many open source packages. When combined with high-throughput analysis such as with passive sampling wristband work-flows, the quantitative screen described here improves efficient environmental monitoring. Overall analysis workflow would be improved through the reduction of solvents in sample processing. To that end, thermal desorption of PSDs instead of solvent extraction could increase extraction efficiency and reduce cost of analysis.

## Electronic supplementary material

ESM 1 (PDF 1288 kb)
