# Supplementary material for: Development of quantitative screen for 1550 chemicals with GC-MS
Source: Anal Bioanal Chem. 2018 Mar 19;410(13):3101–10. doi: 10.1007/s00216-018-0997-7 (PMC5910463; doi:10.1007/s00216-018-0997-7)
Supplement: Supplementary file 1 — (PDF 1288 kb) [file 216_2018_997_MOESM1_ESM.pdf]

Analytical and Bioanalytical Chemistry

Electronic Supplementary Material

Development of quantitative screen for 1550 chemicals with GC-MS

Alan J. Bergmann, Gary L. Points, Richard P. Scott, Glenn Wilson, Kim A. Anderson

Table of Contents **Page**

Table S1. Chemicals used in initial calibration and overspike solutions. 3
Table S2. GC-MS control parameters 9
Table S3. AMDIS Parameters. 10
Figure S1. Histograms of physico-chemical properties used as parameters for the complete list of target analytes and the set used to model response. 11
Figure S2. Goodness of fit for concentration series of subset of chemicals. 12
Table S4. Continuing calibration standard target concentrations after response factor applied 13
Figure S3. Histogram of limits of quantitation (LOQs) determined by two different methods. 14
Table S5. List of all chemicals in method with model parameters & response factors at 500 pg/µL. 15
Python code for pulling fractional ion abundance from ChemStation 45
R code for obtaining chemical parameters from ACD labs via Chemspider.com 47

Table S1 Chemicals used in initial calibration and overspike solutions

CASRN	Chemical name	In overspike mix?	Used for intrainstrumental variation?	In calibration model?
2051-60-7	PCB 1	No	No	Yes
34883-43-7	PCB 8	No	No	Yes
33146-45-1	PCB 10	No	No	Yes
38444-84-7	PCB 20	No	No	Yes
55702-46-0	PCB 21	No	No	Yes
38444-90-5	PCB 37	No	No	Yes
52663-59-9	PCB 41	No	No	Yes
70362-46-8	PCB 43	No	No	Yes
62796-65-0	PCB 50	No	No	Yes
74338-24-2	PCB 55	No	No	Yes
41464-43-1	PCB 56	No	No	Yes
32598-10-0	PCB 66	No	No	Yes
32598-13-3	PCB 77	No	No	Yes
70362-50-4	PCB 81	No	No	Yes
52663-62-4	PCB 82	No	No	Yes
65510-45-4	PCB 85	No	No	Yes
68194-07-0	PCB 90	No	No	Yes
38380-01-7	PCB 99	No	No	Yes
39485-83-1	PCB 100	No	Yes	Yes
56558-16-8	PCB 104	No	No	Yes
32598-14-4	PCB 105	No	No	Yes
70424-69-0	PCB 106	No	No	Yes
74472-37-0	PCB 114	No	No	Yes
57465-28-8	PCB 126	No	No	Yes
38380-07-3	PCB 128	No	No	Yes
38411-22-2	PCB 136	No	No	Yes
35065-28-2	PCB 138	No	No	Yes
35065-27-1	PCB 153	No	No	Yes
38380-08-4	PCB 156	No	No	Yes
39635-35-3	PCB 159	No	No	Yes
32774-16-6	PCB 169	No	No	Yes
35065-30-6	PCB 170	No	No	Yes
38411-25-5	PCB 174	No	No	Yes
52663-64-6	PCB 179	No	No	Yes
35065-29-3	PCB 180	No	No	Yes
60145-23-5	PCB 182	No	No	Yes
39635-31-9	PCB 189	No	No	Yes
52663-73-7	PCB 200	No	No	Yes
2051-24-3	PCB 209	No	Yes	Yes

Table S1 (continued)

96-12-8	1,2-Dibromo-3-chloropropane	Yes	Yes	Yes
87-62-7	2,6-Dimethylaniline	No	No	Yes
2593-15-9	Terrazole	Yes	Yes	Yes
2675-77-6	Chloroneb	Yes	Yes	Yes
1918-16-7	Propachlor	Yes	Yes	Yes
13194-48-4	Ethoprophos	No	Yes	Yes
126-73-8	Tributyl phosphate	No	No	Yes
1582-09-8	Trifluralin	Yes	Yes	Yes
2303-16-4	Diallate I	Yes	Yes	Yes
319-84-6	alpha-BHC	Yes	Yes	Yes
999035-03-9	Diallate II	No	Yes	Yes
118-74-1	Hexachlorobenzene	Yes	Yes	Yes
60-51-5	Dimethoate	No	Yes	Yes
122-34-9	Simazine	No	Yes	Yes
1912-24-9	Atrazine	No	Yes	Yes
319-85-7	beta-BHC	Yes	Yes	Yes
58-89-9	Lindane	Yes	No	No
82-68-8	Pentachloronitrobenzene	Yes	Yes	Yes
944-22-9	Fonofos	No	Yes	Yes
333-41-5	Diazinon	No	Yes	Yes
319-86-8	delta-BHC	Yes	Yes	Yes
1897-45-6	Chlorothalonil	Yes	Yes	Yes
709-98-8	Propanil	No	Yes	Yes
298-00-0	Parathion-methyl	No	Yes	Yes
5598-13-0	Chlorpyrifos Methyl	No	Yes	Yes
50471-44-8	Vinclozolin	No	Yes	Yes
76-44-8	Heptachlor	Yes	Yes	Yes
15972-60-8	Alachlor	Yes	Yes	Yes
122-14-5	Fenitrothion	No	Yes	Yes
84-65-1	9,10-Anthraquinone	No	No	Yes
309-00-2	Aldrin	Yes	Yes	Yes
51218-45-2	Metolachlor	No	Yes	Yes
90-98-2	4,4'-Dichlorobenzophenone	No	Yes	Yes
2921-88-2	Chlorpyrifos	No	Yes	Yes
56-38-2	Parathion-ethyl	No	Yes	Yes
1861-32-1	Dacthal	Yes	Yes	Yes
465-73-6	Isodrin	Yes	Yes	Yes
1024-57-3	Heptachlor epoxide	Yes	Yes	Yes
40487-42-1	Pendimethalin	No	Yes	Yes
133-06-2	Captan	Yes	Yes	No
5103-74-2	gamma-Chlordane	Yes	Yes	Yes
959-98-8	Endosulfan I	Yes	Yes	Yes

Table S1 (continued)

5103-71-9	alpha-Chlordane	Yes	Yes	No
60-57-1	Dieldrin	Yes	Yes	Yes
72-55-9	4,4'-DDE	Yes	Yes	Yes
19666-30-9	Oxadiazon	No	Yes	Yes
33213-65-9	Endosulfan II	Yes	Yes	Yes
72-56-0	Perthane	Yes	Yes	Yes
510-15-6	Chlorobenzilate	Yes	Yes	Yes
5836-10-2	Chloropropylate	Yes	No	No
72-54-8	4,4'-DDD	Yes	Yes	Yes
7421-93-4	Endrin aldehyde	Yes	Yes	Yes
563-12-2	Ethion	No	Yes	Yes
1031-07-8	Endosulfan sulfate	Yes	Yes	Yes
50-29-3	4,4'-DDT	Yes	Yes	Yes
2425-06-1	Captafol	Yes	No	No
53494-70-5	Endrin ketone	Yes	Yes	Yes
732-11-6	Imidan	No	Yes	Yes
72-43-5	Methoxychlor	Yes	Yes	Yes
2385-85-5	Mirex	Yes	Yes	Yes
52645-53-1	Permethrin	Yes	Yes	Yes
999046-03-6	Permethrin II	Yes	Yes	Yes
50-32-8	benzo[a]pyrene	Yes	No	Yes
78-40-0	Triethyl phosphate	No	No	Yes
91-20-3	naphthalene	Yes	No	Yes
208-96-8	acenaphthylene	Yes	No	Yes
83-32-9	acenaphthene	Yes	No	Yes
86-73-7	Fluorene	Yes	No	Yes
877-09-8	Tetrachloro-meta-xylene	No	Yes	Yes
115-96-8	TCEP	No	No	Yes
85-01-8	phenanthrene	Yes	No	No
120-12-7	anthracene	Yes	No	Yes
85-29-0	2,4'-Dichlorobenzophenone	No	Yes	Yes
81-14-1	Musk Ketone	No	No	Yes
206-44-0	fluoranthene	Yes	No	No
120067-83-6	Fipronil-sulfide	No	Yes	Yes
120068-37-3	Fipronil	No	Yes	Yes
129-00-0	pyrene	Yes	No	Yes
39765-80-5	trans-Nonachlor	Yes	Yes	Yes
72-20-8	Endrin	Yes	Yes	Yes
120068-36-2	Fipronil-sulfone	No	Yes	Yes
56-55-3	benz[a]anthracene	Yes	No	Yes
218-01-9	chrysene	Yes	No	Yes
205-99-2	benzo[b]fluoranthene	Yes	No	No

Table S1 (continued)

193-39-5	indeno[1,2,3-cd]pyrene	Yes	No	No
53-70-3	dibenzo[a,h]anthracene	Yes	No	No
191-24-2	benzo[ghi]perylene	Yes	No	Yes
207-08-9	benzo[k]fluoranthene	Yes	No	No
115-32-2	p,p'-Dicofol	Yes	No	No
1090-13-7	5,12-Naphthacene-quinone	No	No	Yes
82-05-3	Benzanthrone	No	No	Yes
486-25-9	9-Fluorenone	No	No	Yes
5737-13-3	4H-cyclopenta[def]phenanthren-4-one	No	No	Yes
10439-39-1	9,10-Anthraquinone-d8	No	Yes	Yes
479-79-8	Benzo(a)fluoren-11-one	No	No	Yes
2498-66-0	Benz(a)anthracene-7,12-dione	No	No	Yes
3074-00-8	Naphthanthrone	No	No	Yes
91-57-6	2-methylnaphthalene	Yes	No	No
90-12-0	1-methylnaphthalene	Yes	No	Yes
575-43-9	1,6-dimethylnaphthalene	Yes	No	No
573-98-8	1,2-dimethylnaphthalene	Yes	No	Yes
132-65-0	dibenzothiophene	Yes	No	Yes
483-65-8	retene	Yes	No	Yes
2381-21-7	1-methylpyrene	Yes	No	Yes
1705-85-7	6-methylchrysene	Yes	No	Yes
191-30-0	dibenzo[a,l]pyrene	Yes	No	Yes
1718-52-1	pyrene-d10	No	Yes	Yes
613-12-7	2-methylanthracene	Yes	No	No
832-69-9	1-methylphenanthrene	Yes	No	No
939-27-5	2-Ethylnaphthalene	No	No	Yes
581-42-0	2,6-dimethylnaphthalene	Yes	No	Yes
571-58-4	1,4-dimethylnaphthalene	Yes	No	Yes
571-61-9	1,5-dimethylnaphthalene	Yes	No	No
569-41-5	1,8-dimethylnaphthalene	Yes	No	Yes
59919-41-4	2,6-diethylnaphthalene	No	No	Yes
2531-84-2	2-methylphenanthrene	Yes	No	Yes
779-02-2	9-methylanthracene	Yes	No	Yes
1576-67-6	3,6-dimethylphenanthrene	Yes	No	Yes
613-06-9	2,3-dimethylanthracene	Yes	No	Yes
781-43-1	9,10-dimethylanthracene	Yes	No	Yes
205-12-9	benzo[c]fluorene	Yes	No	Yes
27208-37-3	cyclopenta[cd]pyrene	Yes	No	Yes
57-97-6	7,12-dimethylbenz[a]anthracene	No	No	Yes
205-82-3	benzo[j]fluoranthene	Yes	No	No
192-97-2	benzo[e]pyrene	Yes	No	Yes
213-46-7	benzo[a]chrysene	Yes	No	Yes

Table S1 (continued)

191-26-4	anthanthrene	Yes	No	No
5385-75-1	dibenzo[a,e]fluoranthene	Yes	No	No
191-07-1	coronene	Yes	No	Yes
192-65-4	dibenzo[a,e]pyrene	Yes	No	No
189-55-9	dibenzo[a,i]pyrene	Yes	No	Yes
189-64-0	dibenzo[a,h]pyrene	Yes	No	Yes
217-59-4	triphenylene	Yes	No	No
238-84-6	benzo[a]fluorene	Yes	No	No
243-17-4	benzo[b]fluorene	Yes	No	No
3697-24-3	5-methylchrysene	Yes	No	No
2052-07-5	PBB 1	No	No	Yes
7025-06-1	PBDE 1	No	No	Yes
6876-00-2	PBDE 2	No	No	Yes
101-55-3	PBDE 3	No	No	Yes
51930-04-2	PBDE 10	No	No	Yes
171977-44-9	PBDE 7	No	No	Yes
189084-59-1	PBDE 12	No	No	Yes
83694-71-7	PBDE 13	No	No	Yes
2050-47-7	PBDE 15	No	No	Yes
155999-95-4	PBDE 30	No	No	Yes
189084-60-4	PBDE 32	No	No	Yes
147217-77-4	PBDE 25	No	No	Yes
41318-75-6	PBDE 28	No	No	Yes
147217-80-9	PBDE 35	No	No	Yes
147217-81-0	PBDE 37	No	No	Yes
243982-82-3	PBDE 49	No	No	Yes
5436-43-1	PBDE 47	No	No	Yes
93703-48-1	PBDE 77	No	No	Yes
189084-66-0	PBDE 119	No	No	Yes
189084-65-9	PBDE 116	No	No	Yes
65075-08-3	PBDE 31	No	No	Yes
446254-23-5	PBDE 50	No	No	Yes
106-48-9	4-Chlorophenol	No	No	Yes
13674-84-5	TCPP	No	No	Yes
1222-05-5	Galaxolide	No	No	Yes
78-70-6	linalool	No	No	Yes
95-16-9	benzothiazole	No	No	Yes
620-17-7	3-ethylphenol	Yes	No	No
108-68-9	3,5-dimethylphenol	Yes	No	Yes
99-89-8	4-isopropylphenol	Yes	No	Yes
91-59-8	2-naphthylamine	No	No	Yes
88-74-4	2-Nitroaniline	Yes	No	Yes

Table S1 (continued)

95-51-2	2-chloroaniline	Yes	No	Yes
95-82-9	2,5-dichloroaniline	No	No	Yes
106-49-0	p-toluidine	Yes	No	Yes
108-69-0	3,5-dimethylaniline	Yes	No	No
554-00-7	2,4-dichloroaniline	Yes	No	No
24544-04-5	2,6-diisopropylaniline	Yes	No	Yes
578-54-1	2-ethylaniline	Yes	No	Yes
91-64-5	coumarin	No	No	Yes
1806-26-4	4-n-octylphenol	Yes	No	Yes
1570-64-5	4-chloro-2-methylphenol	Yes	No	Yes
128-39-2	2,6-Di-tert-butylphenol	Yes	No	Yes
2416-94-6	2,3,6-trimethylphenol	Yes	No	Yes
150-19-6	3-methoxyphenol	Yes	No	Yes
1638-22-8	4-butylphenol	Yes	No	Yes
98-54-4	4-tert-butylphenol	Yes	No	Yes
120-95-6	2,4-di-tert-amylphenol	Yes	No	Yes
101-53-1	4-benzylphenol	Yes	No	Yes
82657-04-3	Bifenthrin	No	Yes	No
68085-85-8	cyhalothrin I	No	Yes	No
76703-62-3	cyhalothrin II	No	Yes	No
63466-71-7	benzo[a]pyrene-D12	No	Yes	No
6108-10-7	BHC epsilon	No	Yes	No
608-93-5	pentachlorobenzene	No	Yes	No

Table S2 GC-MS control parameters

GC-MS Settings		
Injection volume (µL)	1	
Mode	Pulsed Splitless	
Heater (°C)	265	
Pressure (psi)	RTL*	
Total flow (mL/min)	RTL*	
Septum purge flow (mL/min)	2	
System Carrier Gas	Helium	
Injection pulse pressure (psi)	25 until 0.5 min	
Purge flow to split vent (mL/min)	20 at 0.45 min	
Transfer line (°C)	300	
Column flow (mL/min)	RTL*	
Average velocity (cm/s)	RTL*	
**Temperature Program**		
**Time (min)**	**Ramp (°C/min)**	**Temp (°C)**
0	0	70
2	25	150
5.2	3	200
21.9	8	280
31.9	0	280
46.9	40	310
47.7	0	310
50.7	20	325
51.45	0	325
53.37	End Method	
**Mass Spec Settings**		
Source temp(°C)	300	
Quad temp (°C)	150	
**Scan Parameters**		
**Time (min)**	**Scan range (m/z)**	
0 – 29	50 - 500	
29 – 39	100 – 660	
39 - END	200 - 975	
**Column Settings & Specifications**		
Column Type	Agilent DB-5MS	
Length (m)	30	
Diameter (µm)	250	
Film thickness (µm)	0.25	

*RTL: retention time lock, pressure and associated parameters are adjusted based on instrument operating conditions.

Table S3 AMDIS Parameters.

AMDIS Parameters				
~Identification Tab~				
**Minimum Match Factor**	**Data Mode**	**RI Window**	**Match Factor Penalty**	**Max Penalty**
60	retention index	30 + 0	average	60
~Instrument Tab~				
**Use Scans Box**	**Thresholds**	**Scan Direction**	**Data File Format**	**Instrument Type**
checked	off	high to low	Agilent	quadrupole
~Deconvolution Tab~				
**Component Width**	**Omit m/z**	**Resolution**	**Adjacent Peak Subtraction**	**Sensitivity & Shape**
12	73, 207, 281	medium	2	medium

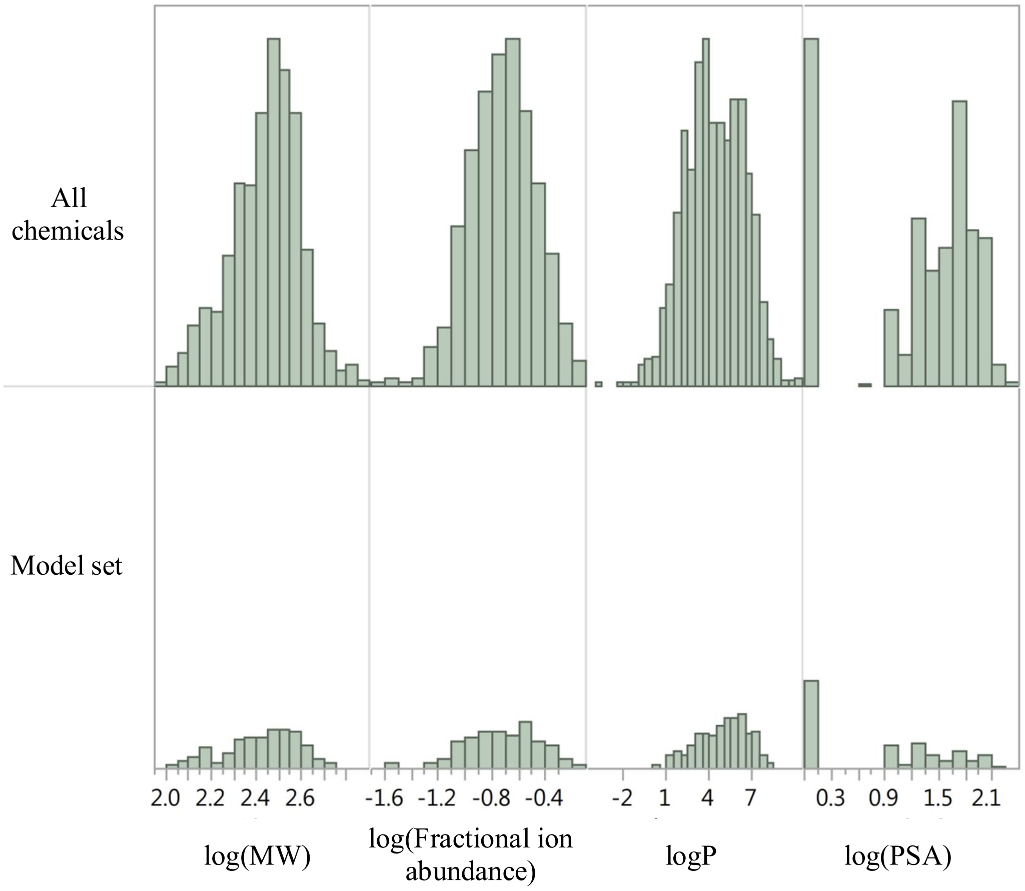

Fig. S1 Histograms of physico-chemical properties used as parameters for the complete list of target analytes and the set used to model response.

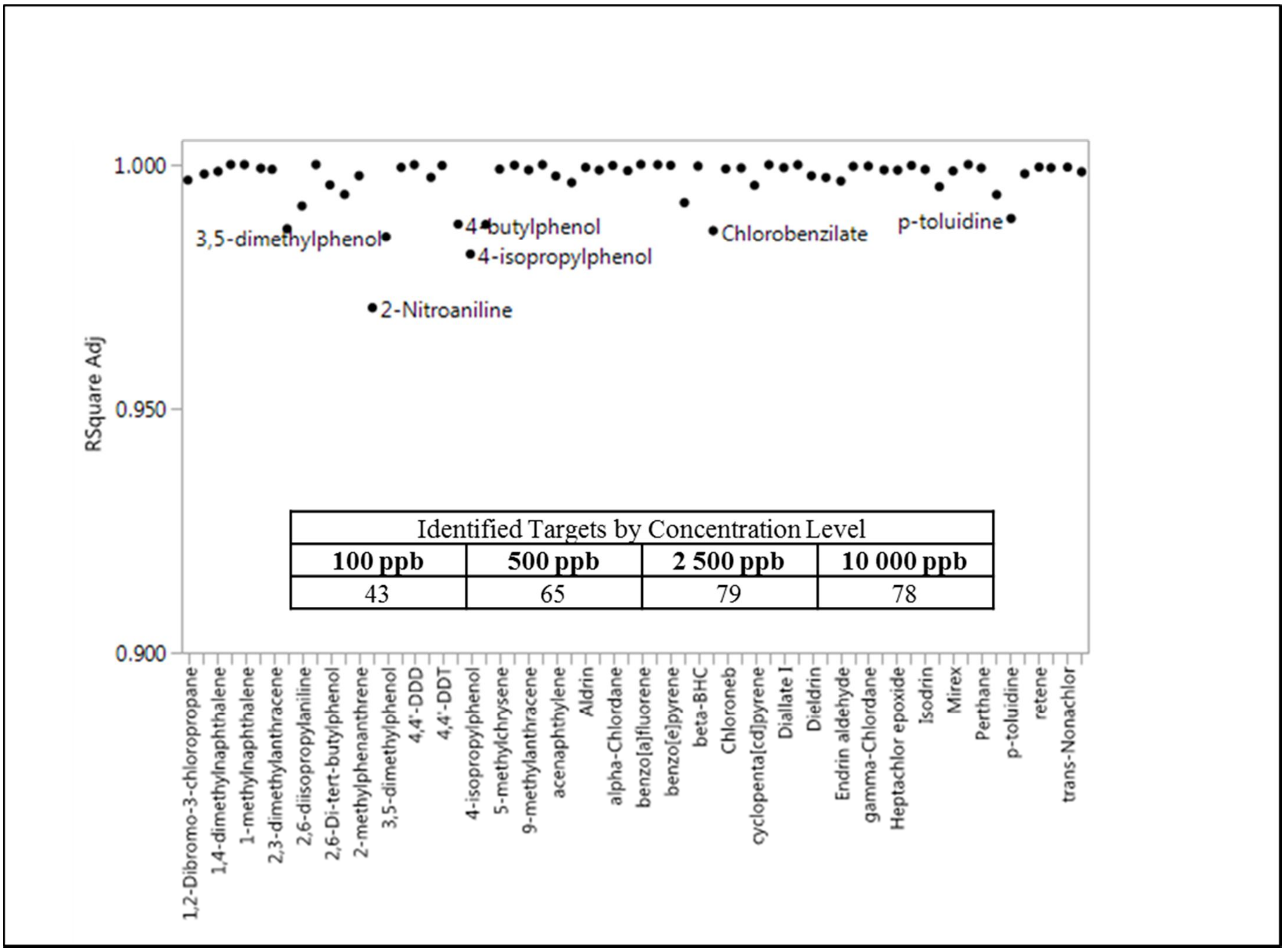

Identified Targets by Concentration Level			
**100 ppb**	**500 ppb**	**2 500 ppb**	**10 000 ppb**
43	65	79	78

Fig. S2 Goodness of fit for concentration series of subset of chemicals. RSquare Adj (R^2 adjusted) for 64 chemicals present in at least 3 of the 4 concentration levels evaluated on the MASV1500 method. The six chemicals that resulted in the lowest R^2 are labeled. Inset table shows the number of detected compounds per concentration level in this experiment

Table S4 Continuing calibration standard target concentrations after response factor applied

Name	*Target Response*	*Acceptable Response minimum (-30% Low)*	*Acceptable Response maximum (+30% High)*
1-naphthylamine	291	204	379
1,4-naphthalenedione	582	408	757
3-chlorophenol	337	236	439
4,4'-DDT	475	332	617
4,4'-DDD/DDE	NA	If detected, system may be dirty, perform maintenance if Sum DDD+DDE is > 20% of DDT+DDD+DDE.	
9-fluorenone	532	373	692
benzo[ghi]perylene	NA	Not evaluated in CV pass/fail	
benzothiazole	975	683	1268
chlorpyrifos	718	502	933
decachlorobiphenyl	516	362	671
endrin	612	429	796
galaxolide	198	138	257
linalool	319	223	415
musk ketone	313	219	407
naphthalene	585	410	761
pyrene	551	386	717
TCEP	282	197	367

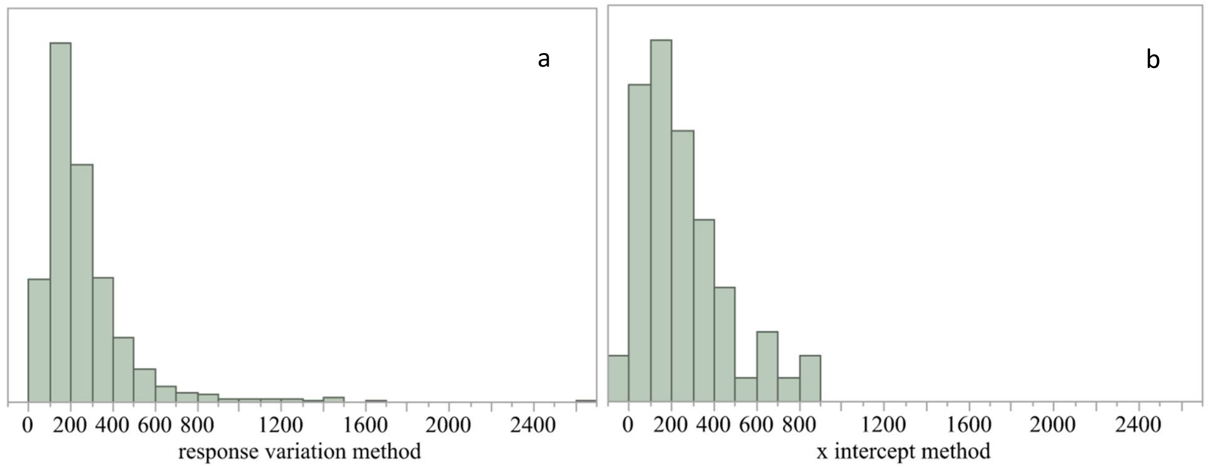

Fig. S3 Histogram of limits of quantitation (LOQs) determined by two different methods: (a) average response variation at 500 pg/µL applied to the modeled response factors (n=1550) and (b) linear extrapolation to the x-intercept (n = 64).

Table S5 List of all chemicals in method with model parameters & response factors at 500 pg/μL.

CASRN	Chemical	Fractional ion abundance	MW (g/mol)	logP	PSA (Å^2)	Response Factor at 500 pg/μL	Estimated LOQ (pg/μL)
2051-61-8	PCB 2	0.3004	188	4.54	0	3671135	51.49
2051-62-9	PCB 3	0.3025	188	4.55	0	3698125	51.11
13029-08-8	PCB 4	0.1965	222	4.93	0	2418475	78.16
16605-91-7	PCB 5	0.2163	222	4.89	0	2577525	73.34
25569-80-6	PCB 6	0.2227	222	5.02	0	2695755	70.12
33284-50-3	PCB 7	0.2239	222	5.04	0	2715710	69.61
34883-39-1	PCB 9	0.2232	222	4.95	0	2666580	70.89
2050-67-1	PCB 11	0.2276	222	5.11	0	2784120	67.89
2974-92-7	PCB 12	0.2258	222	4.99	0	2709020	69.78
2974-90-5	PCB 13	0.2304	222	5.11	0	2809215	67.29
34883-41-5	PCB 14	0.2272	222	5.12	0	2784890	67.88
2050-68-2	PCB 15	0.2340	222	5.12	0	2846805	66.4
38444-78-9	PCB 16	0.1411	256	5.37	0	1714390	110.26
37680-66-3	PCB 17	0.1389	256	5.51	0	1728775	109.34
37680-65-2	PCB 18	0.1382	256	5.43	0	1702520	111.03
38444-73-4	PCB 19	0.1370	256	5.42	0	1689385	111.89
38444-85-8	PCB 22	0.1751	256	5.47	0	2039910	92.66
55720-44-0	PCB 23	0.1694	256	5.4	0	1970700	95.92
55702-45-9	PCB 24	0.1622	256	5.31	0	1883655	100.35
55712-37-3	PCB 25	0.1763	256	5.6	0	2089000	90.49
38444-81-4	PCB 26	0.1771	256	5.51	0	2068850	91.37
38444-76-7	PCB 27	0.1641	256	5.51	0	1955815	96.65
7012-37-5	PCB 28	0.1789	256	5.61	0	2114645	89.39
15862-07-4	PCB 29	0.1762	256	5.41	0	2031840	93.03
35693-92-6	PCB 30	0.1669	256	5.54	0	1989190	95.03
16606-02-3	PCB 31	0.1797	256	5.52	0	2094355	90.26
38444-77-8	PCB 32	0.1706	256	5.51	0	2013005	93.9
38444-86-9	PCB 33	0.1765	256	5.47	0	2052410	92.1
37680-68-5	PCB 34	0.1720	256	5.6	0	2051425	92.14
37680-69-6	PCB 35	0.1827	256	5.55	0	2129785	88.75
38444-87-0	PCB 36	0.1822	256	5.69	0	2168165	87.18
53555-66-1	PCB 38	0.1763	256	5.44	0	2041700	92.58
38444-88-1	PCB 39	0.1828	256	5.69	0	2173730	86.96
38444-93-8	PCB 40	0.1066	290	5.81	0	1253120	150.85
36559-22-5	PCB 42	0.1166	290	5.95	0	1361010	138.89
41464-39-5	PCB 44	0.1098	290	5.87	0	1290030	146.53
70362-45-7	PCB 45	0.1083	290	5.79	0	1264965	149.43
41464-47-5	PCB 46	0.1047	290	5.79	0	1234565	153.11
2437-79-8	PCB 47	0.1359	290	6.1	0	1549875	121.96
70362-47-9	PCB 48	0.1251	290	5.88	0	1422400	132.89
41464-40-8	PCB 49	0.1266	290	6.01	0	1456050	129.82
68194-04-7	PCB 51	0.1192	290	6.01	0	1392320	135.76
35693-99-3	PCB 52	0.1220	290	5.92	0	1402105	134.82
41464-41-9	PCB 53	0.1154	290	5.92	0	1345730	140.46
15968-05-5	PCB 54	0.1432	290	5.91	0	1576825	119.88
70424-67-8	PCB 57	0.1612	290	5.97	0	1732835	109.09
41464-49-7	PCB 58	0.1621	290	6.04	0	1753535	107.8
74472-33-6	PCB 59	0.1588	290	5.88	0	1696095	111.45
33025-41-1	PCB 60	0.1687	290	5.92	0	1781780	106.09
33284-53-6	PCB 61	0.1612	290	5.73	0	1686555	112.08
54230-22-7	PCB 62	0.1587	290	5.78	0	1676595	112.74

Table S5 (continued)

CASRN	Chemical	Fractional ion abundance	MW (g/mol)	logP	PSA (Å^2)	Response Factor at 500 pg/µL	Estimated LOQ (pg/µL)
74472-34-7	PCB 63	0.1687	290	5.97	0	1791900	105.49
52663-58-8	PCB 64	0.1615	290	5.88	0	1717510	110.06
33284-54-7	PCB 65	0.1535	290	5.69	0	1619480	116.72
73575-53-8	PCB 67	0.1691	290	5.97	0	1795485	105.28
73575-52-7	PCB 68	0.1668	290	6.18	0	1820315	103.84
60233-24-1	PCB 69	0.1625	290	6.11	0	1771320	106.72
32598-11-1	PCB 70	0.1707	290	5.96	0	1805430	104.7
41464-46-4	PCB 71	0.1622	290	5.95	0	1736995	108.82
41464-42-0	PCB 72	0.1691	290	6.09	0	1819605	103.88
74338-23-1	PCB 73	0.1549	290	6.09	0	1705805	110.81
32690-93-0	PCB 74	0.1725	290	5.97	0	1821975	103.75
32598-12-2	PCB 75	0.1643	290	6.11	0	1785495	105.87
70362-48-0	PCB 76	0.1639	290	5.92	0	1744085	108.38
70362-49-1	PCB 78	0.1718	290	6.01	0	1824440	103.61
41464-48-6	PCB 79	0.1726	290	6.13	0	1856250	101.83
33284-52-5	PCB 80	0.1736	290	6.26	0	1891765	99.92
60145-20-2	PCB 83	0.0931	324	6.32	0	1019505	185.41
52663-60-2	PCB 84	0.0962	324	6.23	0	1036355	182.4
55312-69-1	PCB 86	0.1029	324	6.21	0	1087700	173.79
38380-02-8	PCB 87	0.1092	324	6.32	0	1147400	164.74
55215-17-3	PCB 88	0.1258	324	6.26	0	1267375	149.15
73575-57-2	PCB 89	0.1014	324	6.32	0	1086170	174.03
68194-05-8	PCB 91	0.1121	324	6.37	0	1174325	160.97
52663-61-3	PCB 92	0.1079	324	6.37	0	1141760	165.56
73575-56-1	PCB 93	0.1017	324	6.17	0	1074090	175.99
73575-55-0	PCB 94	0.1007	324	6.37	0	1085000	174.22
38379-99-6	PCB 95	0.1096	324	6.28	0	1146515	164.87
73575-54-9	PCB 96	0.1377	324	6.28	0	1356760	139.32
41464-51-1	PCB 97	0.1112	324	6.32	0	1162260	162.64
60233-25-2	PCB 98	0.1061	324	6.46	0	1136705	166.29
37680-73-2	PCB 101	0.1209	324	6.38	0	1243140	152.06
68194-06-9	PCB 102	0.1139	324	6.37	0	1188635	159.03
60145-21-3	PCB 103	0.1159	324	6.51	0	1218460	155.14
70424-68-9	PCB 107	0.1535	324	6.41	0	1487300	127.09
70362-41-3	PCB 108	0.1506	324	6.5	0	1477780	127.91
74472-35-8	PCB 109	0.1461	324	6.35	0	1425935	132.56
38380-03-9	PCB 110	0.1510	324	6.32	0	1457830	129.66
39635-32-0	PCB 111	0.1487	324	6.55	0	1470255	128.57
74472-36-9	PCB 112	0.1434	324	6.26	0	1395545	135.45
68194-10-5	PCB 113	0.1408	324	6.46	0	1400665	134.96
74472-38-1	PCB 115	0.1524	324	6.35	0	1471630	128.45
18259-05-7	PCB 116	0.1413	324	6.04	0	1354620	139.54
68194-11-6	PCB 117	0.0862	324	6.26	0	957820	197.35
31508-00-6	PCB 118	0.1591	324	6.42	0	1528495	123.67
56558-17-9	PCB 119	0.1523	324	6.55	0	1495995	126.36
68194-12-7	PCB 120	0.1557	324	6.55	0	1520890	124.29
56558-18-0	PCB 121	0.1258	324	6.68	0	1314010	143.86
76842-07-4	PCB 122	0.1373	324	6.36	0	1363220	138.66
65510-44-3	PCB 123	0.1504	324	6.5	0	1475805	128.08
70424-70-3	PCB 124	0.1501	324	6.41	0	1462475	129.25
74472-39-2	PCB 125	0.1422	324	6.41	0	1405345	134.51
39635-33-1	PCB 127	0.1576	324	6.58	0	1538295	122.88
55215-18-4	PCB 129	0.0866	358	6.65	0	850675	222.21

Table S5 (continued)

CASRN	Chemical	Fractional ion abundance	MW (g/mol)	logP	PSA (Å^2)	Response Factor at 500 pg/µL	Estimated LOQ (pg/µL)
52663-66-8	PCB 130	0.0873	358	6.78	0	862365	219.2
61798-70-7	PCB 131	0.0878	358	6.7	0	862140	219.25
38380-05-1	PCB 132	0.0900	358	6.69	0	877265	215.47
35694-04-3	PCB 133	0.0858	358	6.82	0	853395	221.5
52704-70-8	PCB 134	0.0839	358	6.61	0	828825	228.07
52744-13-5	PCB 135	0.0895	358	6.74	0	876470	215.67
35694-06-5	PCB 137	0.0932	358	6.79	0	905735	208.7
56030-56-9	PCB 139	0.1009	358	6.84	0	963790	196.13
59291-64-4	PCB 140	0.0968	358	6.92	0	938870	201.33
52712-04-6	PCB 141	0.0958	358	6.7	0	919745	205.52
41411-61-4	PCB 142	0.0876	358	6.52	0	851435	222.01
68194-15-0	PCB 143	0.0912	358	6.7	0	886250	213.29
68194-14-9	PCB 144	0.0983	358	6.75	0	939885	201.12
74472-40-5	PCB 145	0.1175	358	6.75	0	1072615	176.23
51908-16-8	PCB 146	0.1015	358	6.83	0	967025	195.47
68194-13-8	PCB 147	0.0963	358	6.75	0	926060	204.12
74472-41-6	PCB 148	0.0953	358	6.96	0	930950	203.05
38380-04-0	PCB 149	0.0997	358	6.74	0	949395	199.1
68194-08-1	PCB 150	0.1220	358	6.88	0	1111500	170.07
52663-63-5	PCB 151	0.0945	358	6.66	0	908110	208.15
68194-09-2	PCB 152	0.1170	358	6.66	0	1063095	177.81
60145-22-4	PCB 154	0.1065	358	6.97	0	1010625	187.04
33979-03-2	PCB 155	0.1270	358	7.1	0	1160840	162.84
69782-90-7	PCB 157	0.1307	358	6.82	0	1165405	162.2
74472-42-7	PCB 158	0.1296	358	6.79	0	1156005	163.52
41411-62-5	PCB 160	0.1233	358	6.7	0	1107865	170.62
74472-43-8	PCB 161	0.1212	358	6.93	0	1109525	170.37
39635-34-2	PCB 162	0.1301	358	6.86	0	1164115	162.38
74472-44-9	PCB 163	0.1259	358	6.7	0	1125030	168.02
74472-45-0	PCB 164	0.1245	358	6.78	0	1121205	168.59
74472-46-1	PCB 165	0.1055	358	6.84	0	995925	189.8
41411-63-6	PCB 166	0.1247	358	6.61	0	1110975	170.15
52663-72-6	PCB 167	0.1316	358	6.87	0	1174900	160.89
59291-65-5	PCB 168	0.1257	358	7	0	1145025	165.09
52663-71-5	PCB 171	0.0811	392	7.16	0	717685	263.38
52663-74-8	PCB 172	0.0766	392	7.16	0	687645	274.89
68194-16-1	PCB 173	0.0732	392	6.96	0	660060	286.38
40186-70-7	PCB 175	0.0792	392	7.21	0	706505	267.55
52663-65-7	PCB 176	0.0962	392	7.12	0	812750	232.58
52663-70-4	PCB 177	0.0756	392	7.07	0	678590	278.56
52663-67-9	PCB 178	0.0773	392	7.12	0	691545	273.34
74472-47-2	PCB 181	0.0831	392	7.1	0	729025	259.29
52663-69-1	PCB 183	0.0891	392	7.21	0	770850	245.22
74472-48-3	PCB 184	0.1059	392	7.35	0	880530	214.67
52712-05-7	PCB 185	0.0817	392	7.01	0	716965	263.65
74472-49-4	PCB 186	0.1027	392	7.01	0	849140	222.61
52663-68-0	PCB 187	0.0867	392	7.12	0	752425	251.22
74487-85-7	PCB 188	0.1028	392	7.26	0	858220	220.25
41411-64-7	PCB 190	0.1121	392	7.05	0	907455	208.31
74472-50-7	PCB 191	0.1081	392	7.25	0	890225	212.34
74472-51-8	PCB 192	0.1026	392	7.18	0	854170	221.3
69782-91-8	PCB 193	0.1067	392	7.18	0	879440	214.94
35694-08-7	PCB 194	0.0751	426	7.49	0	592635	318.96

Table S5 (continued)

CASRN	Chemical	Fractional ion abundance	MW (g/mol)	logP	PSA (Å^2)	Response Factor at 500 pg/µL	Estimated LOQ (pg/µL)
52663-78-2	PCB 195	0.0722	426	7.41	0	574765	328.88
42740-50-1	PCB 196	0.0768	426	7.54	0	603130	313.41
33091-17-7	PCB 197	0.0903	426	7.59	0	680295	277.86
68194-17-2	PCB 198	0.0694	426	7.46	0	558495	338.46
52663-75-9	PCB 199	0.0720	426	7.45	0	574065	329.28
40186-71-8	PCB 201	0.0900	426	7.5	0	677430	279.04
2136-99-4	PCB 202	0.0912	426	7.41	0	683115	276.71
52663-76-0	PCB 203	0.0808	426	7.47	0	625650	302.13
74472-52-9	PCB 204	0.0938	426	7.6	0	699950	270.06
74472-53-0	PCB 205	0.0967	426	7.5	0	714400	264.6
40186-72-9	PCB 206	0.0709	460	7.8	0	495675	381.35
52663-79-3	PCB 207	0.0903	460	7.84	0	593010	318.76
52663-77-1	PCB 208	0.0885	460	7.76	0	584110	323.62
111-46-6	Diethylene glycol	0.5045	106	-1.51	50	559365	337.93
62-53-3	Aniline	0.4794	93	0.94	26	1485760	127.23
106-46-7	p-Dichlorobenzene	0.3369	146	3.34	0	3709600	50.96
77-73-6	Dicyclopentadiene	0.5087	132	3.3	0	5358350	35.28
115-26-4	Dimefox	0.2715	154	-1.45	33	455155	415.31
95-50-1	o-Dichlorobenzene	0.3341	146	3.28	0	3624810	52.15
95-48-7	2-Methylphenol	0.2821	108	1.94	20	1461925	129.3
106-44-5	4-Methylphenol	0.3366	108	1.94	20	1665855	113.47
108-39-4	m-Cresol	0.2916	108	1.94	20	1498330	126.16
95-68-1	2,4-Dimethylaniline	0.2644	121	1.86	26	1241265	152.29
120-82-1	1,2,4-Trichlorobenzene	0.2581	180	3.82	0	2909055	64.98
2941-55-1	Ethiolate	0.3752	161	2.31	46	1469620	128.62
108-42-9	3-Chloroaniline	0.4211	127	1.81	26	1691505	111.75
106-47-8	4-Chloroaniline	0.4107	127	1.76	26	1634540	115.65
94-96-2	2-Ethyl-1,3-hexanediol	0.2489	146	1.25	40	864905	218.55
99-99-0	p-Nitrotoluene	0.2177	137	2.41	46	1096635	172.37
10265-92-6	Methamidophos	0.3063	141	-0.82	87	488005	387.35
62-73-7	Dichlorvos	0.4490	220	0.71	55	1043570	181.13
93-71-0	Allidochlor	0.1387	173	1.34	20	618770	305.49
933-78-8	2,3,5-Trichlorophenol	0.2191	196	3.69	20	1375035	137.47
1194-65-6	2,6-Dichlorobenzonitrile	0.3445	171	2.79	24	1678480	112.62
54-11-5	Nicotine	0.3401	162	0.72	16	1080735	174.91
759-94-4	EPTC	0.2463	189	3.37	46	1253505	150.8
37764-25-3	Dichlormid	0.2073	207	1.98	20	901760	209.62
122-59-8	Phenoxyacetic acid	0.2099	152	1.34	47	753150	250.98
92-52-4	Biphenyl	0.4049	154	3.98	0	4849320	38.98
24579-73-5	Propamocarb	0.5960	188	1.8	45	1718940	109.97
940-31-8	2-Phenoxypropionic acid	0.2305	166	1.69	47	861195	219.49
626-43-7	3,5-Dichloroaniline	0.3185	161	2.7	26	1584585	119.29
7786-34-7	Mevinphos	0.4589	224	0.28	81	918390	205.82
2008-41-5	Butylate	0.1694	217	4.06	46	972625	194.35
95-76-1	3,4-Dichloroaniline	0.3206	161	2.51	26	1516295	124.66
30560-19-1	Acephate	0.3291	183	-0.85	91	544685	347.04
24934-91-6	Chlormefos	0.1502	234	2.78	86	603060	313.45
1929-77-7	Vernolate	0.2706	203	3.9	46	1416020	133.49
131-11-3	Dimethyl phthalate	0.5711	194	1.64	53	1547885	122.12
122-42-9	Propham	0.1989	179	2.65	38	974635	193.95
1929-82-4	Nitrapyrin	0.2449	229	2.52	13	1150720	164.27
1114-71-2	Pebulate	0.2575	203	3.9	46	1365025	138.48
1129-41-5	Metolcarb	0.4060	165	1.63	38	1339225	141.15

Table S5 (continued)

CASRN	Chemical	Fractional ion abundance	MW (g/mol)	logP	PSA (Å^2)	Response Factor at 500 pg/µL	Estimated LOQ (pg/µL)
52-68-6	Trichlorfon	0.1187	256	0.48	66	360415	524.47
25013-16-5	Butylated hydroxyanisole	0.2653	180	3	29	1363510	138.63
62610-77-9	Methacrifos	0.1506	240	0.83	96	427400	442.27
90-43-7	o-Phenylphenol	0.3120	170	2.94	20	1673975	112.92
535-89-7	Crimidine	0.1412	171	1.73	29	650190	290.73
6597-78-0	Dicamba methyl ester	0.2183	234	3.09	36	970655	194.74
112-56-1	2-(2-Butoxyethoxy)ethyl thiocyanate	0.2534	203	1.68	68	807850	233.99
608-93-5	Pentachlorobenzene	0.2355	248	4.6	0	2317625	81.56
3547-33-9	2-(Octylthio)ethanol	0.1634	190	3.81	46	1014020	186.41
2212-67-1	Molinate	0.3858	187	3.21	46	1699530	111.22
2631-40-5	Isoprocarb	0.4124	193	2.51	38	1552490	121.76
682-80-4	Demephion	0.3613	216	1.06	95	865410	218.42
935-95-5	2,3,5,6-Tetrachlorophenol	0.2008	230	4.06	20	1201125	157.38
23844-56-6	Mecoprop methyl ester	0.1527	228	2.76	36	716470	263.83
96-45-7	Ethylenethiourea	0.6398	102	-0.66	56	880705	214.63
4901-51-3	2,3,4,5-Tetrachlorophenol	0.2051	230	4.39	20	1290725	146.45
16752-77-5	Methomyl	0.1589	162	0.6	76	459610	411.28
2436-73-9	MCPA methyl ester	0.1598	214	2.56	36	741825	254.81
107-49-3	Tetraethyl pyrophosphate	0.1505	290	1.03	100	423295	446.56
23560-59-0	Heptenophos	0.2216	250	1.77	55	716175	263.94
134-62-3	N,N-Diethyl-m-toluamide	0.3923	191	1.96	20	1487450	127.08
14437-17-3	Chlorfenprop-methyl	0.1407	232	2.88	26	719310	262.79
1113-02-6	Omethoate	0.2676	213	-0.74	100	490130	385.67
84-66-2	Diethyl phthalate	0.4617	222	2.7	53	1529180	123.61
117-18-0	Tecnazene	0.0843	259	3.73	46	470320	401.91
297-97-2	Thionazin	0.0960	248	1.58	95	342490	551.92
534-52-1	4,6-Dinitro-o-cresol (DNOC)	0.2316	198	2.19	112	777975	242.97
57153-17-0	Dichlorprop methyl ester	0.1895	248	3	36	828630	228.12
919-86-8	Demeton-S-methyl	0.3097	230	1.32	96	796290	237.39
122-39-4	Diphenylamine	0.3401	169	2.97	12	1960600	96.41
103-33-3	Azobenzene	0.3781	182	3.82	25	2173415	86.97
119-61-9	Benzophenone	0.3446	182	3.18	17	1869370	101.12
1134-23-2	Cycloate	0.3035	215	3.83	46	1445915	130.73
1928-38-7	2,4-D methyl ester	0.1322	234	2.65	36	622555	303.63
101-42-8	Fenuron	0.3447	164	1.45	36	1146355	164.89
1918-00-9	Dicamba	0.0849	220	2.76	47	453540	416.78
101-21-3	Chlorpropham	0.1710	213	3.49	38	923925	204.59
5825-87-6	2-(3-Chlorophenoxy)propionamide	0.2602	199	1.93	52	914020	206.81
6164-98-3	Chlordimeform	0.1280	196	3.01	16	831195	227.42
2655-15-4	2,3,5-Trimethacarb	0.3055	193	2.55	38	1254465	150.68
55283-68-6	Ethalfluralin	0.0698	333	5.1	95	325760	580.27
141-66-2	Dicrotophos	0.4866	237	-0.36	75	875690	215.86
39196-18-4	Thiofanox	0.1279	218	2.37	76	529845	356.76
1689-84-5	Bromoxynil	0.2722	275	2.95	44	971000	194.67
22781-23-3	Bendiocarb	0.3020	223	1.86	57	947965	199.4
1861-40-1	Benfluralin	0.3305	335	5.41	95	1046445	180.64
6923-22-4	Monocrotophos	0.4643	223	-0.45	84	808410	233.83
3689-24-5	Sulfotep	0.1559	322	3.99	130	528050	357.97
35256-85-0	Tebutam	0.3899	233	3.14	20	1662225	113.72
999055-03-7	Desbromo-bromobutide	0.1790	233	3.2	29	887625	212.96
2631-37-0	Promecarb	0.3531	207	2.96	38	1455395	129.88
298-02-2	Phorate	0.2481	260	3.67	111	888425	212.77
999057-03-3	Triclopyr methyl ester	0.1400	269	3.05	48	601710	314.15

Table S5 (continued)

CASRN	Chemical	Fractional ion abundance	MW (g/mol)	logP	PSA (Å^2)	Response Factor at 500 pg/µL	Estimated LOQ (pg/µL)
2876-78-0	Methyl-1-naphthalene acetate	0.4604	200	3.2	26	2027420	93.24
640-15-3	Thiometon	0.2914	246	2.25	111	842190	224.45
13684-56-5	Desmedipham	0.1941	300	4.49	84	752150	251.32
533-74-4	Dazomet	0.1760	162	0.16	64	456060	414.48
99-30-9	Dichloran	0.1204	206	3.54	72	663015	285.1
1825-21-4	Pentachloroanisole	0.0990	278	4.97	9	760215	248.65
126-75-0	Demeton-S	0.2834	258	2.38	96	842055	224.48
502-55-6	Diethyl dithiobis(thionoformate) (EXD)	0.4013	242	4.3	133	1440720	131.2
1610-17-9	Atraton	0.1525	211	0.98	70	476800	396.45
120-36-5	Dichlorprop	0.2520	234	2.93	47	1004640	188.15
2686-99-9	3,4,5-Trimethacarb	0.3154	193	2.55	38	1284175	147.2
91-53-2	Ethoxyquin	0.3936	217	3.93	21	2019135	93.62
1610-18-0	Prometon	0.1459	225	1.33	70	485300	389.51
1563-66-2	Carbofuran	0.2312	221	2.72	51	928805	203.52
1918-18-9	Swep	0.1902	219	3.41	38	963760	196.13
1967-16-4	Chlorbufam	0.1918	223	3.17	38	916440	206.26
55290-64-7	Dimethipin	0.2100	210	-0.35	85	452455	417.78
81777-89-1	Clomazone	0.3231	239	2.17	30	1136620	166.31
4841-20-7	Fenoprop methyl ester	0.1441	282	3.63	36	670175	282.06
139-40-2	Propazine	0.1534	229	1.88	61	564050	335.13
66215-27-8	Cyromazine	0.2402	166	-0.04	103	503605	375.35
58-89-9	Lindane	0.1004	288	3.99	0	982225	192.45
33693-04-8	Terbumeton	0.2288	225	0.7	70	605915	311.97
7286-84-2	Chloramben methyl ester	0.1666	219	2.54	52	706985	267.37
999056-03-0	N-Methyl-N-1-naphthyl acetamide	0.2085	199	2.77	29	1017990	185.69
30979-48-7	Isocarbamide	0.4344	185	-0.72	61	739525	255.61
2032-59-9	Aminocarb	0.2483	208	2.01	42	913505	206.93
2163-69-1	Cycluron	0.2794	198	2.98	32	1302685	145.11
131-16-8	Di-n-propyl phthalate	0.6390	250	3.76	53	2124365	88.98
2636-26-2	Cyanophos	0.2294	243	2.71	93	786370	240.38
13071-79-9	Terbufos	0.2052	288	4.37	111	768775	245.88
57369-32-1	Pyroquilon	0.2432	173	1.4	20	950790	198.81
5915-41-3	Terbuthylazine	0.2227	229	1.26	61	668175	282.9
1912-26-1	Trietazine	0.1756	229	3.34	54	817365	231.26
31218-83-4	Propetamphos	0.1971	281	1.61	99	558420	338.5
23950-58-5	Propyzamide	0.1915	255	4.35	33	1011300	186.91
1928-37-6	2,4,5-T methyl ester	0.1235	268	3.28	36	594955	317.72
57153-18-1	MCPB methyl ester	0.2965	242	3.54	36	1276655	148.06
26399-36-0	Profluralin	0.2987	347	5.25	95	918790	205.74
1420-07-1	Dinoterb	0.2922	240	3.42	112	1028125	183.86
53112-28-0	Pyrimethanil	0.5131	199	2.84	38	1920630	98.42
83-26-1	Pindone	0.3254	230	2.12	51	1054410	179.27
117-80-6	Dichlone	0.1332	226	2.35	34	612175	308.78
13171-21-6	Phosphamidon	0.2594	299	0.86	75	649565	291.01
298-04-4	Disulfoton	0.2362	274	3.31	111	783505	241.26
88-85-7	Dinoseb	0.2285	240	3.61	112	883655	213.92
950-35-6	Methyl paraoxon	0.2030	247	1.25	100	562215	336.22
24691-80-3	Fenfuram	0.5528	201	2.24	42	1755135	107.7
33245-39-5	Fluchloralin	0.1456	355	4.64	95	505280	374.1
999052-03-8	2,4-D sec-butyl ester	0.1286	276	2.59	47	526075	359.32
26259-45-0	Secbumeton	0.3145	225	3.09	72	1160950	162.82
5902-51-2	Terbacil	0.2141	216	1.76	49	749560	252.18
29091-05-2	Dinitramine	0.1009	322	5.86	121	456420	414.15

Table S5 (continued)

CASRN	Chemical	Fractional ion abundance	MW (g/mol)	logP	PSA (Å^2)	Response Factor at 500 pg/µL	Estimated LOQ (pg/µL)
2303-17-5	Triallate	0.2213	303	6.18	46	1081085	174.85
42509-80-8	Isazophos	0.0964	313	2.99	100	361515	522.88
16709-30-1	Carbofuran-3-keto	0.2011	235	1.44	65	627500	301.24
38260-54-7	Etrimfos	0.1289	292	3.67	105	502010	376.54
1715-40-8	Bromocyclen	0.0641	390	5.13	0	559960	337.57
115-31-1	Isobornyl thiocyanoacetate	0.1212	253	3.96	75	597320	316.46
7286-69-3	Sebuthylazine	0.3175	229	3.16	63	1195905	158.06
74782-23-3	Oxabetrinil	0.5721	232	1.51	64	1383980	136.58
3369-52-6	Endosulfan ether	0.0904	340	3.95	9	523085	361.37
26087-47-8	Iprobenfos	0.2828	288	3.21	71	920565	205.34
7287-36-7	Monalide	0.2990	239	4.34	29	1530460	123.51
3060-89-7	Metobromuron	0.1509	258	2.32	42	603180	313.39
527-20-8	Pentachloroaniline	0.2192	263	4.86	26	1209540	156.28
2540-82-1	Formothion	0.1759	257	1.43	123	496200	380.95
18625-12-2	2,4-DB methyl ester	0.2929	262	3.64	36	1204120	156.98
29973-13-5	Ethiofencarb	0.4153	225	2.04	64	1209780	156.25
60568-05-0	Furmecyclox	0.6207	251	3.58	43	2090335	90.43
23103-98-2	Pirimicarb	0.3934	238	1.7	59	1088825	173.61
6099-79-2	Dinoseb methyl ether	0.2234	254	3.43	101	822570	229.8
34681-23-7	Butoxycarboxim	0.1133	222	-0.72	93	266675	708.84
575-36-0	N-1-Naphthylacetamide	0.4441	185	2.31	29	1679185	112.57
6988-21-2	Dioxacarb	0.2171	239	0.57	57	587535	321.73
68505-69-1	Benfuresate	0.3779	256	2.82	61	1204170	156.98
1014-69-3	Desmetryn	0.2130	213	2.56	88	790165	239.22
1918-13-4	Chlorthiamid	0.2097	205	2.23	63	791095	238.94
97-17-6	Dichlofenthion	0.1443	314	5.12	70	630715	299.7
50563-36-5	Dimethachlor	0.3153	255	2.13	30	1077015	175.51
22936-86-3	Cyprazine	0.1823	227	2.47	63	708280	266.88
999047-03-9	Phosphamidon II	0.2641	299	0.86	75	658300	287.15
74712-19-9	Bromobutide	0.2220	311	3.94	29	909210	207.9
21087-64-9	Metribuzin	0.2819	214	1.3	96	753525	250.86
2275-18-5	Prothoate	0.1435	285	2.42	115	471240	401.13
61592-45-8	Bentazone methyl derivative	0.2434	254	0.64	66	627520	301.23
34256-82-1	Acetochlor	0.0814	269	2.92	30	428880	440.74
3878-19-1	Fuberidazole	0.4045	184	2.67	42	1601300	118.05
21757-82-4	Plifenat	0.3007	334	4.73	26	1156575	163.44
1918-11-2	Terbucarb	0.3638	277	5	38	1584715	119.28
2164-09-2	Chloranocryl	0.3210	229	3.93	29	1568750	120.5
16655-82-6	3-Hydroxycarbofuran	0.2416	237	0.21	68	582300	324.62
63-25-2	Carbaryl	0.3872	201	3.35	42	1690505	111.82
57018-04-9	Tolclofos-methyl	0.3761	300	4.03	70	1205835	156.76
1014-70-6	Simetryn	0.2491	213	1.21	86	689740	274.05
93-72-1	Fenoprop	0.1762	282	3.56	47	737020	256.47
1634-78-2	Malathion-o-analog	0.2274	314	2.07	123	602295	313.85
34123-59-6	Isoproturon	0.2629	206	2.32	32	1064940	177.5
834-12-8	Ametryne	0.1935	227	1.56	86	599570	315.27
1216-44-0	Flurenol-methylester	0.4327	240	1.99	47	1268220	149.05
644-64-4	Dimetilan	0.7849	240	-0.49	68	1244430	151.9
58138-08-2	Tridiphane	0.0759	318	4.3	13	475155	397.82
299-84-3	Fenchlorphos	0.3006	320	4.84	70	1034960	182.64
301-12-2	Oxydemeton-methyl	0.1542	246	-0.74	107	334265	565.5
57837-19-1	Metalaxyl	0.1031	279	2.15	56	408245	463.03
311-45-5	Paraoxon	0.1335	275	2.31	100	459910	411.01

Table S5 (continued)

CASRN	Chemical	Fractional ion abundance	MW (g/mol)	logP	PSA (Å^2)	Response Factor at 500 pg/µL	Estimated LOQ (pg/µL)
7287-19-6	Prometryn	0.1777	241	1.91	86	584120	323.61
2813-95-8	Dinoseb acetate	0.4531	282	3.29	118	1225585	154.23
86-86-2	2-(1-naphthyl)acetamide	0.3328	185	1.68	43	1102695	171.42
17040-19-6	Demeton-S-methylsulfon	0.2831	262	-0.11	113	575300	328.57
64529-56-2	Tycor (SMY 1500)	0.1418	228	1.83	96	489085	386.49
14143-55-6	Picloram methyl ester	0.1648	254	3.07	65	672240	281.19
1689-83-4	Ioxynil	0.4279	371	3.6	44	1154560	163.72
3204-27-1	Dinoterb acetate	0.0730	282	3.11	118	311060	607.69
886-50-0	Terbutryn	0.1666	241	1.28	86	503955	375.09
2032-65-7	Methiocarb	0.3007	225	3.55	67	1232630	153.35
97886-45-8	Dithiopyr	0.1707	401	5.88	98	517470	365.29
2797-51-5	Quinoclamine	0.2109	207	1.31	60	665685	283.96
330-55-2	Linuron	0.1679	248	3.33	45	767040	246.44
2307-68-8	Pentanochlor	0.3125	239	4.21	29	1548365	122.08
85785-20-2	Esprocarb	0.2883	265	4.16	46	1213600	155.76
29232-93-7	Pirimiphos-methyl	0.1627	305	4	99	600590	314.74
26225-79-6	Ethofumesate	0.2237	286	2	70	677160	279.15
27605-76-1	Probenazole	0.0888	223	1.87	64	376670	501.84
314-40-9	Bromacil	0.2269	380	2.1	49	648315	291.57
15545-48-9	Chlorotoluron	0.1881	212	2.62	36	850860	222.16
1085-98-9	Dichlofluanid	0.2061	332	3.45	74	666095	283.78
84-74-2	Di-n-butyl phthalate	0.6752	278	4.82	53	2304540	82.02
919-76-6	Amidithion	0.1359	273	0.64	124	363790	519.61
28249-77-6	Benthiocarb	0.3716	257	3.53	46	1377655	137.21
4147-51-7	Dipropetryn	0.1761	255	3.97	88	761720	248.16
23135-22-0	Oxamyl	0.1185	219	-0.47	96	285935	661.09
121-75-5	Malathion	0.1482	330	2.92	138	451230	418.91
42588-37-4	Kinoprene	0.1194	276	6.68	26	918870	205.72
55-38-9	Fenthion	0.3300	278	3.21	95	1006960	187.72
87130-20-9	Diethofencarb	0.1674	267	2.92	57	658630	287
67628-93-7	Dimethylvinphos(Z)	0.2459	330	3.45	55	802070	235.67
3244-90-4	Tetrapropyl thiodiphosphate	0.2223	378	6.11	130	657155	287.64
67564-91-4	Fepropimorph	0.6596	303	5.2	12	2738120	69.04
57052-04-7	Isomethiozin	0.1664	268	1.32	83	496270	380.89
21725-46-2	Cyanazine	0.1632	240	0.2	85	418760	451.4
43121-43-3	Triadimefon	0.2024	293	2.77	57	702050	269.25
2463-84-5	Dicapthon	0.4320	297	3.46	115	1164860	162.27
297-78-9	Isobenzan	0.0629	408	4.5	9	344845	548.15
2873-17-8	Methfuroxam	0.5233	229	3.16	42	1853760	101.97
16118-49-3	Carbetamide	0.3142	236	2.83	74	1070805	176.53
36756-79-3	Tiocarbazil I	0.2067	279	4.69	46	964620	195.96
80-38-6	Fenson	0.2868	268	3.89	52	1130245	167.24
999051-03-5	Tiocarbazil II	0.2104	279	4.69	46	977195	193.44
25057-89-0	Bentazone	0.2140	240	2.8	75	792015	238.67
500-28-7	Chlorthion	0.1621	297	3.55	115	569760	331.77
27355-22-2	Phthalide	0.1814	270	2.73	26	772640	244.65
327-98-0	Trichloronate	0.1540	332	4.49	60	604675	312.61
10552-74-6	Nitrothal-isopropyl	0.1572	295	3.72	98	586370	322.37
5707-69-7	Drazoxolon	0.1748	237	2.57	63	682195	277.09
299-86-5	Crufomate	0.1806	291	3.33	57	690275	273.84
1593-77-7	Dodemorph I	0.3664	281	6.1	12	2165305	87.3
61213-25-0	Flurochloridone I	0.1149	311	2.47	20	516120	366.25
10544-50-0	Sulfur (S8)	0.3829	256	3.73	202	1128635	167.48

Table S5 (continued)

CASRN	Chemical	Fractional ion abundance	MW (g/mol)	logP	PSA (Å^2)	Response Factor at 500 pg/µL	Estimated LOQ (pg/µL)
24691-76-7	Pyracarbolid	0.4938	217	2.52	38	1667100	113.39
2104-96-3	Bromophos	0.2531	364	5.07	70	799700	236.37
999043-03-7	Flurochloridone II	0.1195	311	2.47	20	531175	355.86
81-84-5	Naphthalic anhydride	0.2545	198	1.75	43	896640	210.82
957-51-7	Diphenamid	0.2909	239	2.63	20	1211400	156.04
33629-47-9	Butralin	0.2344	295	5.8	104	978730	193.14
3868-61-9	Endosulfan lactone	0.0539	354	3.73	26	285460	662.18
29082-74-4	Octachlorostyrene	0.0738	376	5.57	0	663625	284.84
23505-41-1	Pirimiphos-ethyl	0.0999	333	5.06	99	420380	449.66
33820-53-0	Isopropalin	0.2904	309	5.3	95	1041690	181.46
27304-13-8	Oxychlordane	0.0546	420	4.23	13	282765	668.49
999040-03-8	Dodemorph II	0.3843	281	6.1	12	2242950	84.28
67129-08-2	Metazachlor	0.1588	277	2.11	38	598955	315.6
40596-69-8	Methoprene I	0.2614	310	5.63	36	1168975	161.7
53780-34-0	Mefluidide	0.3420	310	3.46	84	1000425	188.95
148-79-8	Thiabendazole	0.3483	201	2.47	70	1198480	157.72
25059-80-7	Benazolin-ethyl	0.2069	271	3.21	72	761455	248.24
101-05-3	Anilazine	0.2558	274	3.07	51	920680	205.31
13360-45-7	Chlorbromuron	0.1447	292	3.26	42	610780	309.48
66246-88-6	Penconazole	0.1974	283	3.66	31	867680	217.85
22936-75-0	Dimethametryn	0.4890	255	3.97	88	1620545	116.64
150-50-5	Merphos	0.2120	298	8.7	89	1254215	150.71
947-02-4	Phosfolan	0.1240	255	0.99	108	368380	513.13
133-06-2	Captan	0.2472	299	1.85	63	716970	263.65
731-27-1	Tolylfluanid	0.1660	346	3.91	74	567535	333.07
88283-41-4	Pyrifenox I	0.2595	294	3.42	34	987025	191.51
42609-73-4	Methyldymron	0.2585	268	3.01	32	1011095	186.95
330-54-1	Diuron	0.2237	232	2.88	36	959460	197.01
950-10-7	Mephosfolan	0.1333	269	1.48	108	410885	460.05
84332-86-5	Chlozolinate	0.0974	331	3.11	73	374040	505.37
470-90-6	Chlorfenvinphos	0.1573	358	4.51	55	577560	327.29
133-07-3	Folpet	0.1100	295	2.85	63	441635	428.02
25311-71-1	Isofenphos	0.1473	345	4.12	99	503135	375.7
1086-02-8	Pyridinitril	0.1820	273	2.64	60	660570	286.16
13593-03-8	Quinalphos	0.1649	298	3.53	95	593600	318.44
55219-65-3	Triadimenol	0.2023	295	3.04	60	713470	264.94
2597-03-7	Phenthoate	0.1791	320	3.96	112	602115	313.94
2595-54-2	Mecarbam	0.1034	329	2.31	132	332080	569.22
28434-00-6	Bioallethrin S-cyclopentenyl isomer	0.3062	302	4.92	43	1226135	154.17
103-17-3	Chlorbenside	0.4448	268	5.42	25	2166885	87.23
584-79-2	Bioallethrin	0.3031	302	4.92	43	1217055	155.32
2439-01-2	Chinomethionat	0.1839	234	1.99	93	605970	311.94
57646-30-7	Furalaxyl	0.3429	301	2.52	60	985050	191.9
32809-16-8	Procymidone	0.1907	283	2.67	37	729550	259.1
973-21-7	Dinobuton	0.1874	326	4.26	127	614655	307.53
2536-31-4	Chlorflurecol-methyl ester	0.3565	274	2.58	47	1120985	168.63
2314-09-2	Flurenol-butyl ester	0.5126	282	3.58	47	1626775	116.2
7700-17-6	Crotoxyphos	0.2443	314	2.1	81	684105	276.31
950-37-8	Methidathion	0.3872	302	2.03	153	870925	217.04
999045-03-3	Methoprene II	0.1466	310	5.63	36	762155	248.02
68694-11-1	Triflumizole	0.1496	345	4.66	39	619575	305.09
3424-82-6	2,4'-DDE	0.1984	316	6.22	0	1834160	103.06
4824-78-6	Bromophos-ethyl	0.0999	392	6.13	70	384200	492

Table S5 (continued)

CASRN	Chemical	Fractional ion abundance	MW (g/mol)	logP	PSA (Å^2)	Response Factor at 500 pg/µL	Estimated LOQ (pg/µL)
76738-62-0	Paclobutrazol	0.2337	293	2.99	51	815355	231.83
999049-03-5	Pyrifenox II	0.2037	294	3.42	34	825080	229.1
2275-23-2	Vamidothion	0.2610	287	0.15	125	553825	341.31
5103-71-9	alpha-Chlordane	0.1018	406	5.57	0	762080	248.04
961-11-5	Tetrachlorvinphos	0.2083	364	3.86	55	673135	280.82
97-16-5	2,4-Dichlorophenyl benzenesulfonate	0.2640	302	4.46	52	1014610	186.31
21564-17-0	TCMTB	0.2260	238	3.12	116	810360	233.26
76674-21-0	Flutriafol	0.2341	301	2.31	51	747065	253.03
5131-24-8	Ditalimfos	0.2026	299	3.48	98	682050	277.15
23184-66-9	Butachlor	0.1213	311	4.51	30	610740	309.5
80-33-1	Chlorfenson	0.2329	302	4.75	52	953090	198.33
62924-70-3	Flumetralin	0.3307	421	5.91	95	793955	238.08
15299-99-7	Napropamide	0.2485	271	3.24	30	1015195	186.2
131-18-0	Diamyl phthalate	0.6410	306	5.89	53	2218345	85.21
79983-71-4	Hexaconazole	0.1707	313	3.66	51	659005	286.84
18181-70-9	Jodfenphos	0.3411	412	5.51	70	872310	216.7
36335-67-8	Butamifos	0.1261	332	4.62	118	469665	402.47
41814-78-2	Tricyclazole	0.2625	189	2.28	58	995350	189.91
22224-92-6	Fenamiphos	0.1690	303	3.68	83	619695	305.03
38727-55-8	Diethatyl ethyl	0.1367	311	3.58	47	565810	334.08
15457-05-3	Fluorodifen	0.2503	328	3.65	101	748245	252.63
34643-46-4	Prothiofos	0.0968	344	5.53	86	418970	451.17
35554-44-0	Imazalil	0.1899	296	3.58	27	824140	229.36
66332-96-5	Flutolanil	0.5028	323	5.38	42	1712460	110.38
80-05-7	Bisphenol A	0.4170	228	3.43	40	1660775	113.82
50512-35-1	Isoprothiolane	0.1227	290	3.22	103	464060	407.33
41198-08-7	Profenofos	0.0798	372	4.6	71	323395	584.51
83657-17-4	Uniconizole-P	0.2729	291	3.84	51	1010755	187.02
101-27-9	Barban	0.2138	257	3.87	38	992550	190.45
78-48-8	S,S,S-Tributylphosphorotrithioate	0.1155	314	3.23	103	419530	450.57
51218-49-6	Pretilachlor	0.1407	311	4.25	30	664920	284.29
5234-68-4	Carboxin	0.3522	235	3	64	1231750	153.46
53-19-0	2,4'-DDD	0.2556	318	5.39	0	2032515	93
37893-02-0	Flubenzimine	0.1667	416	6.78	66	528955	357.36
75736-33-3	Diclobutrazol	0.1965	327	3.6	51	701885	269.31
88671-89-0	Myclobutanil	0.1826	288	2.82	55	665260	284.14
41394-05-2	Metamitron	0.2114	202	0.83	71	591725	319.45
60207-31-0	Azaconazole	0.2463	299	2.32	49	784415	240.98
52756-25-9	Flamprop-methyl	0.5131	335	3.93	47	1455615	129.86
69327-76-0	Buprofezin	0.1153	305	4.29	61	520970	362.84
85509-19-9	Flusilazole	0.3827	315	3.84	31	1317265	143.5
841-06-5	Methoprotryne	0.1683	271	2.37	97	556890	339.43
64470-88-8	Tryclopyrbutoxyethyl	0.1338	355	4.12	58	499815	378.19
1836-75-5	Nitrofen	0.1505	283	4.92	55	748960	252.39
136-25-4	Erbon	0.1559	364	4.65	36	610730	309.51
18854-01-8	Isoxathion	0.2212	313	3.68	96	718110	263.23
12771-68-5	Ancymidol	0.2569	256	0.82	55	690770	273.65
485-31-4	Binapacryl	0.6596	322	4.9	118	1689280	111.9
79241-46-6	Fluazifop-p-butyl	0.2511	383	3.64	58	725260	260.63
50594-67-7	Acifluorfen methyl ester	0.1103	375	4.32	81	392585	481.49
5836-10-2	Chloropropylate	0.2651	338	5.08	47	966880	195.5
115-90-2	Fensulfothion	0.1541	308	2.23	106	474755	398.16
3761-41-9	Fenthion sulfoxide	0.2025	294	1.63	106	556070	339.93

Table S5 (continued)

CASRN	Chemical	Fractional ion abundance	MW (g/mol)	logP	PSA (Å^2)	Response Factor at 500 pg/µL	Estimated LOQ (pg/µL)
83657-24-3	Diniconazole	0.2067	325	4.23	51	772575	244.67
69581-33-5	Cyprofuram	0.2789	279	0.69	47	733860	257.58
2635-10-1	Methiocarb sulfoxide	0.1976	241	0.62	75	526985	358.69
60207-93-4	Etaconazole	0.1495	327	3.35	49	565390	334.33
789-02-6	2,4'-DDT	0.2411	352	5.92	0	1774780	106.51
52756-22-6	Flamprop-isopropyl	0.5420	363	4.81	47	1484860	127.3
77732-09-3	Oxadixyl	0.1079	278	0.68	59	349660	540.61
2179-25-1	Methiocarb sulfone	0.1702	257	1.26	81	507675	372.34
1031-47-6	Triamiphos	0.4026	294	-0.71	90	734940	257.2
15310-01-7	Benodanil	0.3374	323	3.23	29	1126075	167.86
143-50-0	Kepone	0.1293	486	5.89	17	434735	434.81
2227-13-6	Tetrasul	0.1445	320	7.27	25	889650	212.47
60238-56-4	Chlorthiophos	0.1106	360	5.45	95	426475	443.23
14255-88-0	Fenazaflor	0.1417	374	5.59	44	560620	337.17
55814-41-0	Mepronil	0.5043	269	4.87	42	2017270	93.7
35400-43-2	Sulprofos	0.1583	322	4.64	111	580855	325.43
24017-47-8	Triazophos	0.1938	313	3.99	100	665830	283.9
1836-77-7	Chlornitrofen	0.0876	317	5.33	55	458115	412.62
51207-31-9	2,3,7,8-Tetrachlorodibenzofuran	0.2338	304	6.45	13	1420275	133.09
786-19-6	Carbophenothion	0.1537	342	5.61	111	571915	330.51
52-85-7	Famphur	0.3704	325	2.13	115	865280	218.46
71626-11-4	Benalaxyl	0.2682	325	3.88	47	921835	205.06
17109-49-8	Edifenphos	0.2128	310	3.48	87	701525	269.45
13067-93-1	Cyanofenphos	0.2507	303	4.29	84	881340	214.48
1689-99-2	Bromoxynil octanoic acid ester	0.3490	401	5.19	50	962430	196.41
2164-08-1	Lenacil	0.6278	234	1.98	53	1650665	114.52
60207-90-1	Tilt	0.1483	341	3.88	49	566110	333.91
56-53-1	Diethylstilbestrol	0.2092	268	5.93	40	1226800	154.08
27314-13-2	Norflurazon	0.1844	303	2.45	45	646740	292.28
84-16-2	Hexestrol	0.4746	270	4.98	40	1964760	96.21
85-68-7	Butyl benzyl phthalate	0.2793	312	5	53	1073710	176.05
1698-60-8	Pyrazon	0.2001	221	0.73	59	569145	332.12
1746-01-6	2,3,7,8-TCDD	0.2054	320	7.01	18	1190710	158.75
999048-03-2	Propiconazole-II	0.1484	341	3.88	49	566420	333.72
3478-94-2	Piperalin	0.2753	329	5.42	30	1138260	166.07
51235-04-2	Hexazinone	0.4486	252	1.85	56	1213515	155.77
107534-96-3	Tebuconazole	0.1404	307	3.58	51	575025	328.73
63284-71-9	Nuarimol	0.0865	314	2.68	46	369270	511.89
96491-05-3	Thenylchlor	0.3149	323	3.59	58	981985	192.49
2425-06-1	Captafol	0.2073	347	2.4	63	617650	306.04
51338-27-3	Diclofop methyl	0.0989	340	4.24	45	438550	431.03
81406-37-3	Fluroxypyr-1-methylheptyl ester	0.0862	366	6.77	74	390525	484.03
2312-35-8	Propargite	0.3021	350	4.81	64	952425	198.47
83164-33-4	Diflufenican	0.4748	394	3.65	51	1160240	162.92
5259-88-1	Oxycarboxin	0.3186	267	1.8	84	854225	221.28
39300-45-3	Dinocap	0.5605	364	6.48	118	1427370	132.43
51-03-6	Piperonyl butoxide	0.3748	338	4.23	46	1175455	160.81
10453-86-8	Resmethrin	0.2084	338	6.63	39	939190	201.27
28434-01-7	Bioresmethrin	0.2016	338	6.63	39	916415	206.27
106325-08-0	Epoxiconazole	0.1662	329	3.44	43	626950	301.5
31251-03-3	Fluotrimazole	0.1145	379	5.68	31	501490	376.93
4726-14-1	Nitralin	0.2126	345	3.11	137	581025	325.33
999037-03-5	Dinocap II	0.6188	364	6.48	118	1535645	123.09

Table S5 (continued)

CASRN	Chemical	Fractional ion abundance	MW (g/mol)	logP	PSA (Å^2)	Response Factor at 500 pg/µL	Estimated LOQ (pg/µL)
88678-67-5	Pyributicarb	0.3534	330	5.21	67	1170250	161.53
22212-55-1	Benzoylprop ethyl	0.5325	365	4.77	47	1453310	130.07
36734-19-7	Iprodione	0.1189	329	2.22	73	404500	467.31
97-23-4	Dichlorophen	0.1471	268	4.62	40	794710	237.86
87-82-1	Hexabromobenzene	0.0939	546	5.85	0	482975	391.38
119-12-0	Pyridaphenthion	0.1267	340	3.08	102	420075	449.99
25006-32-0	Leptophos oxon	0.2442	394	4.73	45	755215	250.3
999054-03-4	Chlorthiophos sulfoxide	0.0770	376	3.51	106	275325	686.56
78-57-9	Menazon	0.4034	281	1.02	176	799800	236.34
7696-12-0	Tetramethrin I	0.4136	331	4.78	64	1275000	148.26
18181-80-1	Bromopropylate	0.2184	426	5.43	47	642265	294.31
999038-03-8	Dinocap III	0.7194	364	6.48	118	1716555	110.12
2104-64-5	EPN	0.2436	323	3.85	106	749500	252.21
55285-14-8	Carbosulfan	0.1101	380	6.05	67	433490	436.06
79127-80-3	Fenoxycarb	0.2198	301	4.3	60	853480	221.48
999050-03-2	Tetramethrin II	0.3412	331	4.78	64	1105955	170.92
82657-04-3	Bifenthrin	0.4697	422	7.3	26	1315645	143.68
24151-93-7	Piperophos	0.0953	353	4.3	106	356700	529.94
2275-14-1	Phenkapton	0.0914	376	5.85	111	348335	542.66
999039-03-1	Dinocap IV	0.6184	364	6.48	118	1534940	123.15
64257-84-7	Fenpropathrin	0.1361	349	5.48	59	562335	336.15
42576-02-3	Bifenox	0.1067	341	4.52	81	426955	442.73
84-61-7	Dicyclohexyl phthalate	0.3911	330	5.76	53	1372950	137.68
999053-03-1	Chlorthiophos sulfone	0.1518	392	4.31	112	449830	420.22
116-29-0	Tetradifon	0.1081	354	5.52	43	492635	383.7
26002-80-2	d-(cis-trans)-Phenothrin-I	0.2645	350	7.47	36	1137515	166.18
999034-03-6	d-(cis-trans)-Phenothrin-II	0.2694	350	7.47	36	1153110	163.93
65907-30-4	Furathiocarb	0.1482	382	4.52	94	471610	400.81
117-81-7	Bis(2-ethylhexyl)phthalate	0.3467	390	8.71	53	1130695	167.18
86-50-0	Azinphos-methyl	0.2249	317	2.54	131	614335	307.69
2310-17-0	Phosalone	0.1998	367	4.28	115	584655	323.31
21609-90-5	Leptophos	0.1996	410	6.25	60	619305	305.23
73250-68-7	Mefenacet	0.2160	298	3.85	71	788100	239.85
33089-61-1	Amitraz	0.1195	293	5.64	28	736685	256.59
68085-85-8	Cyhalothrin I (lambda)	0.1576	449	6.2	59	456375	414.19
60168-88-9	Fenarimol	0.1081	330	3.22	46	441815	427.84
2642-71-9	Azinphos-ethyl	0.1932	345	3.61	131	565240	334.42
13457-18-6	Pyrazophos	0.2350	373	3.01	126	598340	315.92
50-28-2	b-Estradiol	0.1510	272	4.13	40	748810	252.44
82558-50-7	Isoxaben	0.1942	332	3.25	74	627250	301.36
10311-84-9	Dialifos	0.2001	393	4.7	123	549920	343.74
55179-31-2	Bitertanol I	0.4299	337	4.02	60	1227230	154.03
19044-88-3	Oryzalin	0.1672	346	3.38	163	480210	393.63
999027-03-1	Bitertanol II	0.4318	337	4.02	60	1231145	153.54
96489-71-3	Pyridaben	0.4498	364	4.73	58	1238830	152.59
57-63-6	17a-Ethynylestradiol	0.1676	296	4.52	40	778955	242.67
56-72-4	Coumaphos	0.1425	362	3.86	96	464495	406.95
67747-09-5	Prochloraz	0.1716	375	3.98	47	587105	321.96
10540-29-1	Tamoxifen	0.4914	371	7.88	12	1987175	95.12
78-34-2	Dioxathion	0.1309	456	4.96	190	317465	595.43
119611-00-6	Fenbuconazole	0.2614	336	3.35	55	821120	230.21
68359-37-5	Cyfluthrin I	0.1188	433	6.29	59	390785	483.71
999028-03-4	Cyfluthrin II	0.1264	433	6.29	59	409105	462.05

Table S5 (continued)

CASRN	Chemical	Fractional ion abundance	MW (g/mol)	logP	PSA (Å^2)	Response Factor at 500 pg/µL	Estimated LOQ (pg/µL)
999029-03-7	Cyfluthrin III	0.1316	433	6.29	59	421495	448.47
999030-03-4	Cyfluthrin IV	0.1371	433	6.29	59	434630	434.92
52315-07-8	Cypermethrin-1	0.1159	415	6.27	59	408315	462.94
999031-03-7	Cypermethrin-2	0.1155	415	6.27	59	407270	464.13
76578-14-8	Quizalofop-ethyl	0.1411	372	4.65	71	493965	382.67
999032-03-0	Cypermethrin-3	0.1334	415	6.27	59	453000	417.28
999033-03-3	Cypermethrin-4	0.1335	415	6.27	59	453445	416.87
70124-77-5	Flucythrinate I	0.1373	451	6.18	69	398595	474.23
70-30-4	Hexachlorophene	0.0760	404	7.2	40	342210	552.38
491-80-5	5,7-Dihydroxy-4'-methoxyisoflavone	0.3390	284	3.14	76	1042160	181.38
999042-03-4	Flucythrinate II	0.1548	451	6.18	69	435530	434.02
59756-60-4	Fluridone	0.5010	329	3.7	20	1645705	114.86
362-05-0	2-Hydroxyestradiol	0.1845	288	3.53	61	714755	264.47
55512-33-9	Pyridate	0.1286	378	6.84	77	496770	380.51
51630-58-1	Fenvalerate	0.1035	419	6.68	59	373915	505.53
999041-03-1	Fenvalerate II	0.1010	419	6.68	59	367195	514.79
66230-04-4	Esfenvalerate	0.1003	419	6.68	59	365285	517.48
102851-06-9	Fluvalinate-tau-I	0.2458	502	7.31	71	509970	370.66
999044-03-0	Fluvalinate-tau-II	0.2275	502	7.31	71	481555	392.53
119446-68-3	Difenoconazol I	0.1901	405	4.92	58	586820	322.12
999036-03-2	Difenoconazol II	0.1886	405	4.92	58	583380	324.02
52918-63-5	Deltamethrin	0.1257	503	6.2	59	327380	577.39
3383-96-8	Temephos	0.2496	466	5.96	164	510285	370.43
108-95-2	Phenol	0.4693	94	1.48	20	1882680	100.4
95-57-8	2-Chlorophenol	0.3994	128	2.04	20	1821035	103.8
541-73-1	1,3-Dichlorobenzene	0.3316	146	3.42	0	3749510	50.41
98-16-8	3-Trifluormethylaniline	0.3831	161	2.39	26	1677025	112.72
88-75-5	2-Nitrophenol	0.3264	139	1.71	66	1123990	168.18
104-12-1	4-Chlorophenyl isocyanate	0.3341	153	3.42	29	2018685	93.64
105-67-9	2,4-Dimethylphenol	0.2539	122	2.4	20	1495880	126.37
367-21-5	3-Chloro-4-fluoroaniline	0.3751	145	2.14	26	1623140	116.46
99-88-7	4-Isopropylaniline	0.4630	135	2.28	26	2043330	92.51
99-49-0	Carvone	0.2334	150	2.27	17	1251480	151.04
24549-06-2	2-ethyl-6-methylaniline	0.4456	135	2.39	26	2053440	92.05
3964-56-5	Profenofos metabolite (4-Bromo-2-chlorophenol)	0.2702	206	3	20	1344040	140.64
591-27-5	3-Aminophenol	0.4322	109	0.34	46	985810	191.75
35421-08-0	4-Chloro-3-methylphenol	0.2503	142	1.75	20	1133955	166.7
89-83-8	Thymol	0.4301	150	3.28	20	2528365	74.76
95-69-2	4-Chloro-2-methylaniline	0.2552	141	2.22	26	1265550	149.36
137-17-7	2,4,5-Trimethylaniline	0.2695	135	2.32	26	1386450	136.34
106-40-1	4-Bromoaniline	0.2703	171	2.05	26	1156610	163.43
1563-38-8	Carbofuran-7-phenol	0.1917	164	2.23	29	938815	201.35
102-36-3	Diuron Metabolite [3,4-Dichlorophenyl isocyanate]	0.2120	187	4.01	29	1408515	134.2
3228-03-3	Promecarb artifact [5-isopropyl-3-methylphenol]	0.3636	150	3.28	20	2233265	84.64
87-40-1	2,4,6-Trichloroanisole	0.1421	210	3.95	9	1126600	167.79
97-53-0	Eugenol	0.2237	164	2.2	29	1044140	181.04
50375-10-5	2,3,6-Trichloroanisole	0.1304	210	3.87	9	1041040	181.57
999058-03-6	Phenol, 2-(1,3-dioxolan-2-yl)-	0.2676	166	0.89	39	812360	232.69
626-39-1	1,3,5-Tribromobenzene	0.2137	312	4.39	0	1658075	114
5345-54-0	3-Chloro-4-methoxyaniline	0.3121	157	1.57	35	1118505	169
85-41-6	Phthalimide	0.2969	147	0.8	50	834930	226.4
27813-21-4	Tetrahydrophthalimide, cis-1,2,3,6-	0.2785	151	1.33	46	930600	203.12
33704-61-9	Cashmeran	0.1859	206	4.06	17	1290865	146.43

Table S5 (continued)

CASRN	Chemical	Fractional ion abundance	MW (g/mol)	logP	PSA (Å^2)	Response Factor at 500 pg/µL	Estimated LOQ (pg/µL)
90-15-3	1-Hydroxynaphthalene	0.3039	144	2.71	20	1712570	110.38
34014-18-1	Tebuthiuron	0.3705	228	1.79	86	1006860	187.74
100-02-7	4-Nitrophenol	0.3081	139	1.57	66	1033605	182.88
2425-10-7	XMC (3,4-Dimethylphenyl N-methylcarbamate)	0.3466	179	2.09	38	1290585	146.47
33089-74-6	Methanimidamide, N-(2,4-dimethylphenyl)-N'-methyl-	0.1678	162	2.66	24	986315	191.65
636-30-6	2,4,5-Trichloroaniline	0.2440	195	3.42	26	1352215	139.79
98-10-2	Benzenesulfonamide	0.3022	157	0.62	70	755505	250.2
626-15-3	alpha, alpha-Dibromo-m-xylene	0.2377	262	3.62	0	1922690	98.31
2655-14-3	XMC (3,5-Dimethylphenyl N-methylcarbamate)	0.3764	179	2.09	38	1371570	137.82
136-85-6	Tolyltriazole [1H-Benzotriazole, 5-methyl-]	0.2159	133	1.8	42	933445	202.51
999002-03-8	Benzoximate metabolite	0.2025	213	3.8	48	1067060	177.15
77-40-7	bisphenol B	0.1724	242	3.96	40	897755	210.56
2600-69-3	Phorate-oxon	0.1177	244	2.07	96	431360	438.21
607-99-8	2,4,6-Tribromoanisole	0.1428	342	4.17	9	741140	255.05
54593-83-8	Chlorethoxyfos	0.1118	334	3.97	70	443670	426.05
4710-17-2	Dichlofluanid metabolite (DMSA)	0.2349	200	0.58	58	629790	300.14
300-76-5	Naled	0.3659	378	1.86	55	896885	210.76
16954-69-1	Methabenzthiazuron [decomposition product]	0.2173	164	2.23	53	930240	203.2
6190-65-4	Atrazine-desethyl	0.2915	187	0.18	73	653330	289.33
879-39-0	2,3,4,5-Tertrachloronitrobenzene	0.0756	259	3.94	46	446890	422.98
3811-49-2	Dioxabenzofos	0.2627	216	2.15	70	882225	214.26
2008-58-4	2,6-Dichlorobenzamide	0.2610	189	1.2	44	820485	230.39
30125-63-4	Terbuthylazine-desethyl	0.3322	201	-0.1	73	681240	277.48
95465-99-9	Cadusafos	0.1662	270	4.28	87	723025	261.44
95-06-7	Sulfallate	0.5089	223	4.19	61	2085890	90.62
3740-92-9	Fenclorim	0.2034	224	4.02	26	1181600	159.98
13171-00-1	Celestolide	0.3103	244	5.51	17	2019575	93.6
41205-21-4	Fluoroimide	0.1978	259	2.3	37	749090	252.34
999016-03-4	Empenthrin IV	0.1451	274	6.35	26	1029625	183.59
999017-03-7	Empenthrin V	0.2386	274	6.35	26	1486915	127.13
152-16-9	Octamethyl pyrophosphoramide	0.1396	286	-3.77	76	240315	786.58
4228-88-0	Fenazaflor metabolite	0.1173	254	3.46	29	634430	297.95
92-67-1	4-Aminobiphenyl	0.4895	169	2.77	26	2152410	87.82
37019-18-4	Sebuthylazine-desethyl	0.4112	201	2.04	77	1221385	154.76
66840-71-9	Tolylfluanid metabolite (DMST)	0.2538	214	1.04	58	723775	261.17
15323-35-0	Phantolide	0.3626	244	5.8	17	2370825	79.73
120-51-4	Benzyl benzoate	0.3504	212	3.97	26	1840050	102.73
2282-34-0	Bufencarb	0.2623	221	3.57	38	1249005	151.34
1517-22-2	phenanthrene-d10	0.5192	188	4.68	0	5673600	33.32
85-01-8	phenanthrene	0.5362	178	4.68	0	6163050	30.67
962-58-3	Diazinon-oxon	0.2290	288	2.07	80	676970	279.23
3734-48-3	Chlordene, trans-	0.3435	336	5.44	0	2369825	79.76
771-51-7	3-Indolylacetonitrile	0.3251	156	1.37	40	1070880	176.52
502-72-7	Cyclopentadecanone	0.0789	224	5.84	17	867960	217.78
4147-57-3	2-Amino-4-isopropylamino-6-methylthio-1,3,5-triazine	0.2392	199	2.15	102	801105	235.96
6108-10-7	BHC epsilon isomer	0.1338	288	3.99	0	1214150	155.69
79538-32-2	Tefluthrin, cis-	0.4861	418	5.78	26	1323890	142.78
106-02-5	Exaltolide [15-Pentadecanolide]	0.0838	240	5.44	26	722445	261.65
495-48-7	Azoxybenzene	0.2856	198	4.09	41	1597835	118.3
83-66-9	Musk amberette	0.2009	268	3.71	101	757325	249.6
1929-87-9	Methyl (2-naphthoxy)acetate	0.3043	216	2.64	36	1205795	156.77
34849-42-8	Cyclafuramid	0.3186	221	3.2	42	1326580	142.49
58-08-2	Caffeine	0.3812	194	-0.13	58	776720	243.37

Table S5 (continued)

CASRN	Chemical	Fractional ion abundance	MW (g/mol)	logP	PSA (Å^2)	Response Factor at 500 pg/µL	Estimated LOQ (pg/µL)
96182-53-5	Tebupirimifos	0.1129	318	3.3	95	417130	453.16
98730-04-2	Benoxacor	0.2332	259	3.19	30	993880	190.19
68140-48-7	Traseolide	0.3644	258	6.14	17	2315815	81.62
24602-86-6	Tridemorph , 4-tridecyl-	0.0627	297	7.03	12	599710	315.2
101-76-8	p,p'-DDM [bis(4-chlorophenyl)methane]	0.2205	236	5.4	0	2652170	71.27
3983-45-7	Fenchlorphos-oxon	0.2888	304	3.03	55	922270	204.96
175217-20-6	Silthiopham	0.4392	267	4.88	57	1746245	108.25
83-67-0	Theobromine	0.4329	180	-2.08	71	521755	362.29
84-69-5	Diisobutyl phthalate	0.6448	278	4.46	53	2130195	88.74
104-40-5	4-Nonylphenol	0.6627	220	6.19	20	4451410	42.46
81-15-2	Musk xylene	0.2671	297	3.83	137	824390	229.29
1506-02-1	Tonalide	0.3050	258	6.37	17	2097515	90.12
41096-46-2	Hydroprene	0.2874	266	6.83	26	1902275	99.37
2255-17-6	Fenitrothion-oxon	0.2644	261	1.71	100	719435	262.74
26244-33-7	Ethofumesate, 2-Keto	0.4575	256	1.45	78	1091775	173.14
87674-68-8	Dimethenamid	0.2365	275	2.15	58	754850	250.42
56425-91-3	Flurprimidol	0.5023	312	2.37	55	1281305	147.53
118134-30-8	Spiroxamine I	0.6789	297	4.88	22	2508075	75.37
116-66-5	Musk Moskene	0.3119	278	5.26	92	1251385	151.05
136-45-8	Dipropyl isocinchomeronate	0.3632	251	3.09	65	1219075	155.06
135158-54-2	Azibenzolar-S-methyl	0.1834	210	2.18	96	652865	289.53
149508-90-7	Simeconazole	0.2042	293	2.46	51	695780	271.68
61676-87-7	Cymiazole	0.1965	218	3.32	41	961865	196.52
999003-03-1	Flurochloridone, deschloro-	0.3045	277			0	
118712-89-3	Transfluthrin	0.3805	370	5.4	26	1275990	148.14
86763-47-5	Propisochlor	0.2647	283	3.27	30	1034485	182.73
999004-03-4	Cyclohexanol, 2-(4-tert-butylphenoxy)	0.5474	248	4.14	29	2237410	84.48
127-90-2	Bis(2,3,3,3-tetrachloropropyl) ether	0.1052	374	6.17	9	601020	314.51
111246-15-2	Fipronil, Desulfinyl-	0.1788	388	7.57	68	640070	295.32
52888-80-9	Prosulfocarb	0.2581	251	3.99	46	1148275	164.62
67306-00-7	Fenpropidin	0.6878	273	5.87	3	4279475	44.17
34622-58-7	Orbencarb	0.3299	257	3.53	46	1261655	149.82
145-39-1	Musk Tibetene (Moschustibeten)	0.2495	266	4.68	92	1035670	182.52
999026-03-8	Spiroxamine II	0.6906	297	4.88	22	2540015	74.42
1825-19-0	pentachlorophenyl methyl sulfide	0.1244	294	5.81	25	784615	240.92
39811-17-1	5-Phenyl-o-anisidine	0.3301	199	2.78	35	1388475	136.14
76608-88-3	Triapenthenol	0.4348	263	3.62	51	1511255	125.08
29091-21-2	Prodiamine	0.1845	350	6.92	121	683400	276.6
2588-03-6	Phorate sulfoxide	0.1745	276	1.51	122	489080	386.49
999005-03-7	Terbufos-oxon-sulfone	0.0913	304	2.72	113	337310	560.4
14214-32-5	Difenoxuron	0.4283	286	2.02	51	1157735	163.27
2588-04-7	Phorate sulfone	0.1902	292	1.78	128	523790	360.88
63-74-1	Sulfanilamide	0.2084	172	-0.72	95	391150	483.26
117718-60-2	Thiazopyr	0.0865	396	3.52	77	304375	621.04
40341-04-6	Rabenzazole	0.3092	212	3.47	47	1380735	136.9
24353-61-5	Isocarbophos	0.1816	289	1.66	113	512095	369.13
142459-58-3	Flufenacet	0.1555	363	3.98	84	509080	371.31
106848-93-5	Isofenphos-oxon	0.2578	329	3.07	84	750580	251.84
141112-29-0	Isoxaflutole	0.2113	359	1.67	86	558440	338.49
112281-77-3	Tetraconazole	0.3384	371	3.19	40	965370	195.81
98886-44-3	Fosthiazate I	0.1250	283	0.94	107	362410	521.58
30043-49-3	Ethidimuron	0.1823	264	-0.5	129	385445	490.41
999018-03-0	Fosthiazate II	0.1002	283	0.94	107	307765	614.19

Table S5 (continued)

CASRN	Chemical	Fractional ion abundance	MW (g/mol)	logP	PSA (Å^2)	Response Factor at 500 pg/µL	Estimated LOQ (pg/µL)
121552-61-2	Cyprodinil	0.4055	225	4	38	1832400	103.16
92-84-2	Phenothiazine	0.3702	199	4.15	37	1985005	95.23
2550-75-6	Chlorbicyclen	0.0579	394	5.84	0	527635	358.25
206-44-0	fluoranthene	0.5266	202	5.17	0	5858400	32.27
56070-16-7	Terbufos-sulfone	0.1614	320	2.48	128	477545	395.83
18708-87-7	Chlorfenvinphos, cis-	0.1619	358	4.51	55	590025	320.37
61432-55-1	Dimepiperate	0.2712	263	3.97	46	1138195	166.08
28159-98-0	Irgarol	0.1725	253	1.12	86	499145	378.7
18708-86-6	Chlorfenvinphos, trans-	0.1642	358	4.51	55	596245	317.03
79622-59-6	Fluazinam	0.1001	464	8.19	117	275545	686.01
19480-43-4	MCPA-butoxyethyl ester	0.0988	300	3.89	45	476690	396.54
999006-03-0	Zoxamide decomposition product	0.1262	650			0	
113614-08-7	Beflubutamid	0.2795	355	4.96	38	976210	193.63
3380-34-5	Triclosan	0.1304	288	5.17	29	757120	249.67
1022-22-6	DDMU [1-Chloro-2,2-bis(4'-chlorophenyl)ethylene]	0.1563	282	6.04	0	1776360	106.41
101-80-4	4,4'-Oxydianiline	0.4182	200	0.51	61	943025	200.45
23031-36-9	Prallethrin, cis-	0.3426	300	4.38	43	1268435	149.02
92-87-5	Benzidine	0.5299	184	1.56	52	1468645	128.71
7292-16-2	Propaphos	0.2256	304	3.79	80	778745	242.73
999023-03-9	Prallethrin, trans-	0.3310	300	4.38	43	1236630	152.86
62850-32-2	Fenothiocarb	0.5045	253	3.28	55	1635405	115.58
70193-21-4	Trichlamide	0.3693	339	5.69	59	1239735	152.47
69806-40-2	Haloxyfop-methyl	0.1400	375	2.87	58	460920	410.11
4640-01-1	Triclosan-methyl	0.1465	302	4.74	18	807095	234.21
2497-06-5	Disulfoton sulfone	0.2104	306	1.87	128	559995	337.55
110235-47-7	Mepanipyrim	0.4579	223	3.28	38	1777955	106.32
58580-14-6	Bromfenvinphos-(E)	0.1498	402	4.68	55	496625	380.62
112143-82-5	Triazamate	0.5195	314	1.93	103	1128755	167.47
58580-13-5	Bromfenvinphos-(Z)	0.1775	402	4.68	55	563130	335.67
117428-22-5	Picoxystrobin	0.1884	367	4.48	58	636315	297.06
174514-07-9	Fluazolate	0.1220	442	5.47	44	403350	468.64
133408-50-1	Metominostrobin (E)	0.1883	284	4.39	60	814535	232.07
131341-86-1	Fludioxonil	0.3826	248	3.67	58	1422035	132.93
6552-21-2	Fensulfothion-oxon	0.1409	292	0.81	91	399240	473.46
999007-03-3	Prothioconazole-desthio	0.3469	311	2.42	51	993500	190.26
140923-25-7	Iprovalicarb I	0.1827	320	3.56	67	643750	293.63
140-57-8	Aramite	0.0862	334	4.12	64	375985	502.75
3988-03-2	p,p'-Dibromobenzophenone	0.1913	338	5.06	17	898920	210.28
142534-71-2	Toxaphene Parlar 26	0.0365	410	5.66	0	353020	535.46
69969-22-8	Imazamethabenz-methyl I	0.1384	288	3.02	68	534835	353.43
41483-43-6	Bupirimate	0.1869	316	2.7	93	577755	327.18
130000-40-7	Thifluzamide	0.2331	526	4.09	79	494130	382.55
999022-03-6	Metominostrobin (Z)	0.1426	284	4.39	60	663455	284.91
94361-06-5	Cyproconazole	0.2407	291	2.7	51	810355	233.27
143390-89-0	Kresoxim-methyl	0.2303	313	4.34	57	860705	219.62
999020-03-0	Iprovalicarb II	0.1846	320	3.56	67	648840	291.33
6132-17-8	Fensulfothion-oxon -sulfone	0.0858	308	0	97	251090	752.83
999009-03-9	Aramite II	0.1161	334	4.12	64	468575	403.41
999019-03-3	Imazamethabenz-methyl II	0.0505	288	3.02	68	253980	744.26
104030-54-8	Carpropamid	0.0940	333	4.26	29	464635	406.83
122453-73-0	Chlorfenapyr	0.4018	406	5.16	38	1101110	171.67
180409-60-3	Cyflufenamid	0.1245	412	5.71	54	435255	434.29
115852-48-7	Fenoxanil	0.1207	328	3.86	62	482815	391.51

Table S5 (continued)

CASRN	Chemical	Fractional ion abundance	MW (g/mol)	logP	PSA (Å^2)	Response Factor at 500 pg/µL	Estimated LOQ (pg/µL)
97-56-3	ortho-Aminoazotoluene	0.2796	225	4.33	51	1403615	134.67
5103-73-1	cis-Nonachlor	0.1283	440	5.9	0	817425	231.25
3761-42-0	Fenthion-sulfone	0.1882	310	1.83	112	522785	361.58
74070-46-5	Aclonifen	0.2685	264	4.37	81	1079860	175.05
999024-03-2	Pyriminobac-methyl (Z)	0.3169	361	2.1	101	750490	251.87
14255-72-2	fensulfothion-sulfone	0.1331	324	2.43	112	419030	451.1
7082-99-7	Chlorbenside sulfone	0.5405	300	3.5	43	1620220	116.67
163520-33-0	Isoxadifen-ethyl	0.0820	295	3.1	48	382525	494.15
58810-48-3	Ofurace	0.0795	281	0.93	47	298475	633.3
121-21-1	Pyrethrin I	0.2503	328	5.49	43	1008340	187.46
124495-18-7	Quinoxyfen	0.2552	307	6.29	22	1348210	140.21
2132-70-9	Methoxychlor olefin	0.1857	308	5.21	18	985865	191.74
63837-33-2	Diofenolan I	0.2370	300	3.73	37	925545	204.23
126833-17-8	Fenhexamid	0.3370	301	4.02	49	1176755	160.63
84-75-3	Di-n-hexyl phthalate	0.7102	334	6.95	53	2305635	81.98
128639-02-1	Carfentrazone-ethyl	0.1034	411	3.21	62	347065	544.65
999013-03-5	Diofenolan II	0.1621	300	3.73	37	698815	270.5
105512-06-9	Clodinafop-propargyl	0.1346	349	2.33	58	451710	418.47
141517-21-7	Trifloxystrobin	0.1709	408	5.11	69	525100	359.98
129630-19-9	Pyraflufen-ethyl	0.1383	412	3.33	63	429570	440.04
136191-64-5	Pyriminobac-methyl (E)	0.3161	361	2.1	101	749090	252.34
115-86-6	TPP	0.2282	326	4.1	55	809245	233.58
66860-80-8	Toxaphene Parlar 50	0.0223	445	6.08	0	220795	856.12
78-51-3	Tris(2-butoxyethyl) phosphate	0.1300	398	4.3	82	416680	453.65
999025-03-5	Resmethrine II	0.2237	382	6.63	39	817750	231.16
156052-68-5	Zoxamide	0.2840	335	4.84	46	1012760	186.65
135590-91-9	Mefenpyr-diethyl	0.2110	372	3.2	68	621210	304.29
74738-17-3	Fenpiclonil	0.2592	236	3.88	40	1224995	154.31
283594-90-1	Spiromesifen	0.2015	370	6.07	53	733070	257.86
31972-43-7	Fenamiphos sulfoxide	0.1341	319	1.39	94	398865	473.91
116255-48-2	Bromuconazole I	0.1843	375	2.73	40	597395	316.42
842-07-9	Sudan I	0.2146	248	4.24	45	1055790	179.04
31972-44-8	Fenamiphos-sulfone	0.2992	335	1.75	100	724920	260.76
131086-42-5	Ethoxyfen-ethyl	0.3146	450	5.68	62	749790	252.11
149961-52-4	Dimoxystrobin	0.2893	326	5.08	60	1032345	183.1
78-42-2	Tris(2-ethylhexyl) phosphate	0.4782	434	10.09	55	1164870	162.27
117-83-9	Bis(2-butoxyethyl) phthalate	0.1688	366	3.99	71	553085	341.77
137641-05-5	Picolinafen	0.2209	376	4.53	55	706100	267.71
99607-70-2	Cloquintocet-mexyl	0.4666	335	5.01	48	1470745	128.52
119-90-4	3,3'-Dimethoxybenzidine	0.3450	244	1.58	71	931540	202.92
153233-91-9	Etoxazole	0.0674	359	5.85	31	367925	513.76
122836-35-5	Sulfentrazone	0.1055	386	2.21	90	331160	570.8
125116-23-6	Metconazole I	0.1162	319	3.3	51	472625	399.95
161326-34-7	Fenamidone	0.1653	311	3.61	70	610090	309.84
120928-09-8	Fenazaquin	0.4407	306	5.54	35	1741870	108.52
119168-77-3	Tebufenpyrad	0.1420	333	3.9	47	564765	334.7
999010-03-6	Bromuconazole II	0.2092	375	2.73	40	656080	288.12
64249-01-0	Anilofos	0.1204	367	4.2	106	405970	465.62
103112-35-2	Fenchlorazole-ethyl	0.0562	401	5.57	57	247065	765.09
51186-88-0	Phenothrin I	0.2549	350	7.47	36	1106930	170.77
164159-06-5	Toxaphene Parlar 62	0.0274	445			0	
26046-85-5	Phenothrin II	0.2820	350	7.47	36	1192775	158.48
84-62-8	Diphenyl phthalate	0.5828	318	4.41	53	1714690	110.24

Table S5 (continued)

CASRN	Chemical	Fractional ion abundance	MW (g/mol)	logP	PSA (Å^2)	Response Factor at 500 pg/µL	Estimated LOQ (pg/µL)
299-45-6	Potasan	0.2125	328	3.66	96	669365	282.4
96525-23-4	Flurtamone	0.2060	333	2.27	38	677405	279.05
95737-68-1	Pyriproxyfen	0.5477	321	4.84	41	1759950	107.4
122008-85-9	Cyhalofop-butyl	0.1941	357	4.4	69	646430	292.42
52570-16-8	Naproanilide	0.1814	291	4.31	38	828055	228.28
3861-47-0	Ioxynil octanoate	0.4065	497	6.18	50	816310	231.56
1420-06-0	Trifenmorph	0.4621	329	4.24	12	1761000	107.34
77501-63-4	Lactofen	0.1254	461	4.84	108	334825	564.56
135186-78-6	Pyriftalid	0.1469	318	2.25	96	459955	410.97
101007-06-1	Acrinathrin	0.1190	541	5.99	86	267500	706.65
77501-90-7	Fluoroglycofen-ethyl	0.0698	447	4.49	108	224115	843.43
82560-54-1	Benfuracarb	0.0974	410	4.54	94	321645	587.69
78-30-8	Tricresylphosphate, ortho-	0.2502	368	5.48	55	834115	226.62
1374255-23-	Pyraclofos	0.1742	360	4.01	88	554595	340.84
220899-03-6	Metrafenone	0.0914	408	5.19	54	345355	547.34
66441-23-4	Fenoxaprop-ethyl	0.1868	361	4.78	71	632565	298.83
563-04-2	Tricresylphosphate, meta-	0.2453	368	5.48	55	821985	229.96
148477-71-8	Spirodiclofen	0.3976	410	6.47	53	1059150	178.47
3118-97-6	Sudan II	0.1428	276	4.56	41	744575	253.87
78-32-0	Tri-p-tolyl phosphate	0.2353	368	5.48	55	797115	237.14
136426-54-5	Fluquinconazole	0.3767	375	2.79	63	940940	200.89
117-84-0	Di-n-octyl phthalate	0.6448	390	9.08	53	1815485	104.12
999008-03-6	2,4,5-Trichloro-p-terphenyl	0.2184	332	7.1	0	1966255	96.14
134605-64-4	Butafenacil	0.4401	474	3.63	93	850125	222.35
205-99-2	benzo[b]fluoranthene	0.3274	252	6.4	0	3793515	49.83
125306-83-4	Cafenstrole	0.4382	350	3.21	94	1054620	179.24
61576-99-6	2,3,5,6-Tetrachloro-p-terphenyl	0.2146	366	7.35	0	1672010	113.05
361377-29-9	Fluoxastrobin cis-	0.2207	458	5.34	97	521910	362.18
188425-85-6	Boscalid (Nicobifen)	0.2726	342	5.72	45	1027440	183.98
111872-58-3	Halfenprox	0.1853	476	8.34	28	526240	359.2
121227-99-4	Cekafix	0.0986	406	4.02	96	321555	587.85
80844-07-1	Ethofenprox	0.5714	376	7.34	28	1836155	102.95
57960-19-7	Acequinocyl	0.3350	384	8.45	60	1101700	171.58
105024-66-6	Silafluofen	0.2327	408	9.63	18	944170	200.2
105779-78-0	Pyrimidifen	0.5097	377	5.34	56	1355210	139.48
84-76-4	Di-n-nonyl phthalate	0.5855	418	10.14	53	1491995	126.69
87546-18-7	Flumiclorac-pentyl	0.1723	423	4.27	73	492665	383.68
131860-33-8	Azoxystrobin	0.2592	403	5.13	104	676220	279.54
131807-57-3	Famoxadon	0.1652	374	4.76	68	558995	338.16
999012-03-2	Dimethomorph-(Z)	0.2819	387	3.71	48	811285	233
129558-76-5	Tolfenpyrad	0.1289	383	4.66	56	466370	405.31
110488-70-5	Dimethomorph-(E)	0.2540	387	3.71	48	751070	251.68
193-39-5	indeno[1,2,3-cd]pyrene	0.4647	276	6.89	0	4558395	41.47
53-70-3	dibenzo[a,h]anthracene	0.5767	278	7.14	0	5451200	34.68
142891-20-1	Cinidon-ethyl	0.2432	393	3.75	64	684745	276.05
83-79-4	Rotenone	0.4003	394	4.65	63	1024740	184.46
98-53-3	4-tert-butylcyclohexanone	0.2085	154	2.48	17	1202670	157.17
29878-31-7	Tolyltriazole [1H-Benzotriazole, 4-methyl-]	0.2560	133	1.8	42	1058645	178.56
207-08-9	benzo[k]fluoranthene	0.4936	252	6.4	0	5138050	36.79
125225-28-7	Ipconazole	0.1133	333	3.64	51	461635	409.47
131983-72-7	Triticonazole	0.1293	317	3.07	51	503335	375.55
999021-03-3	Metconazole II	0.1107	319	3.3	51	456085	414.46
23576-24-1	Norflurazon, Desmethyl-	0.2070	289	2.03	59	657555	287.47

Table S5 (continued)

CASRN	Chemical	Fractional ion abundance	MW (g/mol)	logP	PSA (Å^2)	Response Factor at 500 pg/µL	Estimated LOQ (pg/µL)
54406-48-3	Empenthrin I	0.1956	274	6.35	26	1283645	147.26
999014-03-8	Empenthrin II	0.2047	274	6.35	26	1327670	142.37
999015-03-1	Empenthrin III	0.2056	274	6.35	26	1331705	141.94
82-66-6	Diphacinone	0.2994	340	4.38	51	983640	192.17
71561-11-0	Pyrazoxyfen	0.2294	402	4.27	61	659860	286.47
3766-81-2	Fenobucarb	0.4543	207.3	3.04	38	1779520	106.22
114-26-1	Propoxur	0.5599	209	2.56	51	1790410	105.58
57966-95-7	Cymoxanil	0.2284	198	0.67	104	569295	332.04
2164-17-2	Fluometuron	0.4715	232	2.36	32	1556235	121.46
1746-81-2	Monolinuron	0.2798	214	2.3	42	1041285	181.53
4658-28-0	Aziprotryne	0.0841	225	0.18	88	253815	744.74
80-06-8	Chlorfenethol	0.2003	266	4.28	20	1079405	175.12
42874-03-3	Oxyfluorfen	0.1655	361	4.34	64	573465	329.62
35575-96-3	Azamethiphos	0.1310	324	0.82	113	360030	525.03
135410-20-7	acetamiprid	0.1097	222	0.62	52	365430	517.27
39515-40-7	Cyphenothrin cis-	0.3034	375	6.59	59	981000	192.69
999011-03-9	Cyphenothrin trans-	0.3360	375	6.59	59	1057930	178.68
1229-55-6	Sudan Red	0.1499	278	4.99	54	771215	245.1
117337-19-6	Fluthiacet-methyl	0.1871	403	1.4	113	464310	407.11
139920-32-4	Diclocymet I	0.1309	312	3.33	53	523005	361.42
999059-03-9	Diclocymet II	0.1281	312	3.33	53	514660	367.29
115-32-2	p,p'-Dicofol	0.1634	368	5.74	20	731865	258.28
229977-93-9	Fluacrypyrim	0.1339	426	5.1	80	406185	465.37
103361-09-7	Flumioxazin	0.2300	354	2.29	67	648810	291.35
121776-33-8	Furilazole	0.1293	277	2.33	43	518055	364.88
76703-62-3	Cyhalothrin (Gamma)	0.1767	449	6.2	59	496525	380.7
86598-92-7	Imibenconazole	0.2023	410	5.69	68	602625	313.67
199338-48-2	Imibenconazole-desbenzyl	0.1829	270	2.28	75	612660	308.53
158474-72-7	Prohydrojasmon I	0.1593	254	3.56	43	755315	250.26
999060-03-6	Prohydrojasmon II	0.1679	254	3.56	43	785105	240.77
4466-14-2	Jasmolin I	0.2926	330	5.97	43	1167645	161.89
25402-06-6	Cinerin I	0.2744	316	5.44	43	1126705	167.77
2044-54-2	Cinerin II	0.1652	360	4.38	70	566860	333.46
1172-63-0	Jasmolin II	0.1582	374	4.92	70	543035	348.09
121-29-9	Pyrethrin II	0.0798	372	4.43	70	321150	588.59
28044-83-9	Heptachlor epoxide isomer A	0.0478	386	5.47	13	293170	644.78
71363-52-5	Dimethylvinphos(E)	0.2083	330	3.45	55	709355	266.48
137219-34-2	9-Fluorenone-d8	0.3708	188.11	3.58	17	2104775	89.81
635-12-1	1,4-Anthraquinone	0.2349	208.2	3.02	34	1105835	170.94
82-86-0	Acenaphthenequinone	0.2345	182.04	2.28	34	1021070	185.13
84-11-7	9,10-Phenanthrenequinone	0.2464	208.05	3.13	34	1170855	161.44
569-15-3	Phenanthrene-1,4-dione	0.2169	208.05	3.02	34	1042925	181.25
1719-03-5	chrysene-d12	0.4177	240.17	5.91	0	4514320	41.87
109699-80-1	Benzo(c)phenanthrene(1,4)quinone	0.2022	258.07	4.25	34	1020580	185.22
3067-13-8	1,6-Benzo(a)pyrene-quinone	0.1562	282.07	4.81	34	827270	228.5
65199-11-3	Benzo(a)pyrene-7,8-dione	0.2070	232	3.51	34	1016910	185.88
1146-65-2	naphthalene-d8	0.5123	136.112	3.45	0	5507700	34.32
91-57-6	2-methylnaphthalene	0.3100	142.078	3.91	0	4196550	45.04
575-43-9	1,6-dimethylnaphthalene	0.2558	156.09	4.37	0	3783465	49.96
93951-97-4	acenaphthylene-d8	0.4824	160.112	4.26	0	5729950	32.99
93951-69-0	fluoranthene-d10	0.4458	212.14	5.17	0	4884530	38.7
63466-71-7	benzo[a]pyrene-d12	0.3968	264.168	6.4	0	4073800	46.4
93951-66-7	benzo[ghi]perylene-d12	0.4320	288.17	6.89	0	4026970	46.94

Table S5 (continued)

CASRN	Chemical	Fractional ion abundance	MW (g/mol)	logP	PSA (Å^2)	Response Factor at 500 pg/µL	Estimated LOQ (pg/µL)
1520-96-3	perylene-d12	0.3953	264.17	6.4	0	4061845	46.54
86-57-7	1-Nitronaphthalene	0.1644	173.047	3.18	46	949320	199.12
581-89-5	2-Nitronaphthalene	0.2614	173.047	3.18	46	1337620	141.32
2113-58-8	3-Nitrobiphenyl	0.2145	199.063	3.51	46	1119795	168.81
92-93-3	4-Nitrobiphenyl	0.1967	199.063	3.59	46	1067915	177.01
602-87-9	5-Nitroacenaphthene	0.1786	199.063	3.92	46	1064130	177.64
607-57-8	2-Nitrofluorene	0.1877	211.063	3.89	46	1042030	181.4
602-60-8	9-Nitroanthracene	0.1219	223.063	4.41	46	790820	239.03
954-46-1	9-Nitrophenanthrene	0.1531	223.063	4.41	46	936110	201.93
17024-19-0	3-Nitrophenanthrene	0.1898	223.063	4.41	46	1097270	172.27
3586-69-4	2-Nitroanthracene	0.1770	223.063	4.41	46	1041940	181.42
892-21-7	3-Nitrofluoranthene	0.1416	247.063	4.9	46	858635	220.15
5522-43-0	1-Nitropyrene	0.1425	247.063	4.9	46	862700	219.11
789-07-1	2-Nitropyrene	0.2525	247.06	4.9	46	1316760	143.55
20268-51-3	7-Nitrobenz(a)anthracene	0.0853	273.079	5.64	46	579640	326.11
7496-02-8	6-Nitrochrysene	0.1203	273.08	5.64	46	747280	252.95
1711-34-9	3-Nitrobenzanthrone	0.1116	275.06	4.54	63	577510	327.31
75321-20-9	1,3-Dinitropyrene	0.1478	292.05	4.57	92	628015	300.99
42397-64-8	1,6-Dinitropyrene	0.1124	292.05	4.63	92	516565	365.93
42397-65-9	1,8-Dinitropyrene	0.1118	292.05	4.63	92	514430	367.45
132482-44-1	6-Nitrobenzo(a)pyrene	0.1534	297.08	6.13	46	843555	224.08
NO CAS	5-Nitroacenaphthene-D9	0.1611	208.119	3.92	46	947935	199.41
128008-87-7	2-Nitrofluorene-D9	0.2267	220.119	3.89	46	1154125	163.78
93487-20-8	1-Nitropyrene-D9	0.1529	256.12	4.9	46	872670	216.61
203805-92-9	6-Nitrochrysene-D11	0.1265	284.15	5.64	46	736135	256.78
86-00-0	2-Nitrobiphenyl	0.0985	199.063	3.52	46	631220	299.46
613-12-7	2-methylanthracene	0.3209	192.09	5.14	0	4287270	44.09
832-69-9	1-methylphenanthrene	0.3351	192.09	5.14	0	4426675	42.7
93952-19-3	4,4'-DDE-d4	0.1146	323.987	6.37	0	1193835	158.34
93952-23-9	PCB 77 - d6	0.1768	295.959	6	0	1809150	104.48
15067-26-2	Acenaphthene-d10	0.2336	164.14	4.19	0	3214965	58.8
571-61-9	1,5-dimethylnaphthalene	0.2401	156.09	4.37	0	3610815	52.35
205-82-3	benzo[j]fluoranthene	0.3650	252.09	6.4	0	4108105	46.01
191-26-4	anthanthrene	0.3517	276.09	6.89	0	3708340	50.97
5385-75-1	dibenzo[a,e]fluoranthene	0.2086	302.11	7.63	0	2350175	80.43
192-65-4	dibenzo[a,e]pyrene	0.2579	302.11	7.63	0	2749455	68.75
217-59-4	triphenylene	0.3677	228.09	5.91	0	4411165	42.85
238-84-6	benzo[a]fluorene	0.3071	216.09	5.39	0	3778315	50.03
243-17-4	benzo[b]fluorene	0.2859	216.09	5.39	0	3583280	52.75
3697-24-3	5-methylchrysene	0.2555	242.11	6.37	0	3337610	56.64
202-33-5	benz[j]and[e]aceanthrylene	0.3672	252.09	6.73	0	4333245	43.62
5385-22-8	Naphtho[1,2-b]fluoranthene	0.3613	302.11	7.63	0	3527810	53.58
205-83-4	Naphtho[2,3-j]fluoranthene	0.3278	302.11	7.63	0	3282860	57.58
207-18-1	Naphtho[2,3-k]fluoranthene	0.3097	302.11	7.63	0	3148090	60.05
193-09-9	Naphtho[2,3-e]pyrene	0.2759	302.11	7.63	0	2890645	65.39
192-51-8	dibenzo[e,l]pyrene	0.2850	302.11	7.63	0	2960385	63.85
196-42-9	Naphtho[2,3-a]pyrene	0.3431	302.11	7.63	0	3395410	55.67
197-70-6	benzo[b]perylene	0.3431	302.11	7.63	0	3395410	55.67
2113-57-7	PBB 2	0.1954	231.988	4.65	0	2185140	86.51
92-66-0	PBB 3	0.1989	231.99	4.88	0	2300610	82.16
13029-09-9	PBB 4	0.2316	309.9	5.11	0	1899595	99.51
53592-10-2	PBB 7	0.2010	309.9	5.4	0	1759555	107.43
57422-77-2	PBB 9	0.2108	309.9	5.25	0	1796180	105.24

Table S5 (continued)

CASRN	Chemical	Fractional ion abundance	MW (g/mol)	logP	PSA (Å^2)	Response Factor at 500 pg/µL	Estimated LOQ (pg/µL)
59080-32-9	PBB 10	0.2298	309.9	5.07	0	1881620	100.46
92-86-4	PBB 15	0.1906	309.899	5.78	0	1755420	107.68
59080-34-1	PBB 18	0.0897	387.81	5.82	0	744040	254.06
59080-35-2	PBB 26	0.1047	387.81	5.93	0	838400	225.46
115245-07-3	PBB 29	0.1106	387.81	5.97	0	874085	216.26
59080-33-0	PBB 30	0.1065	387.81	5.88	0	846765	223.23
59080-35-3	PBB 31	0.1149	387.81	5.93	0	897620	210.59
60044-24-8	PBB 49	0.1214	465.72	6.68	0	723805	261.16
59080-37-4	PBB 52	0.1242	465.72	6.53	0	736345	256.71
60044-25-9	PBB 53	0.1242	465.72	6.34	0	736785	256.56
77102-82-0	PBB 77	0.0897	465.72	6.54	0	578790	326.59
16400-50-3	PBB 80	0.0989	465.72	6.59	0	622305	303.75
67888-96-4	PBB-101	0.0508	543.63	7.25	0	291045	649.48
59080-39-6	PBB-103	0.0602	543.63	7.15	0	331670	569.92
96551-70-1	PBB-114	0.0687	543.63	7.24	0	364015	519.28
81381-52-4	PBB-137	0.0524	621.54	7.77	0	228010	829.03
120991-47-1	PBB-141	0.0570	621.54	7.61	0	245485	770.02
59080-40-9	PBB-153	0.0544	621.54	7.92	0	231710	815.79
59261-08-4	PBB-155	0.0551	621.54	7.78	0	236530	799.17
77607-09-1	PBB-156	0.0771	621.54	7.79	0	302995	623.87
120991-48-2	PBB-159	0.0519	621.54	7.65	0	228465	827.38
60044-26-0	PBB-169	0.0863	621.54	7.87	0	327285	577.56
67733-52-2	PBB-180	0.0513	699.45	8.34	0	170405	1109.27
88700-06-5	PBB-189	0.0507	699.45	8.29	0	169680	1114.01
69887-11-2	PBB-200	0.0509	777.36	8.7	0	131195	1440.79
6903-63-5	PBDE 11	0.1194	325.894	6.02	9	794855	237.81
147217-71-8	PBDE 8	0.3306	325.894	5.8	9	1656785	114.09
147217-75-2	PBDE 17	0.1295	403.8	6.53	9	634495	297.92
147217-78-5	PBDE 33	0.1440	403.804	6.62	9	688200	274.67
189084-63-7	PBDE 75	0.0988	481.71	7.34	9	392790	481.24
189084-62-6	PBDE 71	0.1212	481.71	7.3	9	456925	413.69
189084-61-5	PBDE 66	0.1258	481.71	7.47	9	468695	403.31
189084-64-8	PBDE 100	0.0634	559.63	8.03	9	211020	895.78
446254-80-4	PBDE 118	0.0936	559.63	8.27	9	278130	679.64
207122-15-4	PBDE 154	0.0523	637.54	8.83	9	133915	1411.52
68631-49-2	PBDE 153	0.0650	637.54	8.98	9	155390	1216.46
60348-60-9	PBDE 99	0.0821	559.63	8.19	9	253510	745.63
182677-30-1	PBDE 138	0.0613	637.536	8.82	9	150810	1253.4
189084-58-0	PBDE 166	0.0753	637.54	8.62	9	178385	1059.66
51452-87-0	PBDE 4	0.3609	325.894	5.68	9	1749905	108.02
147217-72-9	PBDE 6	0.4110	325.89	5.85	9	1954490	96.71
33513-66-3	PBDE 9	0.3908	325.89	5.88	9	1887665	100.14
147217-73-0	PBDE 19	0.1529	403.8	6.37	9	713745	264.84
337513-67-4	PBDE 21	0.1627	403.8	6.44	9	748870	252.42
337513-75-4	PBDE 26	0.1883	403.8	6.78	9	843540	224.09
337513-53-8	PBDE 27	0.1836	403.8	6.54	9	821490	230.1
189084-57-9	PBDE 51	0.1022	481.71	7.22	9	403335	468.66
446254-33-7	PBDE 62	0.1551	481.71	7.11	9	549590	343.94
327185-09-1	PBDE 69	0.1497	481.71	7.34	9	533945	354.02
182346-21-0	PBDE 85	0.0779	559.63	8.02	9	245880	768.78
446254-55-3	PBDE 88	0.1042	559.63	7.84	9	307610	614.5
446254-56-4	PBDE 89	0.0916	559.63	7.86	9	279355	676.65
446254-67-7	PBDE 103	0.1065	559.63	8.11	9	308510	612.71

Table S5 (continued)

CASRN	Chemical	Fractional ion abundance	MW (g/mol)	logP	PSA (Å^2)	Response Factor at 500 pg/µL	Estimated LOQ (pg/µL)
446254-71-3	PBDE 108	0.1212	559.63	8.2	9	337990	559.26
446254-78-0	PBDE 115	0.0940	559.63	8.01	9	282620	668.83
446254-86-0	PBDE 127	0.0561	559.63	8.37	9	189565	997.15
182677-28-7	PBDE 128	0.0975	637.54	8.66	9	215230	878.26
446254-98-4	PBDE 142	0.0837	637.54	8.46	9	195440	967.18
446255-00-1	PBDE 144	0.0731	637.54	8.77	9	172405	1096.42
35854-94-5	PBDE 155	0.0500	637.54	8.67	9	131320	1439.43
446255-11-4	PBDE 160	0.1033	637.54	8.63	9	225185	839.43
405237-86-7	PBDE 185	0.0653	715.45	9.39	9	116620	1620.9
117964-21-3	PBDE 201	0.0524	793.36	10	9	72475	2608.15
NO CAS	2'-Hydroxy-4-monobromodiphenyl ether	0.1241	263.978	4.25	29	714710	264.48
NO CAS	2'-Hydroxy-2,4,4'-tribromodiphenyl ether	0.1012	419.799	6.13	29	408155	463.13
NO CAS	2'-Methoxy-2,4,4'-tribromodiphenyl ether	0.0668	433.815	5.53	18	307570	614.58
NO CAS	3-Methoxy-2,2',4,4',6-pentabromodiphenyl ether	0.0427	589.636	7.32	18	133355	1417.46
578-57-4	2-Bromoanisole	0.1602	185.97	2.7	9	1039805	181.79
2398-37-0	3-Bromoanisole	0.1709	185.968	3.03	9	1173710	161.05
104-92-7	4-Bromoanisole	0.1708	185.968	3.16	9	1207545	156.54
95970-08-4	2,5-Dibromoanisole	0.1786	263.878	3.62	9	1038525	182.01
21702-84-1	2,4-Dibromoanisole	0.1592	263.878	3.69	9	962985	196.29
95970-22-2	2,3-Dibromoanisole	0.1754	263.878	3.46	9	1002490	188.56
38603-09-7	2,6-Dibromoanisole	0.1551	263.878	3.23	9	887415	213.01
74137-36-3	3,5-Dibromoanisole	0.1708	263.878	3.87	9	1039650	181.82
95970-10-8	2,4,5-Tribromoanisole	0.1160	341.789	4.48	9	650535	290.57
106-41-2	4-Bromophenol	0.2641	171.95	2.49	20	1317345	143.49
591-20-8	3-Bromophenol	0.1991	171.95	2.63	20	1106065	170.9
615-58-7	2,4-Dibromophenol	0.1452	249.86	3.43	20	796420	237.35
615-58-7	2,5-Dibromophenol	0.2529	249.863	3.43	20	1200570	157.45
608-33-3	2,6-Dibromophenol	0.2419	249.863	3.41	20	1158225	163.2
626-41-5	3,5-Dibromophenol	0.2459	249.863	3.73	20	1229910	153.69
57383-80-9	2,3-Dibromophenol	0.2402	249.863	3.48	20	1164410	162.34
118-79-6	2,4,6-Tribromophenol	0.1280	327.773	4.33	20	634550	297.89
138507-65-0	2,3,4-Tribromophenol	0.1363	327.773	4.31	20	663575	284.86
2039-86-3	3-Bromostyrene	0.1766	181.973	3.53	0	2037555	92.77
2039-82-9	4-Bromostyrene	0.1694	181.973	3.59	0	2003175	94.36
1522-92-5	Tribromoneopentyl alcohol	0.1634	321.82	2.47	20	657370	287.55
3278-89-5	2,4,6-Tribromophenyl allyl ether	0.0619	367.8	4.97	9	389395	485.44
3322-93-8	1,2-Dibromo-4-(1,2-dibromoethyl)cyclohexane	0.1363	423.767	4.44	0	874520	216.15
39569-21-6	Tetrabromo-o-chlorotoluene	0.0553	437.665	5.64	0	440855	428.78
87-83-2	Pentabromotoluene	0.0441	481.61	5.87	0	327645	576.93
85-22-3	Pentabromoethylbenzene	0.0394	495.63	6.4	0	287370	657.78
35109-60-5	(2,3-Dibromopropyl) (2,4,6-tribromophenyl) ether	0.1164	525.641	5.92	9	399420	473.26
20566-35-2	Tetrabromophthalate diol	0.0609	623.762	1.15	102	186445	1013.84
3194-55-6	1,2,5,6,9,10-Hexabromocyclododecane	0.0993	635.65	6.63	0	384380	491.77
37853-59-1	1,2-Bis(2,4,6-tribromophenoxy)ethane	0.1269	681.562	7.88	18	219960	859.37
262-12-4	Dibenzo-p-dioxin	0.1797	184.052	4.38	18	1487410	127.08
262-16-8	1,4-Dioxino(2,3,b,5,6,b')dipyridine	0.4026	186.04	1.6	44	1239765	152.47
39227-54-8	2-Chlorodibenzo-p-dioxin	0.2648	218.013	5.1	18	1906920	99.13
39227-53-7	1-Chlorodibenzo-p-dioxin	0.2732	218.013	4.88	18	1874595	100.84
50585-39-2	1,3-Dichlorodibenzo-p-dioxin	0.1936	251.974	5.61	18	1374780	137.5
54536-19-5	1,4-Dichlorodibenzo-p-dioxin	0.2008	251.974	5.31	18	1351470	139.87
38178-38-0	1,6-Dichlorodibenzo-p-dioxin	0.2056	251.974	5.38	18	1389325	136.06
33857-26-0	2,7-Dichlorodibenzo-p-dioxin	0.2218	251.974	5.82	18	1568410	120.52
38964-22-6	2,8-Dichlorodibenzo-p-dioxin	0.2027	251.974	5.82	18	1467355	128.82

Table S5 (continued)

CASRN	Chemical	Fractional ion abundance	MW (g/mol)	logP	PSA (Å^2)	Response Factor at 500 pg/µL	Estimated LOQ (pg/µL)
29446-15-9	2,3-Dichlorodibenzo-p-dioxin	0.2150	251.974	5.69	18	1503440	125.73
54536-18-4	1,2-Dichlorodibenzo-p-dioxin	0.2044	251.974	5.47	18	1401765	134.85
39227-58-2	1,2,4-Trichlorodibenzo-p-dioxin	0.1481	285.935	5.91	18	990060	190.92
54536-17-3	1,2,3-Trichlorodibenzo-p-dioxin	0.1505	285.935	6.08	18	1021905	184.98
82306-65-8	1,7,8-Trichlorodibenzo-p-dioxin	0.1614	285.935	6.19	18	1090050	173.41
33857-28-2	2,3,7-Trichlorodibenzo-p-dioxin	0.1625	285.935	6.41	18	1124050	168.17
33423-92-6	1,3,6,8-Tetrachlorodibenzo-p-dioxin	0.1371	319.896	6.84	18	870400	217.17
116889-70-4	1,3,7,9-Tetrachlorodibenzo-p-dioxin	0.1297	319.896	6.84	18	835100	226.35
50585-46-1	1,3,7,8-Tetrachlorodibenzo-p-dioxin	0.1329	319.896	7.01	18	863385	218.94
67323-56-2	1,2,6,8-Tetrachlorodibenzo-p-dioxin	0.1326	319.896	6.71	18	839190	225.25
30746-58-8	1,2,3,4-Tetrachlorodibenzo-p-dioxin	0.1290	319.896	6.4	18	800055	236.27
41903-57-5	1,2,6,7-Tetrachlorodibenzo-p-dioxin	0.1414	319.896	6.4	18	856255	220.76
34816-53-0	1,2,7,8-Tetrachlorodibenzo-p-dioxin	0.1306	319.896	6.79	18	835555	226.23
116889-69-1	1,2,8,9-Tetrachlorodibenzo-p-dioxin	0.1447	319.896	6.57	18	883890	213.86
71998-76-0	1,2,4,6,8/1,2,4,7,9-Pentachlorodibenzo-p-dioxin	0.1164	353.857	7.15	18	668690	282.68
58802-08-7	1,2,4,7,8-Pentachlorodibenzo-p-dioxin	0.1311	353.857	7.23	18	733890	257.57
39227-61-7	1,2,3,4,7-Pentachlorodibenzo-p-dioxin	0.1237	353.857	7.11	18	697710	270.93
40321-76-4	1,2,3,7,8-Pentachlorodibenzo-p-dioxin	0.1371	353.857	7.39	18	766520	246.61
71925-18-3	1,2,3,8,9-Pentachlorodibenzo-p-dioxin	0.1475	353.857	7.18	18	798035	236.87
39227-62-8	1,2,4,6,7,9/1,2,4,6,8,9-Hexachlorodibenzo-p-dioxin	0.1185	387.818	7.45	18	587965	321.49
39227-28-6	1,2,3,4,7,8-Hexachlorodibenzo-p-dioxin	0.1085	387.818	7.71	18	556745	339.52
57653-85-7	1,2,3,6,7,8-Hexachlorodibenzo-p-dioxin	0.0963	387.818	7.78	18	511500	369.55
58200-66-1	1,2,3,4,6,7-Hexachlorodibenzo-p-dioxin	0.1095	387.818	7.49	18	555480	340.3
19408-74-3	1,2,3,7,8,9-Hexachlorodibenzo-p-dioxin	0.1127	387.818	7.78	18	574250	329.17
50585-41-6	2,3,7,8-Tetrabromodibenzo-p-dioxin	0.1112	495.694	8.24	18	358935	526.63
58200-70-7	1,2,3,4,6,7,9-Heptachlorodibenzo-p-dioxin	0.0929	421.779	8.1	18	429620	439.99
35822-46-9	1,2,3,4,6,7,8-Heptachlorodibenzo-p-dioxin	0.0911	421.779	8.1	18	423440	446.41
3268-87-9	1,2,3,4,6,7,8,9-Octachlorodibenzo-p-dioxin	0.0809	455.74	8.41	18	334545	565.02
132-64-9	Dibenzofuran	0.3597	168.057	4.12	13	2691250	70.24
51230-49-0	2-Chlorodibenzofuran	0.3613	202.02	4.66	13	2532495	74.64
74992-96-4	4-Chlorodibenzofuran	0.3605	202.018	4.56	13	2477865	76.29
5409-83-6	2,8-Dichlorodibenzofuran	0.2604	235.979	5.29	13	1865825	101.31
5410-97-9	3-Nitrodibenzofuran	0.2152	213.042	3.85	59	1087840	173.76
54589-71-8	2,4,8-Trichlorodibenzofuran	0.1876	269.94	5.79	13	1325245	142.64
30402-14-3	1,3,6,8-Tetrachlorodibenzofuran	0.1770	303.901	6.1	13	1116025	169.38
24478-72-6	1,2,3,4-Tetrachlorodibenzofuran	0.1606	303.901	6.1	13	1038875	181.95
57117-41-6	1,2,3,7,8-Pentachlorodibenzofuran	0.1642	337.862	6.73	13	951905	198.58
57117-31-4	2,3,4,7,8-PeCDF	0.1625	337.862	6.8	13	949825	199.01
55684-94-1	1,2,3,4,7,8-Hexachlorodibenzofuran	0.1276	371.823	6.7	13	678110	278.76
38998-75-3	1,2,3,4,6,7,8-Heptachlorodibenzofuran	0.0974	405.784	7.26	13	492075	384.14
39001-02-0	1,2,3,4,6,7,8,9-Octachlorodibenzofuran	0.0798	439.745	7.22	13	370160	510.67
7005-72-3	4-Chlorophenyl phenyl ether	0.2078	204.034	4.81	9	1818410	103.95
620-88-2	4-nitrophenyl phenyl ether	0.2285	215.058	3.93	55	1158475	163.17
2303-23-3	2-chlorophenyl-4-nitrophenyl ether	0.1309	249.02	4.65	55	750415	251.9
2303-23-3	3-chlorophenyl-4-nitrophenyl ether	0.1576	249.02	4.65	55	860410	219.69
1836-74-4	4-chlorophenyl-4-nitrophenyl ether	0.1803	249.019	4.53	55	933605	202.47
2093-28-9	2,6-dichlorophenyl-4-nitrophenyl ether	0.0911	282.98	4.7	55	503435	375.48
391-48-7	2,5-dichlorophenyl-4-nitrophenyl ether	0.1214	282.98	4.7	55	622610	303.6
21105-77-1	3,5-Dichlorophenyl-4-nitrophenyl ether	0.1069	282.98	5.37	55	613710	308.01
82239-20-1	2,3-Dichlorophenyl-4-nitrophenyl ether	0.1355	282.98	4.9	55	691265	273.45
22532-80-5	3,4-dichlorophenyl-4-nitrophenyl ether	0.1239	282.98	5.12	55	664235	284.58
142022-58-0	2,3,6-Trichlorophenyl-4-nitrophenyl ether	0.0907	316.941	5.22	55	465350	406.2
142022-59-1	2,3,5-Trichlorophenyl-4-nitrophenyl ether	0.1158	316.941	5.56	55	575200	328.63

Table S5 (continued)

CASRN	Chemical	Fractional ion abundance	MW (g/mol)	logP	PSA (Å^2)	Response Factor at 500 pg/µL	Estimated LOQ (pg/µL)
22532-68-9	2,4,5-Trichlorophenyl-4-nitrophenyl ether	0.0708	316.941	5.44	55	395500	477.95
2671-93-4	2,4-Dibromophenyl-4-nitrophenyl ether	0.0978	370.879	5.52	55	413175	457.5
142022-59-1	3,4,5-Trichlorophenyl-4-nitrophenyl ether	0.0849	316.941	5.56	55	457200	413.45
142022-61-5	2,3,4-Trichlorophenyl-4-nitrophenyl ether	0.1061	316.941	9.52	101	698045	270.8
120-83-2	2,4-Dichlorophenol	0.2330	161.964	2.99	20	1410075	134.05
87-65-0	2,6-Dichlorophenol	0.2339	161.964	2.61	20	1282655	147.37
16766-30-6	4-Chloroguaiacol	0.1986	158.013	2.36	29	1014925	186.25
88-06-2	2,4,6-Trichlorophenol	0.1594	195.925	3.58	20	1062225	177.95
95-95-4	2,4,5-Trichlorophenol	0.1804	195.925	3.71	20	1196250	158.02
3978-67-4	3,4-Dichlorocatechol	0.1763	177.959	2.9	40	940260	201.04
77102-94-4	3,4-Dichloroguaiacol	0.1587	191.974	3.1	29	913090	207.02
2138-22-9	4-Chlorocatechol	0.3014	143.998	2.15	40	1292150	146.29
16766-31-7	4,6-Dichloroguaiacol	0.1343	191.974	3.16	29	817430	231.25
2460-49-3	4,5-Dichloroguaiacol	0.1661	191.974	3.25	29	975090	193.86
18268-76-3	6-Chlorovanillin	0.1828	186.008	2.29	47	797395	237.06
58-90-2	2,3,4,6-Tetrachlorophenol	0.1406	229.886	4.17	20	940690	200.94
60712-44-9	3,4,6-Trichloroguaiacol	0.1092	225.935	3.83	29	703085	268.85
19463-48-0	5-Chlorovanillin	0.1947	186.008	2	47	783210	241.35
87-86-5	Pentachlorophenol	0.1180	263.847	4.78	20	787955	239.9
76341-69-0	2-Chlorosyringaldehyde	0.1753	216.019	1.92	56	650905	290.41
18268-69-4	5,6-Dichlorovanillin	0.1295	219.969	2.97	47	643815	293.6
2539-17-5	Tetrachloroguaiacol	0.1096	259.896	4.6	29	695200	271.9
2539-26-6	Trichlorosyringol	0.0681	255.946	3.86	39	425190	444.57
76330-06-8	2,6-Dichlorosyringaldehyde	0.1020	249.98	2.98	56	482395	391.85
136-94-7	Zinc diethyldithiocarbamate	0.1828	359.98		121	0	
13927-77-0	Nickel dibutyldithiocarbamate	0.2228	466.111		121	0	
134-32-7	1-naphthylamine	0.3403	143.07	2.17	26	1532630	123.34
6265-91-4	2-(4-chlorophenyl)benzothiazole	0.2333	245.01	5.02	41	1302590	145.12
4074-77-5	2-benzothiazolyl sulfide	0.1583	299.99	6.01	108	726900	260.05
120-72-9	indole	0.3391	117.06	2.14	16	1798700	105.09
95-14-7	benzotriazole	0.1990	119.05	1.34	42	785735	240.57
84-54-8	2-methyl-9,10-anthraquinone	0.1857	222.07	3.84	34	1030765	183.39
95-33-0	N-Cyclohexyl-2-benzothiazolylsulfenamide	0.2108	264.07	5.32	78	1034500	182.72
128-37-0	butylated hydroxytoluene	0.2338	220.18	5.32	20	1758370	107.5
149-30-4	2-mercaptobenzothiazole	0.2150	166.99	2.38	69	908170	208.14
102-77-2	2-(morpholinothio)benzothiazole	0.2869	252.04	2.44	79	897060	210.72
95-32-9	4-(2-benzothiazolyldithio)morpholine	0.1762	284.01	3.48	104	633755	298.27
90-00-6	2-ethylphenol	0.3628	122.07	2.47	20	1991875	94.9
697-82-5	2,3,5-trimethylphenol	0.2344	136.09	2.86	20	1528765	123.65
620-17-7	3-ethylphenol	0.3366	122.07	2.47	20	1884280	100.32
489-01-0	2,6-ditertbutyl-4-methoxyphenol	0.1860	236.18	4.69	29	1154390	163.75
94-86-0	propenyl guaethol	0.1399	178.1	2.9	29	835985	226.11
585-34-2	3-tert-butylphenol	0.2583	150.1	3.17	20	1681925	112.39
91-10-1	2,6-dimethoxyphenol	0.2198	154.06	0.77	39	687655	274.89
86-74-8	carbazole	0.4290	167.07	3.72	16	2698270	70.06
732-26-3	quinoline	0.3942	129.06	2.08	13	1952035	96.84
92-69-3	4-hydroxybiphenyl	0.3004	170.07	3.2	20	1734015	109.01
2440-22-4	Drometrizole	0.2550	225.09	4.3	51	1303575	145.01
135-88-6	n-phenyl-2-naphthylamine	0.2498	219.1	4.2	12	1645700	114.86
97-02-9	2,4-dinitroaniline	0.1480	183.03	2.22	118	578280	326.88
99-09-2	3-nitroaniline	0.2428	138.04	1.37	72	806800	234.29
1817-73-8	2-bromo-4,6-dinitroaniline	0.0981	260.94	3.09	118	407205	464.21
100-01-6	4-nitroaniline	0.2067	138.04	1.39	72	720555	262.34

Table S5 (continued)

CASRN	Chemical	Fractional ion abundance	MW (g/mol)	logP	PSA (Å^2)	Response Factor at 500 pg/µL	Estimated LOQ (pg/µL)
121-87-9	2-chloro-4-nitroaniline	0.1416	172	2.46	72	664865	284.31
3531-19-9	2-chloro-4,6-dinitroaniline	0.1021	216.99	3.38	118	502255	376.36
89-63-4	4-chloro-2-nitroaniline	0.1298	172	2.74	72	666975	283.41
827-94-1	2,6-dibromo-4-nitroaniline	0.0654	293.86	3.47	72	315465	599.2
643-28-7	2-isopropylaniline	0.3490	135.1	2.28	26	1657660	114.03
108-44-1	m-toluidine	0.3213	107.07	1.4	26	1274555	148.31
108-69-0	3,5-dimethylaniline	0.2631	121.09	1.86	26	1236320	152.9
554-00-7	2,4-dichloroaniline	0.2349	162	2.74	26	1273705	148.41
95-64-7	3,4-dimethylaniline	0.2034	121.09	1.86	26	1022220	184.92
634-93-5	2,4,6-trichloroaniline	0.1735	196.46	3.74	26	1117420	169.16
90-30-2	N-phenyl-1-naphthylamine	0.2235	219.1	4.2	12	1515815	124.7
101-77-9	4,4'-methylenedianiline	0.1924	198.12	1.64	52	689895	273.99
95-53-4	o-toluidine	0.3415	107.07	1.4	26	1333295	141.77
579-66-8	2,6-diethylaniline	0.3083	149.12	2.92	26	1720290	109.88
95-78-3	2,5-dimethylaniline	0.2232	121.09	1.86	26	1094870	172.65
87-59-2	2,3-dimethylaniline	0.2170	121.09	1.86	26	1072305	176.28
583-78-8	2,5-dichlorophenol	0.2231	163	2.88	20	1322055	142.98
95-56-7	2-bromophenol	0.2056	173	2.47	20	1085830	174.09
5989-27-5	d-limonene	0.1723	136.13	4.45	0	3318690	56.96
5392-40-5	Citral B	0.1807	152.12	3.17	17	1313850	143.87
5392-40-5	Citral A	0.2562	152.12	3.17	17	1700780	111.14
106-22-9	b-citronellol	0.1377	156.15	3.38	20	1086155	174.03
107-75-5	hydroxy-citronellal	0.2938	172.15	1.54	37	1021945	184.97
79-77-6	b-ionone	0.2256	192.15	3.86	17	1521800	124.21
106-24-1	geraniol	0.2768	154.14	3.28	20	1789075	105.66
4602-84-0	farnesol I	0.2038	222.2	5.31	20	1567890	120.56
4602-84-0	farnesol II	0.2134	222.2	5.31	20	1622225	116.52
4602-84-0	farnesol III	0.2577	222.2	5.31	20	1864775	101.37
4602-84-0	farnesol IV	0.2040	222.2	5.31	20	1569310	120.45
101-85-9	amylcinnamyl alcohol	0.1163	204.15	4.37	20	953195	198.31
122-40-7	amyl cinnamal	0.0962	202.14	4.8	17	937340	201.66
93-15-2	methyleugenol	0.1593	178.1	2.97	18	1012275	186.74
118-58-1	benzyl salicylate	0.6382	228.08	4.01	47	2445425	77.3
97-54-1	isoeugenol	0.2000	164.08	2.45	29	1024060	184.59
105-13-5	anisyl alcohol	0.1262	138.07	0.95	29	512145	369.09
104-54-1	cinnamyl alcohol	0.1294	134.07	1.7	20	700170	269.97
127-41-3	a-ionone	0.1778	192.15	3.86	17	1275760	148.17
103-41-3	benzyl cinnamate	0.1803	238.1	3.65	26	962655	196.36
100-51-6	benzyl alcohol	0.2134	108.06	1.03	20	859965	219.81
105-95-3	Ethylene brassylate	0.0841	270.18	2.9	53	396780	476.4
31906-04-4	Lyral	0.0785	210.16	2.53	37	438390	431.19
80-54-6	lilial	0.1096	204.15	4.07	17	882620	214.17
104-55-2	cinnamal	0.2356	132.06	2.12	17	1280395	147.63
111-12-6	methyl 2-octynoate	0.0842	154.1	3.56	26	766425	246.64
622-62-8	4-ethoxyphenol	0.4002	138.07	1.84	29	1564330	120.84
843-55-0	bisphenol Z	0.1555	268.15	4.65	40	830895	227.5
92-04-6	4-hydroxy-3-chlorobiphenyl	0.2834	204.03	4	20	1710855	110.49
2081-08-5	bisphenol E	0.3431	212.08	3.08	40	1422190	132.91
1478-61-1	bisphenol AF	0.1984	336.06	2.82	40	678310	278.67
609-19-8	3,4,5-trichlorophenol	0.1724	197.45	4.02	20	1225805	154.21
554-84-7	3-nitrophenol	0.2351	139.03	1.93	66	940745	200.93
98-87-4	2,5-dimethylphenol	0.2319	122.07	2.4	20	1398425	135.17
87-26-3	2-(1-methylbutyl)phenol	0.4456	164.12	3.88	20	2828300	66.83

Table S5 (continued)

CASRN	Chemical	Fractional ion abundance	MW (g/mol)	logP	PSA (Å^2)	Response Factor at 500 pg/µL	Estimated LOQ (pg/µL)
95-65-8	3,4-dimethylphenol	0.2872	122.07	2.4	20	1637870	115.41
644-35-9	2-propylphenol	0.4801	136.09	3.01	20	2716870	69.58
576-26-1	2,6-dimethylphenol	0.2099	122.07	2.4	20	1298870	145.53
88-69-7	2-isopropylphenol	0.3552	136.09	2.82	20	2054400	92.01
94-71-3	2-ethoxyphenol	0.4052	138.07	1.72	29	1523785	124.05
599-64-4	4-alpha-cumylphenol	0.2982	212.12	4.17	20	1770895	106.74
99-07-0	3-(dimethylamino)phenol	0.3034	137.08	1.56	23	1222480	154.63
123-30-8	4-aminophenol	0.3693	109.05	-0.29	46	702300	269.15
91-68-9	N,N-diethyl-3-aminophenol	0.3147	165.12	2.62	23	1547635	122.14
150-76-5	4-methoxyphenol	0.2861	124.05	1.31	29	1071980	176.33
123-07-9	4-ethylphenol	0.4623	122.07	2.47	20	2382590	79.34
615-74-7	6-chloro-m-cresol	0.2828	142.02	2.5	20	1541090	122.66
526-75-0	2,3-xylenol	0.2431	122.07	2.4	20	1447870	130.56
93-51-6	2-methoxy-4-methylphenol	0.2165	138.07	1.65	29	939080	201.29
96-76-4	2,4-di-tert-butylphenol	0.3693	206.17	4.86	20	2441990	77.41
4130-42-1	2,6-di-tert-butyl-4-ethylphenol	0.2980	234.2	5.85	20	2128175	88.82
95-85-2	2-amino-4-chlorophenol	0.2459	143.01	1.67	46	949050	199.17
95-84-1	2-amino-p-cresol	0.2697	123.07	0.9	46	832920	226.95
609-23-4	2,4,6-triiodophenol	0.1563	471.73	3.88	20	513685	367.98
133-53-9	2,4-dichloro-3,5-dimethylphenol	0.1764	191	3.91	20	1255190	150.6
88-04-0	4-chloro-3,5-dimethylphenol	0.2239	156.03	3.35	20	1544435	122.39
2772-45-4	2,4-bis(alpha,alpha-dimethylbenzyl)phenol	0.2252	330.2	6.85	20	1173810	161.04
42486-53-3	4-amino-2,6-dichlorophenol	0.1543	178	1.4	46	586375	322.37
119-33-5	2-nitro-p-cresol	0.1995	153.04	2.17	66	855400	220.98
89-72-5	2-sec-butylphenol	0.3754	150.1	3.35	20	2329670	81.14
88-27-7	2,6-di-tert-butyl-4-dimethylaminomethylphenol	0.2516	263.23	4.62	23	1321695	143.02
95-55-6	2-aminophenol	0.3579	109.05	0.44	46	888455	212.76
527-60-6	2,4,6-trimethylphenol	0.2607	136.09	2.86	20	1653965	114.29
580-51-8	3-hydroxybiphenyl	0.3909	170.07	3.23	20	2122365	89.06
108-43-0	3-chlorophenol	0.3235	128.56	2.4	20	1741280	108.56
90-05-1	guaiacol	0.2719	124.05	1.19	29	993155	190.33
2051-60-7	PCB 1	0.2898	188	4.45	0	3504705	53.94
34883-43-7	PCB 8	0.2258	222	5.02	0	2723150	69.41
33146-45-1	PCB 10	0.2075	222	4.95	0	2526595	74.82
38444-84-7	PCB 20	0.1749	256	5.46	0	2035660	92.86
55702-46-0	PCB 21	0.1696	256	5.35	0	1958775	96.5
38444-90-5	PCB 37	0.1844	256	5.56	0	2147665	88.02
52663-59-9	PCB 41	0.1102	290	5.83	0	1287750	146.79
70362-46-8	PCB 43	0.1086	290	5.88	0	1280900	147.57
62796-65-0	PCB 50	0.1155	290	6.02	0	1362345	138.75
74338-24-2	PCB 55	0.1664	290	5.92	0	1763995	107.16
41464-43-1	PCB 56	0.1680	290	5.91	0	1774455	106.53
32598-10-0	PCB 66	0.1696	290	6.05	0	1815920	104.09
32598-13-3	PCB 77	0.1726	290	6	0	1828880	103.36
70362-50-4	PCB 81	0.0864	290	6.01	0	1098085	172.14
52663-62-4	PCB 82	0.0977	324	6.27	0	1051840	179.71
65510-45-4	PCB 85	0.1154	324	6.41	0	1204310	156.96
68194-07-0	PCB 90	0.1106	324	6.46	0	1172305	161.24
38380-01-7	PCB 99	0.1277	324	6.47	0	1304340	144.92
39485-83-1	PCB 100	0.1199	324	6.6	0	1258805	150.16
56558-16-8	PCB 104	0.1412	324	6.51	0	1410080	134.05
32598-14-4	PCB 105	0.1549	324	6.36	0	1490910	126.79
70424-69-0	PCB 106	0.1515	324	6.3	0	1458580	129.6

Table S5 (continued)

CASRN	Chemical	Fractional ion abundance	MW (g/mol)	logP	PSA (Å^2)	Response Factor at 500 pg/µL	Estimated LOQ (pg/µL)
74472-37-0	PCB 114	0.1552	324	6.3	0	1485105	127.28
57465-28-8	PCB 126	0.1591	324	6.45	0	1532265	123.36
38380-07-3	PCB 128	0.0934	358	6.73	0	904020	209.1
38411-22-2	PCB 136	0.1193	358	6.65	0	1078050	175.34
35065-28-2	PCB 138	0.1058	358	6.78	0	994350	190.1
35065-27-1	PCB 153	0.1134	358	6.83	0	1049770	180.07
38380-08-4	PCB 156	0.1349	358	6.74	0	1186855	159.27
39635-35-3	PCB 159	0.1300	358	6.88	0	1164875	162.27
32774-16-6	PCB 169	0.1381	358	6.9	0	1219945	154.95
35065-30-6	PCB 170	0.0799	392	7.11	0	708225	266.9
38411-25-5	PCB 174	0.0800	392	7.07	0	707535	267.16
52663-64-6	PCB 179	0.1023	392	7.03	0	847750	222.98
35065-29-3	PCB 180	0.0905	392	7.16	0	778070	242.94
60145-23-5	PCB 182	0.0838	392	7.3	0	739315	255.68
39635-31-9	PCB 189	0.1131	392	7.2	0	918825	205.73
52663-73-7	PCB 200	0.0902	426	7.38	0	677080	279.18
2051-24-3	PCB 209	0.0770	494	8.1	0	459690	411.2
96-12-8	1,2-Dibromo-3-chloropropane	0.2639	234	2.75	0	1971660	95.87
87-62-7	2,6-Dimethylaniline	0.2703	121	1.86	26	1261540	149.84
2593-15-9	Terrazole	0.1486	246	3.27	63	659680	286.54
2675-77-6	Chloroneb	0.2136	206	3.58	18	1288945	146.65
1918-16-7	Propachlor	0.2714	211	2.28	20	1156335	163.47
13194-48-4	Ethoprophos	0.1343	242	3.59	87	616885	306.42
126-73-8	Tributyl phosphate	0.4936	266	4.26	55	1769520	106.82
1582-09-8	Trifluralin	0.2186	335	5.41	95	770910	245.2
2303-16-4	Diallate I	0.2246	269	5.13	46	1130585	167.19
319-84-6	alpha-BHC	0.1108	288	3.99	0	1056340	178.95
999035-03-9	Diallate II	0.2379	269	5.13	46	1179515	160.26
118-74-1	Hexachlorobenzene	0.1967	282	4.89	0	1833180	103.11
60-51-5	Dimethoate	0.2409	229	1.32	118	639055	295.79
122-34-9	Simazine	0.1445	201	1.19	61	493400	383.11
1912-24-9	Atrazine	0.1783	215	1.53	61	605240	312.32
319-85-7	beta-BHC	0.0925	288	3.99	0	923805	204.62
82-68-8	Pentachloronitrobenzene	0.0708	293	4.16	46	390735	483.77
944-22-9	Fonofos	0.2728	246	3.94	76	1109815	170.32
333-41-5	Diazinon	0.1024	304	3.81	95	422625	447.27
319-86-8	delta-BHC	0.0921	288	3.99	0	921100	205.22
1897-45-6	Chlorothalonil	0.2536	264	2.88	48	923600	204.66
709-98-8	Propanil	0.3212	217	3.18	29	1433960	131.82
298-00-0	Parathion-methyl	0.1943	263	2.78	115	646195	292.52
5598-13-0	Chlorpyrifos Methyl	0.2426	321	3.71	82	775405	243.78
50471-44-8	Vinclozolin	0.0720	285	3.19	47	360875	523.8
76-44-8	Heptachlor	0.0892	370	5.46	0	774785	243.97
15972-60-8	Alachlor	0.1141	269	2.92	30	550195	343.56
122-14-5	Fenitrothion	0.1561	277	3.24	115	563720	335.32
84-65-1	9,10-Anthraquinone	0.2503	208	3.38	34	1244090	151.94
309-00-2	Aldrin	0.0672	362	5.32	0	640940	294.92
51218-45-2	Metolachlor	0.3104	283	3	30	1126795	167.76
90-98-2	4,4'-Dichlorobenzophenone	0.3107	250	4.62	17	1714875	110.23
2921-88-2	Chlorpyrifos	0.0865	349	4.77	82	362335	521.69
56-38-2	Parathion-ethyl	0.1283	291	3.84	115	503435	375.47
1861-32-1	Dacthal	0.1942	330	3.48	53	679410	278.22
465-73-6	Isodrin	0.0910	362	5.32	0	801995	235.7

Table S5 (continued)

CASRN	Chemical	Fractional ion abundance	MW (g/mol)	logP	PSA (Å^2)	Response Factor at 500 pg/µL	Estimated LOQ (pg/µL)
1024-57-3	Heptachlor epoxide	0.0896	386	5.47	13	466775	404.96
40487-42-1	Pendimethalin	0.2599	281	5.56	104	1095825	172.5
5103-74-2	gamma-Chlordane	0.1118	406	5.57	0	816570	231.49
959-98-8	Endosulfan I	0.0253	404	3.13	55	126185	1498.04
60-57-1	Dieldrin	0.0982	378	4.88	13	498535	379.17
72-55-9	4,4'-DDE	0.1400	316	6.37	0	1437080	131.54
19666-30-9	Oxadiazon	0.1559	344	4.55	51	607440	311.19
33213-65-9	Endosulfan II	0.0280	404	3.13	55	135950	1390.43
72-56-0	Perthane	0.4552	306	6.18	0	3536930	53.44
510-15-6	Chlorobenzilate	0.2489	324	4.73	47	941085	200.86
72-54-8	4,4'-DDD	0.2469	318	5.39	0	1981460	95.4
7421-93-4	Endrin aldehyde	0.0549	378	3.19	17	286250	660.36
563-12-2	Ethion	0.2188	384	5.07	171	578520	326.74
1031-07-8	Endosulfan sulfate	0.0610	420	4.3	61	237665	795.35
50-29-3	4,4'-DDT	0.2397	352	5.92	0	1767225	106.96
53494-70-5	Endrin ketone	0.0533	378	2.68	17	273265	691.74
732-11-6	Imidan	0.6227	317	2.84	123	1355215	139.48
72-43-5	Methoxychlor	0.4615	344	4.56	18	1614005	117.12
2385-85-5	Mirex	0.1591	540	7.42	0	680655	277.71
52645-53-1	Permethrin	0.4019	390	7.15	36	1264095	149.54
999046-03-6	Permethrin II	0.4075	390	7.15	36	1277015	148.02
50-32-8	benzo[a]pyrene	0.4780	252	6.4	0	5017750	37.67
78-40-0	Triethyl phosphate	0.1979	182	1.08	55	631625	299.27
91-20-3	naphthalene	0.5333	128	3.45	0	5951200	31.76
208-96-8	acenaphthylene	0.5492	152	4.26	0	6634150	28.49
83-32-9	acenaphthene	0.3017	154	4.19	0	4127975	45.79
86-73-7	Fluorene	0.3608	166	4.16	0	4351985	43.43
877-09-8	Tetrachloro-meta-xylene	0.1401	242	5.23	0	1789550	105.63
115-96-8	TCEP	0.1663	284	0.48	55	474650	398.24
120-12-7	anthracene	0.5339	178	4.68	0	6143550	30.77
85-29-0	2,4'-Dichlorobenzophenone	0.3045	250	4.07	17	1556095	121.48
81-14-1	Musk Ketone	0.2279	294	3.86	109	771775	244.93
120067-83-6	Fipronil-sulfide	0.1911	420	6.4	93	538925	350.75
120068-37-3	Fipronil	0.2512	436	4.76	104	598780	315.69
129-00-0	pyrene	0.5236	202	5.17	0	5833450	32.4
39765-80-5	trans-Nonachlor	0.1398	440	5.9	0	870960	217.03
72-20-8	Endrin	0.0298	378	4.88	13	206300	916.27
120068-36-2	Fipronil-sulfone	0.1588	452	7.44	110	410735	460.22
56-55-3	benz[a]anthracene	0.4845	228	5.91	0	5412500	34.92
218-01-9	chrysene	0.4748	228	5.91	0	5332500	35.45
191-24-2	benzo[ghi]perylene	0.4796	276	6.89	0	4666235	40.51
1090-13-7	5,12-Naphthacene-quinone	0.2638	258.07	4.61	34	1307460	144.58
82-05-3	Benzanthrone	0.3336	230.07	4.81	17	2039355	92.69
486-25-9	9-Fluorenone	0.3994	180.06	3.58	17	2303525	82.06
5737-13-3	4H-cyclopenta[def]phenanthren-4-one	0.3990	204.06	3.96	17	2244825	84.21
10439-39-1	9,10-Anthraquinone-d8	0.1854	216.1	3.38	34	967840	195.31
479-79-8	Benzo(a)fluoren-11-one	0.4132	230.07	4.81	17	2389225	79.12
2498-66-0	Benz(a)anthracene-7,12-dione	0.2157	258.07	4.61	34	1126745	167.76
3074-00-8	Naphthanthrone	0.3843	254.07	5.19	17	2143810	88.17
90-12-0	1-methylnaphthalene	0.3146	142.08	3.91	0	4242275	44.56
573-98-8	1,2-dimethylnaphthalene	0.2622	156.09	4.37	0	3853635	49.05
132-65-0	dibenzothiophene	0.4252	184.035	4.38	28	2613260	72.33
483-65-8	retene	0.1992	234.14	6.48	0	2972440	63.59

Table S5 (continued)

CASRN	Chemical	Fractional ion abundance	MW (g/mol)	logP	PSA (Å^2)	Response Factor at 500 pg/µL	Estimated LOQ (pg/µL)
2381-21-7	1-methylpyrene	0.3243	216.094	5.63	0	4112055	45.97
1705-85-7	6-methylchrysene	0.3092	242.109	6.37	0	3843340	49.18
191-30-0	dibenzo[a,l]pyrene	0.2770	302.109	7.63	0	2898945	65.21
1718-52-1	pyrene-d10	0.3826	212.14	5.17	0	4361935	43.34
939-27-5	2-Ethylnaphthalene	0.3366	156.09	4.44	0	4722135	40.03
581-42-0	2,6-dimethylnaphthalene	0.2539	156.09	4.37	0	3762850	50.24
571-58-4	1,4-dimethylnaphthalene	0.2293	156.09	4.37	0	3490130	54.16
569-41-5	1,8-dimethylnaphthalene	0.2257	156.09	4.37	0	3449285	54.8
59919-41-4	2,6-diethylnaphthalene	0.2687	184.13	5.43	0	4220465	44.79
2531-84-2	2-methylphenanthrene	0.3073	192.09	5.14	0	4152100	45.53
779-02-2	9-methylanthracene	0.2867	192.09	5.14	0	3944320	47.92
1576-67-6	3,6-dimethylphenanthrene	0.2683	206.11	5.6	0	3781315	49.99
613-06-9	2,3-dimethylanthracene	0.2844	206.11	5.6	0	3947790	47.88
781-43-1	9,10-dimethylanthracene	0.1927	206.11	5.6	0	2960265	63.85
205-12-9	benzo[c]fluorene	0.2713	216.09	5.39	0	3447255	54.83
27208-37-3	cyclopenta[cd]pyrene	0.3664	226.08	5.99	0	4517145	41.85
57-97-6	7,12-dimethylbenz[a]anthracene	0.1984	256.13	6.83	0	2718960	69.52
192-97-2	benzo[e]pyrene	0.3747	252.09	6.4	0	4188620	45.13
213-46-7	benzo[a]chrysene	0.3360	278.11	7.14	0	3654135	51.73
191-07-1	coronene	0.3132	300.09	7.38	0	3129780	60.4
189-55-9	dibenzo[a,i]pyrene	0.3013	302.11	7.63	0	3084755	61.28
189-64-0	dibenzo[a,h]pyrene	0.2858	302.11	7.63	0	2966640	63.72
2052-07-5	PBB 1	0.1897	231.988	4.55	0	2101820	89.94
7025-06-1	PBDE 1	0.1884	247.983	4.95	9	1390865	135.91
6876-00-2	PBDE 2	0.1316	247.983	5.11	9	1093190	172.91
101-55-3	PBDE 3	0.1432	247.983	5.11	9	1163445	162.47
51930-04-2	PBDE 10	0.3632	325.894	5.63	9	1750935	107.96
171977-44-9	PBDE 7	0.3047	325.894	5.8	9	1559865	121.18
189084-59-1	PBDE 12	0.1135	325.894	5.88	9	756900	249.74
83694-71-7	PBDE 13	0.1138	325.89	5.8	9	753070	251.01
2050-47-7	PBDE 15	0.1322	325.89	6.01	9	856450	220.71
155999-95-4	PBDE 30	0.1522	403.8	6.44	9	712845	265.17
189084-60-4	PBDE 32	0.1324	403.8	6.53	9	644895	293.11
147217-77-4	PBDE 25	0.1544	403.8	6.17	9	714540	264.54
41318-75-6	PBDE 28	0.1196	403.8	6.7	9	601430	314.3
147217-80-9	PBDE 35	0.0695	403.8	6.4	9	398840	473.94
147217-81-0	PBDE 37	0.0794	403.8	6.78	9	445700	424.11
243982-82-3	PBDE 49	0.1239	481.71	7.47	9	463575	407.76
5436-43-1	PBDE 47	0.0861	481.71	7.39	9	354615	533.05
93703-48-1	PBDE 77	0.0691	481.71	7.55	9	300820	628.37
189084-66-0	PBDE 119	0.0827	559.63	8.11	9	255955	738.51
189084-65-9	PBDE 116	0.1178	559.625	7.72	9	338585	558.29
65075-08-3	PBDE 31	0.1515	403.8	6.78	9	718280	263.17
446254-23-5	PBDE 50	0.1158	481.71	7.17	9	442435	427.24
106-48-9	4-Chlorophenol	0.3314	128.003	2.43	20	1793140	105.42
13674-84-5	TCPP	0.1884	326	1.53	55	565195	334.45
1222-05-5	Galaxolide	0.2268	258.2	6.23	9	1826545	103.49
78-70-6	linalool	0.1435	154.25	3.28	20	1100240	171.81
95-16-9	benzothiazole	0.3097	135.01	2.01	41	1296260	145.83
108-68-9	3,5-dimethylphenol	0.2222	122.07	2.4	20	1354770	139.53
99-89-8	4-isopropylphenol	0.3527	136.09	2.82	20	2043675	92.49
91-59-8	2-naphthylamine	0.3724	143.07	2.17	26	1638545	115.36
88-74-4	2-Nitroaniline	0.2042	138.04	1.83	72	813265	232.43

Table S5 (continued)

CASRN	Chemical	Fractional ion abundance	MW (g/mol)	logP	PSA (Å^2)	Response Factor at 500 pg/µL	Estimated LOQ (pg/µL)
95-51-2	2-chloroaniline	0.3214	127.02	1.91	26	1429925	132.19
95-82-9	2,5-dichloroaniline	0.2345	160.98	2.71	26	1266935	149.2
106-49-0	p-toluidine	0.3878	107.07	1.4	26	1464445	129.08
24544-04-5	2,6-diisopropylaniline	0.3232	177.15	3.61	26	1873925	100.87
578-54-1	2-ethylaniline	0.4008	121.09	1.93	26	1726870	109.46
91-64-5	coumarin	0.2451	146.04	1.39	26	956085	197.71
1806-26-4	4-n-octylphenol	0.6671	206.17	5.66	20	4427170	42.7
1570-64-5	4-chloro-2-methylphenol	0.2223	142.02	2.89	20	1443735	130.93
128-39-2	2,6-Di-tert-butylphenol	0.2940	206.17	4.86	20	2063420	91.61
2416-94-6	2,3,6-trimethylphenol	0.2726	136.09	2.86	20	1709640	110.57
150-19-6	3-methoxyphenol	0.2670	124.05	1.51	29	1086160	174.03
1638-22-8	4-butylphenol	0.5252	150.1	3.54	20	3146575	60.07
98-54-4	4-tert-butylphenol	0.3534	150.1	3.17	20	2120185	89.16
120-95-6	2,4-di-tert-amylphenol	0.4175	234.2	5.92	20	2762885	68.42
101-53-1	4-benzylphenol	0.1851	184.09	3.47	20	1217225	155.29

LOQs determined with average response variation method (see main text and figure S2).

Python code for pulling fractional ion abundance from ChemStation

Python code was used to find what fraction of the total ion abundance the quantification ion is. This assumes that the ion chosen for quantification is the one with the greatest abundance in the total spectrum. This is the default for any quantification data base (.qdb) files generated by AMDIS from a PBM library in Chemstation.

This code opens 1 MSP file at a time and extracts the appropriate data from it. These files are generated when an AMDIS library is made in Chemstation. To use the following code, create a folder with the following directory: “C:\ionfragments” and place all MSP files in that folder. Each MSP file should be named in ascending numerical order (e.g. 1.MSP – n.MSP).

The final output will yield a list of values representing the most abundant ion in the spectra as a fraction of the total abundance of all ions in the spectra. It is possible that if a different version of AMDIS is being used, the formatting of the MSP files may be slightly different and some adjustments may need to be made to the given code.

Python Code: Fractional Ion Abundance

```
i = 1
print('Quant Ion Fractional Abundance')              #1588 is the number of chemicals in the library for this
while (i <= 1588):                                    # iteration of AMDIS R&D. It can be changed to suit
any library.
    filepath = 'C:\ionfragments\{}.MSP'.format(i)
    text = open(filepath, 'r')                        # Create the following directory to put your MSP files:
    text_string = text.read()                         # “C:\ionfragments” OR change “filepath” as you desire.
    new_text = text_string.replace(';','\n')
    s = "Num Peaks:  "
    index = new_text.find(s)
    lastalpha = new_text.find('\n', index)
    firstnum = lastalpha + 1
    new_text = new_text.replace('\n\n', '\n')
    num_in_text = (new_text[firstnum:])
    IonMass = []
    IonAbundance = []
    lines = num_in_text.split('\n')                   # split file into separate lines
    for line in lines:
        split_line = line.split(' ')                  # split line into separate words separated by space
        if len(split_line) == 2:
            IonMass.append(split_line[0])
            IonAbundance.append(split_line[1])
    numIonAbundance = []
    for item in IonAbundance:
        numIonAbundance.append(float(item))
    maximum = max(numIonAbundance)
    maxindex = numIonAbundance.index(max(numIonAbundance))
    total = sum(numIonAbundance)
    Fraction = maximum/total
    print(Fraction)
    i = i + 1
```

In order to making sorting the fractional ion abundance values easier, additional code was written which operates on the same MSP files in order to provide the name and CASRN as listed in the library files. The code will yield chemical name or CASRN in the same order as the fractional ion abundance and can be paired directly.

Python Code: Chemical Name

```
i = 1
print('Name')
while (i <= 1588):
    filepath = 'C:\ionfragments\{}.MSP'.format(i)
    text = open(filepath, 'r')
    for lines in text:
        if 'NAME: ' in lines:
            lines = lines.strip('NAME: ')
            lines = lines.strip()
            print(lines)
    i = i + 1
```

Python Code: CASRN

```
i = 1
print('CAS #')
while (i <= 1588):
    filepath = 'C:\ionfragments\{}.MSP'.format(i)
    text = open(filepath, 'r')
    for lines in text:
        if 'CASNO:' in lines:
            lines = lines.strip('CASNO:')
            lines = lines.strip()
            print(lines)
    i = i + 1
```

R code for obtaining chemical parameters from ACD labs via Chemspider.com

```
# This Script will pull specific physico-chemical properties from ChemSpider for a list of cas numbers
from the DRS library

library(webchem) # also at least: stringr, rvest, xml2, car, and some base packages.
library(rvest)
library(car)
# Note: this token will allow access to Chemspider IDs: 1393aff2-1ee3-4265-8add-4abaa3e19ea0
token = "1393aff2-1ee3-4265-8add-4abaa3e19ea0"


# import real full list of DRS entries.
DRSlist <- read.csv("YOUR FILE HERE", header = T)
DRSlist$CA.S... <- as.character(DRSlist$CA.S...)


# pull chemspider IDs for each CASRN in the list
csids <- sapply(DRSlist$CA.S...,
                function(x) {
                  (tryCatch(get_csid(x, token = token),
                        error = function(err) {
                          print(err)
                        },
                        finally = print(paste("chemical", x, "done-zo"))))
                })


# You'll need this:
# function modified from cs_prop in webchem:
# this function is a truncated so EPIsuite values are not accessed. 3 of 8 test chemicals
# gave errors for episuite values when trying to use cs_prop() so this solution only scrapes
# for ACD values. It should be faster then too!
csprop_acdonly <- function (csid, verbose = TRUE, ...)
{
  foo <- function(csid, verbose) {
    qurl <- paste0("https://www.chemspider.com/Chemical-Structure.",
                   csid, ".html")
    if (verbose)
      message(qurl)
    Sys.sleep(rgamma(1, shape = 10, scale = 1/10))
    h <- try(read_html(qurl), silent = TRUE)
    if (inherits(h, "try-error")) {
      warning("CSID not found... Returning NA.")
      return(NA)
    }
    acd <- do.call(rbind, html_table(xml_find_all(h, "//div[@class=\"column two\"]/table")))
    names(acd) <- c("variable", "val")
    acd$variable <- gsub("^(.*)\\:$", "\\1", acd$variable)
    acd$value <- as.numeric(gsub("^(\\d*\\.?\\d*).*$", "\\1",
                                 acd$val))
```

```
    acd$error <- as.numeric(ifelse(grepl("Â±", acd$val), gsub("^\\d*\\.?\\d*Â±(\\d*\\.?\\d*)\\s.*$",
                                                  "\\1", acd$val), NA))
    acd$unit <- ifelse(grepl("\\s.*\\d*$", acd$val), gsub("^.*\\d*\\s(.*\\d*)$",
                                                  "\\1", acd$val), NA)
    acd$val <- NULL
    out <- acd
    return(out)
  }
  out <- lapply(csid, foo, verbose = verbose)
  out <- setNames(out, csid)
  return(out)
}

# scrape Chemspider for chemical properties using modified function to exclude episuite values
props <- sapply(csids,
               function(x) {
                 (tryCatch(csprop_acdonly(x),
                    error = function(err) {
                      print(err)
                      },
                    finally = print(paste("chemical", x, "done-zo"))))
        })


# pulling kow out of the ACD lists
kow <- sapply(props, function(x) {
  tryCatch(
    if(is.atomic(x) == F) {
      x$value[x$variable == 'ACD/LogP']} else {
        return(NA)
      },
          error = function(err) {
            print(err)
          },
          finally = print(paste("chemical", "x", "done-zo"))
          )
})
PSA <- sapply(props, function(x) {
  tryCatch(
    if(is.atomic(x) == F) {
      x$value[x$variable == 'Polar Surface Area']} else {
        return(NA)
      },
    error = function(err) {
      print(err)
    },
    finally = print(paste("chemical", "x", "done-zo"))
  )
})

# compile data into one frame.
```

```
DRSlistfull <- cbind(DRSlist, csids, kow, PSA
```
